# Design and Synthesis of Boronic Chalcones with Dual Anticancer and Anti-Inflammatory Activity

**DOI:** 10.3390/molecules30143032

**Published:** 2025-07-19

**Authors:** Juliana Romano Lopes, Freddy Humberto Marin-Dett, Rita Alexandra Machado Silva, Rafael Consolin Chelucci, Lucília Saraiva, Maria Emília Sousa, Leonardo Luiz Gomes Ferreira, Adriano Defini Andricopulo, Paula Aboud Barbugli, Jean Leandro Dos Santos

**Affiliations:** 1 School of Pharmaceutical Sciences, São Paulo State University (UNESP), Araraquara 14800-903, SP, Brazil; freddy.m.dett@unesp.br; 2 LAQV/REQUIMTE, Laboratório de Microbiologia, Departamento de Ciências Biológicas, Faculdade de Farmácia, Universidade do Porto, 4050-313 Porto, Portugal; up201904678@edu.fc.up.pt (R.A.M.S.); lucilia.saraiva@ff.up.pt (L.S.); 3 Laboratory of Medicinal and Computational Chemistry, Physics Institute of São Carlos, University of São Paulo (USP), São Carlos 13563-120, SP, Brazil; rafaelchelucci@gmail.com (R.C.C.); leonardo@ifsc.usp.br (L.L.G.F.); aandrico@ifsc.usp.br (A.D.A.); 4 Laboratório de Química Orgânica e Farmacêutica, Departamento de Ciências Químicas, Faculdade de Farmácia, Universidade do Porto, 4050-313 Porto, Portugal; esousa@ff.up.pt; 5 CIIMAR—Centro Interdisciplinar de Investigação Marinha e Ambiental, Terminal de Cruzeiros do Porto de Leixôes, 4450-208 Matosinhos, Portugal; 6 School of Dentistry, São Paulo State University (UNESP), Araraquara 14801-385, SP, Brazil; paula.barbugli@unesp.br

**Keywords:** chalcones, boronic acid, head and neck cancer, anti-inflammatory

## Abstract

Head and neck cancer (HNC) is a highly aggressive malignancy with limited treatment options and poor prognosis. Inflammation plays a critical role in HNC progression, with elevated levels of pro-inflammatory cytokines such as TNF, IL-6, IL-8, and IL-1β contributing to tumor development. In this study, a novel series of boronic chalcones was designed and synthesized as potential dual-action anticancer and anti-inflammatory agents. The most potent compounds were evaluated for their cytotoxicity against Squamous Cell Carcinoma (SCC-25), and their selectivity index (SI) was determined. Compound **5** emerged as the most promising, displaying cytotoxicity against cancer cells, with IC_50_ values of 17.9 µM and a favorable SI (>3). Mechanistic studies revealed that its anticancer activity was independent of p53 status, and annexin V/PI staining indicated cell death via necrosis. Interestingly, compound **5** also significantly reduced pro-inflammatory cytokine levels, as TNF and IL-6. Furthermore, drug metabolism and pharmacokinetics (DMPK) studies demonstrated that compound **5** exhibited moderate solubility and high permeability. These findings underscore the crucial role of the boronic acid moiety in enhancing both anticancer and anti-inflammatory properties.

## 1. Introduction

Head and neck cancer (HNC) is the sixth most common cancers worldwide, with a consistently increasing incidence and a predicted 30% annual rise by 2030 [1,2]. Approximately 90% of HNCs are squamous cell carcinomas, originating from the epithelium of the oral cavity, pharynx, and larynx [1].

Histologically, HNC progression begins with epithelial hyperplasia, followed by dysplasia, carcinoma in situ, and invasive carcinoma [1]. The etiology of HNC varies, with two major subtypes: HPV-positive and HPV-negative tumors, each displaying distinct gene expression and immune profiles [3]. HPV-negative HNC, the predominant form, is strongly associated with tobacco use and alcohol consumption [4]. These carcinogens induce chronic inflammation, promoting cancer progression through the release of pro-inflammatory cytokines (e.g., TNF, IL-6, IL-8, IL-1β), activation of transcription factors (e.g., NF-κB), and enhancement of angiogenesis [1]. The production of inflammatory cytokines, chemokines, and dysregulation of transcription factors contribute to proliferation, angiogenesis, and carcinogenesis in these tissues [1]. Induction of this inflammatory microenvironment, in turn, favors tumor progression. Nakano and collaborators (1999) identified elevated concentrations of pro-inflammatory cytokines TNF and IL-6 in tumor tissues of oral cavity cancer patients [5]. These cytokines can stimulate tumor cell progression through various mechanisms, playing a pathogenic role in cancer development. Subsequent studies have identified higher concentrations of TNF and interleukins (IL-6 and IL-8) in patients with oral cavity cancer [6,7]. The pro-inflammatory cytokine TNF was present in high concentrations in two oral cavity cancer cell models (HSC3 and SCC9), and this cytokine mediated nociception and inflammation induction in this type of cancer [8]. Finally, the pro-inflammatory cytokine TNF is also responsible for significantly promoting cancer progression by facilitating cellular invasion and metastasis [9]. Elevated levels of TNF have been observed in patients diagnosed with HNC and are associated with pain and inflammation. This indicates that approaches targeting the reduction of this cytokine may offer valuable contributions to treatment strategies [8].

HNC treatment is highly dependent on the disease stage and anatomical location. Standard therapies include surgery, radiotherapy, and chemotherapy, with cisplatin-based regimens being the mainstay [8]. However, treatment failure is common, with over 65% of patients experiencing recurrence or metastasis [10]. Although targeted therapies such as pembrolizumab (anti-PD-1) and cetuximab (anti-EGFR) have contributed to improved treatment outcomes, their effectiveness remains limited to a subset of patients, and resistance often emerges over time [11]. Small molecules represent an attractive alternative to biologics due to their lower cost, favorable pharmacokinetics, and oral bioavailability [12,13]. However, challenges such as limited selectivity and resistance development require the discovery of novel small-molecule candidates [12].

Chalcones are considered a privileged structure in medicinal chemistry due to their broad applications against various diseases and their ability to interact with multiple molecular targets [14,15]. This scaffold, characterized by a 1,3-diphenyl-2-propen-1-one nucleus in either the (*E*) or (*Z*) configuration, is recognized as a precursor to various flavonoid natural compounds [16,17]. Chemically, chalcones are α,β-unsaturated ketones with a conjugated double bond and a delocalized π–electron system spanning the benzene rings. They are typically low-molecular-weight molecules (<600 Da) with notable lipophilicity (Log P ≥ 5) [17]. It can exist in both cis (*Z*) and trans (*E*) isomeric forms, with the (*E*) configuration being thermodynamically more stable than the (*Z*) form [17].

Chalcones have demonstrated anticancer activity by targeting various molecular pathways involved in carcinogenesis, including epigenetic enzymes (such as histone deacetylases and sirtuins), proteasomes, NF-κB, kinases, cathepsin-K, and tubulin, among others [18]. However, this scaffold presents pharmacokinetic and oral bioavailability challenges due to its high lipophilicity. Despite these limitations, chalcones—both synthetic and natural—still continue to be extensively investigated, with structural modifications in phenyl rings A or B being explored to enhance selectivity for specific targets and improve physicochemical properties [19,20,21].

Building on the chalcone scaffold, the present study hypothesizes that incorporating a boronic acid moiety into the compound’s core structure could enhance its anticancer activity while modulating key physicochemical properties, such as solubility. Boron-containing compounds have gained attention in medicinal chemistry as pharmacophoric groups in several drugs such as crisaborole, bortezomib, and tavaborole, already approved for clinical use [22]. In a previous study conducted by our research group, we investigated the anticancer potential of boron-containing compounds by evaluating boronic acids substituted at the *meta* and *para* positions of the phenyl ring, as well as benzoboroxole derivatives, through a primary screening using the SCC-25 squamous cell carcinoma cell line model [23]. Two compounds demonstrated cytotoxic activity, with IC_50_ values of 59.07 µM and 45.61 µM, and corresponding selectivity indices (SI) of 3.73 and 3.37, respectively, while exhibiting no cytotoxic effects toward non-tumoral NOK-si cells (IC_50_ > 220.7 µM) [23]. Notably, none of the benzoboroxole derivatives exhibited activity against SCC-25 cells, and no discernible structure–activity relationship could be established [23]. These findings motivated the continued exploration of boron-containing compounds against HNC.

Previous studies [24,25,26,27] have investigated boronic chalcone derivatives across various cancer cell lines, highlighting the potential of this scaffold for the development of novel anticancer agents. Building upon these findings, the present study examines a new series of boronic chalcones as potential therapeutic candidates for HNC. Specifically, we assess their cytotoxicity and selectivity in HNC cell models and further explore their mechanisms of action, with particular emphasis on p53 dependency and cell death pathways. Given the anti-inflammatory activity reported for certain chalcones in the literature [28,29,30], we also evaluated whether the most cytotoxic compound could reduce levels of pro-inflammatory cytokines (TNF, IL-6, IL-1β, and IL-8), thereby assessing its potential role in modulating inflammation. Additionally, drug-like properties such as solubility, permeability, and metabolic stability were characterized to inform future efforts in structural optimization.

## 2. Results

### 2.1. Design and Synthesis of Boronic Chalcones Derivatives

**Figure 1:**
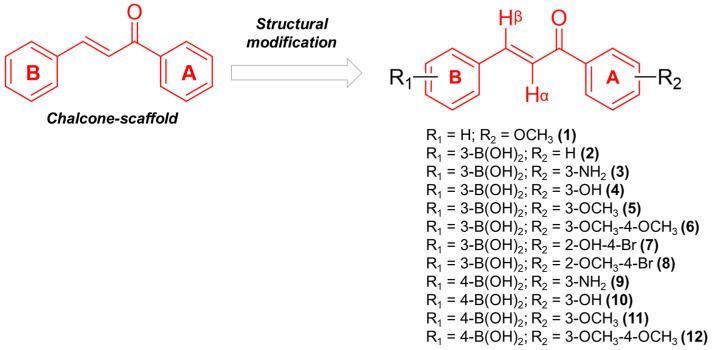
Structural modification of chalcone-core.

The chalcones were rationally designed to incorporate a boronic acid moiety into the *B-ring*, strategically positioned at either the *meta* or *para* position relative to the α,β-unsaturated carbonyl system, in accordance with the design strategy previously established by our group [23]. Furthermore, a variety of substituents were introduced into the *A-ring* to investigate their potential to enhance cytotoxic activity against cancer cells (Figure 1). Although prior studies have explored boronic acid substitution on both the *A-ring* and *B-ring*, findings by Kong et al. (2009) indicate that cytotoxic effects were more pronounced when the boronic acid was introduced into the *B-ring* [27]. To evaluate the significance of the boronic acid moiety for the molecule’s activity, compound **1** was designed without this functional group.

**Scheme 1:**
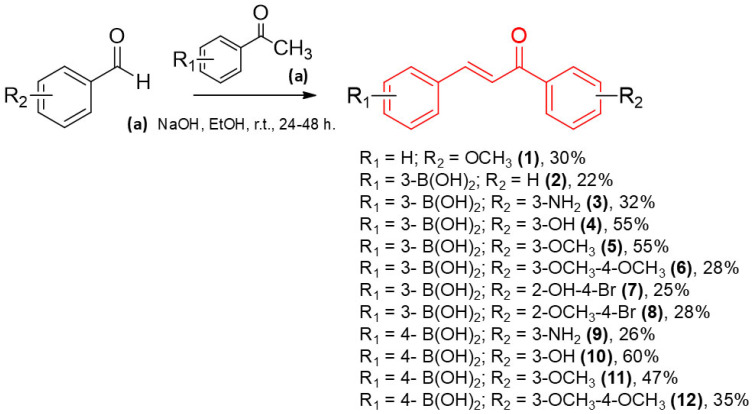
General route for the obtained compounds (**1**–**12**).

The synthetic route for the synthesis of compounds **1**–**12** are outlined in Scheme 1. Chalcone derivatives were synthesized via conventional Claisen–Schmidt condensation between the corresponding functionalized ketones and aldehydes, employing sodium hydroxide (NaOH) in ethanol (EtOH) as the reaction medium, and stirring for 24 to 48 h at ambient temperature. The compounds were obtained with yields ranging from 22% to 60%, and the (E) configuration was confirmed by nuclear magnetic resonance (NMR), as evidenced by the coupling constants of the vinylic system (*J*_Hα-Hβ_ = 15–16 Hz). The ^13^C NMR spectrum displayed a characteristic signal for the chalcone carbonyl group in the 188–190 ppm region. The presence of the boronic acid moiety was supported by a singlet in the 7.87–8.22 ppm range, integrating two protons. As expected, the ^13^C NMR spectrum did not reveal the quaternary carbon directly bonded to the boron atom, likely due to quadrupolar relaxation of the ^11^B nucleus, which commonly results in broadening or loss of the ^13^C signal for such carbons [31]. Nevertheless, 2D NMR experiments indicated long-range coupling between the hydroxyl protons of the boronic acid and a carbon signal, which we attribute to the carbon directly bonded to boron (Supplementary Materials). Additionally, all chalcone derivatives were analyzed by Liquid Chromatography–Mass Spectrometry (LC-MS) and high-performance liquid chromatography (HPLC) (Supplementary Materials).

### 2.2. Determination of Log P and In Vitro Cytotoxicity in Cancer Models

#### 2.2.1. Assessment of Log P and In Vitro Activity in SCC-25 and NOK-si Cells

All 12 compounds were evaluated for their biological activity against NOK-si (normal oral keratinocytes) and SCC-25 cells (a squamous cell carcinoma line derived from the human tongue). Only the most promising compounds were selected for evaluation against Detroit-562 cells (metastatic pharyngeal cancer model isolated from pleural fluid). The IC_50_ values and selectivity indices (SIs) were determined (Table 1). The physicochemical properties of the synthesized compounds were also assessed by determining their partition coefficient (Log P), enabling evaluation of their compliance with Lipinski’s Rule of Five, which predicts optimal drug-likeness for molecules with Log P values ≤ 5 [32] (Table 1).

**Figure 2:**
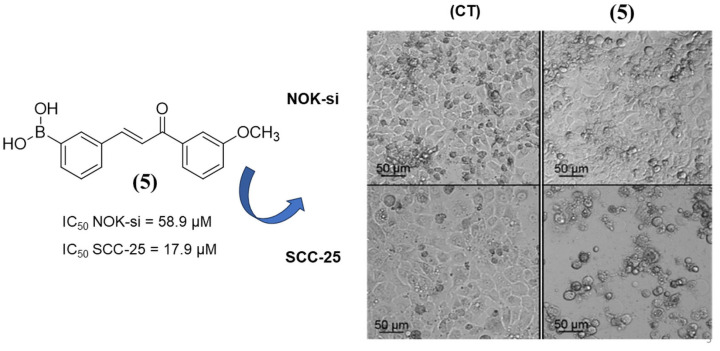
Microscopy image of chalcone **5** following 24 h of incubation with NOK-si and SCC-25 cell models at a concentration of 9.45 μM each.

Boronic chalcones **4** and **5** exhibited the most promising IC_50_ values against the SCC-25 cell line in the series. Compound **5** demonstrated a favorable balance between inhibitory activity against cancer cell lines and normal cells, with a selectivity index (SI) greater than 3. The microscopy image (Figure 2) showed that after 24 h of incubation with compound **5**, only the oral cavity cancer cells (SCC-25) exhibited morphological alterations suggesting cell death features (small rounding-shaped cells—Figure 2).

The drug 5-fluorouracil (5-FU) was used as an anticancer control, demonstrating an IC_50_ of 1800 μM for the SCC-25 cell model. In comparison, compound **5**, which has an IC_50_ of 17.9 μM for the SCC-25 cell model, proved to be superior to the reference drug. Furthermore, 5-FU lacked selectivity for the cancer cell model, as normal cells were statistically more sensitive than cancer cells (Supplementary Materials—Figure S1). Within this series, derivatives **4** and **5** were selected for evaluation against the Detroit-562 cell line, a metastatic pharyngeal cancer model isolated from pleural fluid. The IC_50_ values were subsequently determined (Table 1). Both compounds exhibited comparable IC_50_ values, although these were higher than those observed for the carcinoma cell line (SCC-25). Notably, compound **5** demonstrated an IC_50_ of 58.9 µM against the normal cell line (NOK-si) and an SI greater than 1.7 when assessed against the metastatic cell line. Moreover, all compounds exhibited Log P values below 5, aligning with the criteria defined by Lipinski’s Rule of Five.

#### 2.2.2. Cytotoxicity of Chalcones in p53^+/+^ and p53^−/−^ Cell Lines

Given that the cell lines used, SCC-25 and Detroit-562, are p53 mutated, it was intended to evaluate whether the molecules would also be effective in a wild-type p53-expressing cancer cell line. Furthermore, assessing whether the molecules exhibit enhanced cytotoxicity or selectivity in a specific model could provide valuable insights into their potential molecular targets. Accordingly, as presented in Table 1, seven chalcones were selected based on their promising anticancer activity profile for further evaluation in two additional colon cancer cell lines: HCT116 p53^+/+^ (with wild-type p53) and HCT116 p53^−/−^ (isogenic cells lacking p53) (Table 2). The results suggested that none of the evaluated compounds exhibited a significant difference in activity between the p53^+/+^ and p53^−/−^ cell lines, indicating that chalcones may exert their anticancer effects through a p53-independent pathway.

#### 2.2.3. Mechanism of Cell Death Triggered by Chalcone 5

**Figure 3:**
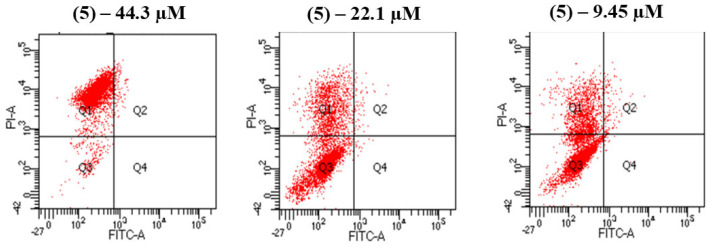
Annexin V-FITC/PI analysis of chalcone **5** carried out at three different concentrations: 44.3 μM, 22.1 μM, and 9.45 μM. Q1: Early necrotic cells; Q2: Late apoptotic cells; Q3: Viable cells; Q4: Early apoptotic cells.

Annexin V-FITC/PI analyses were conducted to examine the cell death process induced by compound **5**, identified as the most promising candidate in the series. At a concentration of 44.3 μM, the proportion of viable cells (Q2) was reduced to less than 5%. Across all tested concentrations—44.3 μM, 22.1 μM, and 9.45 μM—the percentage of necrotic cells (Q1) exceeded that of early apoptotic (Q4) and late apoptotic cells (Q3) (Figure 3). These findings suggest that compound **5** predominantly induces necrotic cell death in SCC-25 cells, as indicated by the higher prevalence of necrotic cells (Q1) compared to apoptotic populations (Q3, Q4) across all tested concentrations. Further analyses may provide additional confirmation of the necrotic nature of the cell death process.

#### 2.2.4. Chalcone 5 Drastically Reduces Levels of Pro-Inflammatory Cytokines: TNF, IL-6, IL-1β

**Figure 4:**
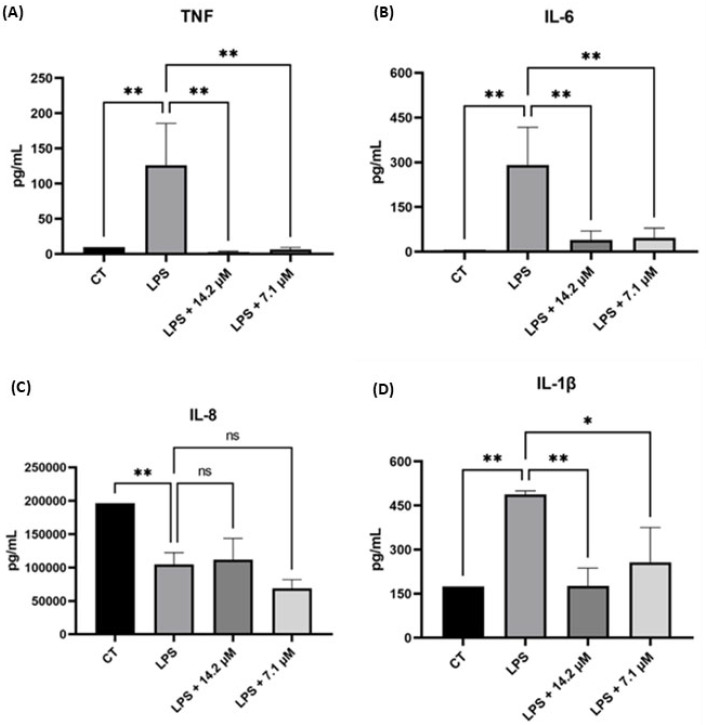
Evaluation of chalcone **5** in reducing levels of (**A**) TNF, (**B**) IL-6, (**C**) IL-8, and (**D**) IL-1β in LPS-stimulated THP-1 cells at two different concentrations. Control (CT) = THP-1 cells not stimulated with LPS. * *p* < 0.05, ** *p* < 0.01, ns = not significant.

Given the role of cytokines in the tumor microenvironment, the most active compounds against HNC cell lines were further evaluated for their potential to reduce pro-inflammatory cytokines while maintaining their anticancer activity. The two most potent and selective compounds—**1** and **5**—were chosen to investigate the anti-inflammatory effects. These compounds were tested at non-cytotoxic concentrations, and their effects on key inflammatory cytokines, including TNF, IL-6, IL-1β, and IL-8, were analyzed (Figure 4 and Supplementary Materials).

The findings indicate that chalcone derivatives effectively reduced the levels of TNF, IL-6, and IL-1β cytokines. Notably, chalcone **5** presented a pronounced reduction for TNF levels and for IL-6 beyond those observed in the control group (Figure 4). Compounds **1** and **5** differ structurally only by the presence of the boronic acid group; however, **1** demonstrated a lower effectiveness than that of the boronic chalcones (Supplementary Materials—Figure S2), suggesting that this moiety was crucial for achieving substantial TNF and IL-6 reduction. For IL-1β, the differences among the two compounds were less pronounced, showing comparable efficacy in reducing cytokine levels relative to LPS-stimulated cells (Figure 4 and Supplementary Materials—Figure S2). Finally, for IL-8, compound **5** effectively reduced cytokine levels compared to the control (Figure 4).

#### 2.2.5. Compound **5** Exhibits a Promising DMPK Profile

Compound **5** was selected for DMPK evaluation to identify the optimal balance between pharmacokinetic properties and anticancer/anti-inflammatory activity. The results are presented in Table 3.

Compound **5** presented a favorable pharmacokinetic profile, with a LogD value of 3.92, indication a balancing lipophilicity and aqueous solubility. This suggests a good potential for passive membrane diffusion and oral absorption. Also, the KS value of >39.65 indicates that this molecule presented moderate-to-high solubility. The permeability assessment through the PAMPA assay indicates high passive permeability. The half-life (T_1_/_2_) of compound **5** in HLM was 48.81 min, while in MsLM, it was 56.80 min. These values suggest a moderate metabolic stability. However, the intrinsic clearance (Clint) values in HLM (77.02 µL/min/mg) and MsLM (36.00 µL/min/mg) suggest a relatively higher clearance rate in humans compared to mice [33].

## 3. Discussion

In this present study, new boronic chalcones were evaluated as potential anticancer agents using the SCC-25 cell line. Both compounds **4** and **5** exhibited cytotoxicity, with compound **5** emerging as the most promising against the SCC-25 cell line (IC_50_ = 17.9 µM), given its selectivity index (SI) >3, indicating a favorable selectivity profile. The methoxy-to-hydroxyl substitution in compound **4** resulted in increased cytotoxic activity (IC_50_ = 9.8 µM) but a lower SI (1.88), suggesting a trade-off between potency and selectivity. In contrast, compound **1**, which lacks the boronic acid moiety, demonstrated the highest SI but a weaker IC_50_ (24.9 µM), reinforcing the importance of boronic acid for cytotoxic potency against the cancer cell line. Moreover, compound **11**, where the boronic acid group is positioned differently on the phenyl ring (in *para* position), exhibited an IC_50_ value three times higher than compound **5**, further emphasizing the structural relevance of boronic acid positioning.

The lipophilicity (Log P) of the synthesized chalcones was evaluated, yielding values ranging from 1.18 to 4.68, in agreement with Lipinski’s Rule of Five. Furthermore, the compounds exhibited fewer than five hydrogen bond donors, no more than 10 hydrogen bond acceptors, and molecular weights below 500 Da, thereby meeting all parameters outlined by Lipinski’s criteria. A comparison between compounds 1 and 5 revealed that the incorporation of the boronic acid moiety contributed to a reduction in lipophilicity (Table 1), a modification designed to enhance the physicochemical profile of the molecules, potentially improving solubility. Compound **5** was selected for further investigation, as it demonstrated the most favorable balance between anticancer activity, selectivity index, and lipophilicity. It was subsequently tested against the Detroit-562 cell line, a metastatic model of HNC. Although its inhibitory potency in this model (IC_50_ = 33.1 µM) was lower compared to that observed in the SCC-25 cell line, its reduced cytotoxicity toward the non-tumoral NOK-si cell line (IC_50_ = 58.9 µM) remained noteworthy, indicating a modest yet meaningful degree of selectivity for cancer cells.

To investigate potential targets, cytotoxicity was assessed in p53^+/+^ cells, revealing no significant difference between p53+ and p53− cell lines, suggesting that boronic chalcones operate through a p53-independent mechanism. Annexin V-FITC/PI assays suggest that compound **5** induces cell death primarily via necrosis. The potential of compound **5** to reduce pro-inflammatory cytokines was assessed for TNF, IL-6, IL-1β, and IL-8. Compound **5** exhibited the most pronounced effect on TNF levels, reducing them below those of untreated THP-1 cells at 7.1 μM—approximately half its IC_50_ value against SCC-25 cells. These findings suggest that compound **5** could exert both anticancer and anti-inflammatory effects. Since pro-inflammatory cytokines play a key role in cancer progression, their suppression could enhance the compound’s anticancer properties. Moreover, the role of boronic acid in anti-inflammatory activity was confirmed by the weaker cytokine reduction by compound **1**, which lacks this functional group.

Finally, the DMPK properties were assessed, showing that compound **5** demonstrated moderate solubility and high permeability, supporting its potential for oral bioavailability. However, its high clearance in human liver microsomes (HLMs) suggests that rapid hepatic metabolism could limit systemic exposure. These findings suggest that further structural modifications may be necessary to prolong the half-life of compound **5** and enhance its metabolic stability.

## 4. Materials and Methods

### 4.1. General Information

The reactions were monitored using thin-layer chromatography (TLC), precoated with silica gel 60 (HF-254; Sigma-Aldrich, St. Louis, MA, USA) to a thickness of 0.25 mm. The plates were revealed under UV light (254 and 365 nm). Column chromatography was conducted on Sigma-Aldrich^®^ 60 Å silica gel (40–60 μm, 230–400 mesh) (Sigma-Aldrich, St Louis, MO, USA), using ethyl acetate and n-hexane as eluents (mobile phase). Melting points were determined using Stuart Scientific^®^ Melting Point Apparatus SMP3 capillary equipment (Bibby Stuart Scientific, Cole-Parmer, Staffordshire, UK). FTIR analyses were performed using a NICOLET iS5 instrument equipped with the iD1 Transmission module (Thermo Scientific, Waltham, MA, USA). The scanning range was 4000–400 cm^−1^, with 32 scans for the background and 32 scans for the sample, at a resolution of 2 cm^−1^. Samples were diluted in spectroscopy-grade KBr (Merck, Darmstadt, Germany) and pressed into pellets using a pellet press for 1 min under a pressure of 6 tons. The purity for all compounds was characterized by HPLC using a Shimadzu LC-10AD chromatograph equipped with a model SPD-10A UV–VIS detector (Shimadzu, Kyoto, Japan), using MeOH/H_2_O (70:30) as the mobile phase. Nuclear magnetic resonance (NMR) spectra for ^1^H and ^13^C of all compounds were obtained using Bruker Avance III 600 (Bruker Corporation, Billerica, MA, USA) equipment operating at 600 and 150 MHz for ^1^H and ^13^C nuclei, respectively. Chemical shifts were expressed in parts per million (ppm) relative to tetramethylsilane. The coupling constants were reported in hertz (Hz), and the signal multiplicities were reported as singlet (s), doublet (d), doublet of doublet (dd), doublet of doublet of doublets (ddd), triplet (t), and multiplet (m). High-resolution mass spectrometry (ESI+) spectra were obtained by using the Bruker Maxis Impact quadrupole time-of-flight tandem mass spectrometer (Q-TOF MS/MS) (Bruker Corporation, Billerica, MA, USA). Solvents and reagents were purchased from the commercial suppliers Acros (Fukuoka, Japan), Alfa Aesar (Heysham, UK), Oakwood Chemical (Estill, SC, USA), Sigma-Aldrich (Saint Louis, MO, USA), and Synth (San Francisco, CA, USA).

### 4.2. General Procedure for the Synthesis of Chalcones Derivatives (**1**–**12**)

In a 10 mL round-bottom flask, a 1M NaOH solution in ethanol (96%) was prepared, followed by the addition of the respective functionalized ketones (1 eq.). Subsequently, the corresponding aldehydes (1 eq.) were dissolved in 4 mL of ethanol and added dropwise to the reaction mixture. The reaction was stirred at room temperature for 24 to 48 h. Reaction progress was monitored by TLC, using hexane and acetate as the mobile phase and UV light (254 nm) until consumption of the reagents indicated completion. At the end of the reaction, the pH of the reaction mixture was adjusted using a 10% hydrochloric acid (HCl) solution and poured into an ice bath. The formed precipitate was kept refrigerated overnight. The precipitate was then filtered using a Büchner funnel and washed with cold water. The resulting residues were purified by flash column chromatography (eluent n-hexane/AcOEt). Compounds were individually evaluated to determine the optimal mobile phase. After purification, products (**1**–**12**) were obtained, with yields ranging from 20% to 60%.

*(E)-3-(3-methoxyphenyl)-1-phenylprop-2-en-1-one* (**1**)

Compound **1** was purified by chromatography column with hexane/ethyl acetate (8:2 *v*/*v*) as eluents, leading to obtaining the pure product as a yellow oil with a 30% yield. ^1^H NMR (600 mHz, DMSO-*d*_6_): δ 7.93 (*d*, *J* = 15.7 Hz, 1H, Hα); 7.91–7.89 (*m*, 2H); 7.80–7.77 (*m*, 1H); 7.75 (*d*, *J* = 15.7 Hz, 1H, Hβ); 7.62 (*dd*, *J* = 2.7, 1.6 Hz, 1H); 7.49 (*t*, *J* = 7.9 Hz, 1H); 7.47–7.45 (*m*, 3H); 7.24 (*ddd*, *J* = 8.2, 2.7, 0.9 Hz, 1H); 3.85 (*s*, 3H, OCH_3_). ^13^C (150 mHz, DMSO-*d*_6_): 188.95; 159.58; 144.14; 139.02; 134.65; 130.68; 129.96; 128.98 (2C); 128.93 (2C); 122.10; 121.09; 119.25; 113.00; 55.39. HMRS (ESI^+^, methanol) m/z calculated for C_16_H_14_O_2_: 238.10, found: [M + H]^+^ = 239.1064, Rt = 4.53 min. FTIR (KBr) *ν*max 3457 (residual H_2_O); 3060–2837 (C-H aromatic or aliphatic); 1665 (C=O α,β-unsaturated ketone); 1591 (C=C aromatic); 1251 (C-O methoxyl); 1331 (C-O methoxyl); 1033 (C-O methoxyl).

*(E)-(3-(3-oxo-3-phenylprop-1-en-1-yl)phenyl)boronic acid* (**2**)

Compound **2** was purified by chromatography column with hexane/ethyl acetate (8:2 *v*/*v*) as eluents, leading to obtaining the pure product as a white solid with a 22% yield; mp: 90–92 °C; ^1^H NMR (600 mHz, DMSO-*d*_6_): δ 8.23 (*s*, 1H, Ar-H); 8.22 (*s*, 2H, B-OH boronic acid); 8.13 (*dd*, *J* = 8.4; 1.3 Hz, 1H); 7.93–7.91 (*m*, 1H); 7.89 (*d*, *J* = 15.6 Hz, 1H, Hα); 7.85 (*dt*, *J* = 7.4, 1.1 Hz, 1H); 7.74 (*d*, *J* = 15.6 Hz, 1H, Hβ); 7.70–7.66 (*m*, 1H); 7.63–7.55 (*m*, 2H); 7.44 (*t*, *J* = 7.5 Hz, 1H). ^13^C (150 MHz, DMSO-*d*_6_): 188.36; 144.47; 137.69; 136.53; 134.53; 133.68; 133.25; 130.64; 128.94 (2C); 128.51 (2C); 128.24; 121.77. HMRS (ESI^+^, methanol) *m*/*z* calculated for C_15_H_13_BO_3:_ 252.09, found: [M + H]^+^ = 253.1036, Rt = 6.67 min. FTIR (KBr) *ν*max 3415 (B-OH boronic acid); 1650 (C=O α,β-unsaturated ketone); 1587 (C=C aromatic; 1316 (B–O boronic acid).

*(E)-(3-(3-(3-aminophenyl)-3-oxoprop-1-en-1-yl)phenyl)boronic acid* (**3**)

Compound **3** was purified by chromatography column with hexane/ethyl acetate 4:6 as eluents, leading to obtaining the pure product as a white solid with a 32% yield; mp: 113–117 °C; ^1^H NMR (600 MHz, DMSO-*d*_6_): δ 8.22 (*s*, 1H, Ar-H), 8.20 (*s*, 2H, B-OH boronic acid), 7.87–7.83 (*m*, 2H), 7.75 (*d*, *J* = 15.7 Hz, 1H, Hα), 7.68 (*d*, *J* = 15.6 Hz, 1H, Hβ), 7.43 (*t*, *J* = 7.5 Hz, 1H), 7.29 (*dt*, *J* = 7.6, 1.2 Hz, 1H), 7.26–7.24 (*m*, 1H), 7.21 (*t*, *J* = 7.8 Hz, 1H), 6.84 (*ddd*, *J* = 7.9, 2.4, 1.0 Hz, 1H), 5.38 (s, 2H, NH_2_). ^13^C (150 MHz, DMSO-*d*_6_): 189.60; 149.18; 143.74; 138.49; 136.27; 134.77; 134.03; 133.70; 130.59; 129.21; 128.13; 122.04; 118.56; 116.16; 112.91. HMRS (ESI^+^, methanol) m/z calculated for C_15_H_14_BNO_3:_ 267.10, found: [M + H]^+^ = 268.1141, Rt = 3.32. FTIR (KBr) *ν*max 3337 (N-H); 3320 (B-OH boronic acid); 1656 (C=O α,β-unsaturated ketone); 1588 (C=C aromatic); 1289 (B–O boronic acid).

*(E)-(3-(3-(3-hydroxyphenyl)-3-oxoprop-1-en-1-yl)phenyl)boronic acid* (**4**)

Compound **4** was purified by chromatography column with hexane/ethyl acetate (3:7 *v*/*v*) as eluents, leading to obtaining the pure product as a pale-yellow solid with a 55% yield; mp: 168–172 °C; ^1^H NMR (600 MHz, DMSO-*d*_6_): δ 9.82 (*s,* 1H, OH), 8.23 (*s*, 1H, Ar-H), 8.20 (*s*, 2H, B-OH boronic acid), 7.89 (*d, J* = 7.8, 1.6 Hz, 1H), 7.86–7.84 (*m*, 1H), 7.81 (*d*, *J* = 15.6 Hz, 1H, Hα), 7.71 (*d*, *J* = 15.6 Hz, 1H, Hβ), 7.61–7.58 (*m*, 1H), 7.46–7.43 (*m*, 3H), 7.38 (*t*, *J* = 7.9 Hz, 1H), 7.08–7.05 (*m*, 1H). ^13^C (150 MHz, DMSO-*d*_6_): 189.11; 157.75; 144.25; 139.07; 136.39; 133.62; 130.64; 129.90; 128.14; 121.82; 120.28; 119.44; 114.57. HMRS (ESI^+^, methanol) m/z calculated for C_15_H_13_BNO_4_: 268.09, found: [M + H]^+^ = 269.0983, Rt = 4.05. FTIR (KBr) *ν*max 3344 (B-OH boronic acid or O-H); 1653 (C=O α,β-unsaturated ketone); 1582 (C=C aromatic); 1358 (B–O boronic acid).

*(E)-(3-(3-(3-methoxyphenyl)-3-oxoprop-1-en-1-yl)phenyl)boronic acid* (**5**)

Compound **5** was purified by chromatography column with hexane/ethyl acetate (4:6 *v*/*v*) as eluents, leading to obtaining the pure product as a pale-yellow solid with a 55% yield; mp: 122–127 °C; ^1^H NMR (600 MHz, DMSO-*d*_6_): δ 8.23 (*s*, 1H, Ar-H), 8.20 (*s*, 2H, B-OH boronic acid), 7.94 (*d*, *J* = 7.7 Hz, 1H), 7.88 (*d*, *J* = 15.7 Hz, 1H, Hα), 7.85–7.84 (*m*, 1H), 7.76–7.71 (*m*, 2H), 7.59 (*dd*, *J* = 2.7, 1.6 Hz, 1H), 7.51 (*t*, *J* = 7.9 Hz, 1H), 7.44 (*t*, *J* = 7.5 Hz, 1H), 7.25 (*dd*, *J* = 8.0, 2.4 Hz, 1H), 3.86 (*s*, 3H, OCH_3_). ^13^C (150 MHz, DMSO-*d*_6_): 188.97; 159.59; 144.57; 139.09; 136.47; 134.58; 133.60; 130.56; 130.01; 128.13; 121.75; 120.98; 119.14; 112.99; 55.40. HMRS (ESI^+^, methanol) m/z calculated for C_16_H_15_BO_4_: 282.10, found: [M + H]^+^ = 283.1138, Rt = 7.86. FTIR (KBr) *ν*max 3400 (B-OH boronic acid); 1656 (C=O α,β-unsaturated ketone); 1585 (C=C aromatic); 1343 (B–O boronic acid); 1027 (C-O methoxyl).

*(E)-(3-(3-(3,4-dimethoxyphenyl)-3-oxoprop-1-en-1-yl)phenyl)boronic acid* (**6**)

Compound **6** was purified by chromatography column with hexane/ethyl acetate (3:7 *v*/*v*) as eluents, leading to obtaining the pure product as a pale-yellow solid with a 28% yield; mp: 79–81 °C; ^1^H NMR (600 MHz, DMSO-*d*_6_): δ 8.21 (*s*, 1H, Ar-H), 8.19 (*s*, 2H, B-OH boronic acid), 7.95–7.92 (*m*, 1H), 7.91 (*d*, *J* = 15.5 Hz, 1H, Hα), 7.88 (*d*, *J* = 2.0 Hz, 1H), 7.85 (*d*, *J* = 7.3 Hz, 1H), 7.71 (*d*, *J* = 15.5 Hz, 1H, Hβ), 7.60 (*d*, *J* = 2.1 Hz, 1H), 7.44 (*t*, *J* = 7.5 Hz, 1H), 7.12 (*d*, *J* = 8.5 Hz, 1H), 3.88 (*s*, 3H, OCH_3_), 3.86 (*s*, 3H, OCH_3_). ^13^C (150 MHz, DMSO-*d*_6_): 187.38; 153.25; 148.87; 143.51; 136.21; 134.57; 133.78; 130.55; 130.29; 128.08; 123.30; 121.60; 110.90; 110.71; 55.81; 55.61. HMRS (ESI^+^, methanol) *m*/*z* calculated for C_17_H_17_BO_5_: 312.11, found: [M + H]^+^ = 313.1242, Rt = 5.08. FTIR (KBr) *ν*max 3403 (B-OH boronic acid); 1650 (C=O α,β-unsaturated ketone); 1573 (C=C aromatic); 1266 (B–O boronic acid); 1167 (C-O methoxyl); 1033 (C-O methoxyl).

*(E)-(3-(3-(2-bromo-4-hydroxyphenyl)-3-oxoprop-1-en-1-yl)phenyl)boronic acid* (**7**)

Compound **7** was purified by chromatography column with hexane/ethyl acetate (3:7 *v*/*v*) as eluents, leading to obtaining the pure product as a yellow solid with a 25% yield; mp: 129–132 °C; ^1^H NMR (600 MHz, DMSO-*d*_6_): δ 12.44 (*s*, 1H, OH), 8.22 (*s*, 1H, Ar-H), 8.21 (*s*, 2H, B-OH boronic acid), 8.07 (*d*, *J* = 8.6 Hz, 1H), 7.94–7.89 (*m*, 2H), 7.87 (*dt*, *J* = 7.4, 1.2 Hz, 1H), 7.81 (*d*, *J* = 15.5 Hz, 1H, Hα), 7.45 (*t*, *J* = 7.5 Hz, 1H), 7.26 (*d*, *J* = 1.9 Hz, 1H), 7.20 (*dd*, *J* = 8.5, 1.9 Hz, 1H). ^13^C (150 MHz, DMSO-*d*_6_): 192.70; 161.66; 145.37; 136.82; 134.82; 133.39; 132.27; 130.77; 128.92; 128.19; 122.37; 121.98; 120.93; 120.36. HMRS (ESI^+^, methanol) m/z calculated for C_15_H_12_BBrO_4:_ 346.00, found: [M + H]^+^ = 347.0081, Rt = 4.29. FTIR (KBr) *ν*max 3409 (B-OH boronic acid or O-H); 1659 (C=O α,β-unsaturated ketone); 1594 (C=C aromatic); 1343 (B–O boronic acid); 1024 (C-O hydroxyl).

*(E)-(3-(3-(2-bromo-4-methoxyphenyl)-3-oxoprop-1-en-1-yl)phenyl)boronic acid* (**8**)

Compound **8** was purified by chromatography column with hexane/ethyl acetate (5:5 *v*/*v*) as eluents, leading to obtaining the pure product as a white solid with a 28% yield; mp: 110–113 °C; ^1^H NMR (600 MHz, DMSO-*d*_6_): δ 8.18 (*s*, 2H, B-OH boronic acid), 8.10 (*s*, 1H, Ar-H), 7.83 (*dt*, *J* = 7.4, 1.0 Hz, 1H), 7.78–7.75 (*m*, 1H), 7.48 (*d*, *J* = 16.0 Hz, 1H), 7.45–7.39 (*m*, 3H), 7.34 (*d*, *J* = 16.0 Hz, 1H, Hα), 7.28 (*dd*, *J* = 8.1, 1.8 Hz, 1H), 3.89 (*s*, 3H, OCH_3_). ^13^C (150 MHz, DMSO-*d*_6_): 191.61; 158.26; 144.01; 136.46; 134.32; 133.43; 131.00; 130.28; 128.20; 128.02; 126.38; 125.84; 123.60; 115.64; 56.44. HMRS (ESI^+^, methanol) *m*/*z* calculated for C_16_H_14_BBrO_4_: 360.01, found: [M + H]^+^ = 361.0168, Rt = 33.17. FTIR (KBr) *ν*max 3332 (B-OH boronic acid); 1635 (C=O α,β-unsaturated ketone); 1573 (C=C aromatic); 1361 (B–O boronic acid); 1203 (C-O methoxyl)**;** 1024 (C-O methoxyl).

*(E)-(4-(3-(3-aminophenyl)-3-oxoprop-1-en-1-yl)phenyl)boronic acid* (**9**)

Compound **9** was purified by chromatography column with hexane/ethyl acetate (4:6 *v*/*v*) as eluents, leading to obtaining the pure product as a white solid with a 26% yield; mp: 135–138 °C; ^1^H NMR (600 MHz, DMSO-*d*_6_): δ 7.91 (s, 2H, B-OH boronic acid), 7.63 (d, *J* = 7.9 Hz, 2H), 7.25 (d, *J* = 7.9 Hz, 2H), 7.15–7.06 (m, 5H), 6.77 (dt, *J* = 7.1, 2.1 Hz, 1H), 5.30 (s, 2H). ^13^C (150 MHz, DMSO-*d*_6_): 190.46; 149.34; 143.94; 138.75; 136.48; 135.04 (2C); 129.83; 128.13 (2C); 123.14; 119.37; 117.04; 113.53. HMRS (ESI^+^, methanol) *m*/*z* calculated for C_15_H_14_BNO_3_: 267.10, found: [M + H]^+^ = 268.1144, Rt = 3.00. FTIR (KBr) *ν*max 3415 (N-H); 3323 (B-OH boronic acid); 1665 (C=O α,β-unsaturated ketone); 1567 (C=C aromatic); 1334 (B–O boronic acid).

*(E)-(4-(3-(3-hydroxyphenyl)-3-oxoprop-1-en-1-yl)phenyl)boronic acid* (**10**)

Compound **10** was purified by chromatography column with hexane/ethyl acetate (3:7 *v*/*v*) as eluents, leading to obtaining the pure product as a pale-yellow solid with a 60% yield; mp: 162–164 °C; ^1^H NMR (600 MHz, DMSO-*d*_6_): δ 9.81 (*s*, 1H, OH), 8.19 (*s*, 2H, B-OH boronic acid), 7.93–7.81 (*m*, 5H), 7.70 (*d*, *J* = 15.7 Hz, 1H, Hα), 7.64 (*d*, *J* = 7.7 Hz, 1H), 7.48–7.45 (*m*, 2H), 7.38 (*t*, *J* = 7.9 Hz, 1H), 7.06 (*dd*, *J* = 7.8, 2.1 Hz, 2H). ^13^C (150 MHz, DMSO-*d*_6_): 189.25; 157.79; 143.90; 139.05; 136.09; 134.64 (2C); 129.97; 127.85 (2C); 122.61; 120.43; 119.70; 114.67. HMRS (ESI^+^, methanol) *m*/*z* calculated for C_15_H_13_BO_4_: 268.09, found: [M + H]^+^ = 269.0982, Rt = 3.82. FTIR (KBr) *ν*max 3370 (B-OH boronic acid or O-H); 1650 (C=O α,β-unsaturated ketone); 1582 (C=C aromatic); 1349 (B–O boronic acid).

*(E)-(4-(3-(3-methoxyphenyl)-3-oxoprop-1-en-1-yl)phenyl)boronic acid* (**11**)

Compound **11** was purified by chromatography column with hexane/ethyl acetate (4:6 *v*/*v*) as eluents, leading to obtaining the pure product as a pale-yellow solid with a 47% yield; mp: 134–139 °C; ^1^H NMR (600 MHz, DMSO-*d*_6_): δ 7.98 (*d*, *J* = 7.6 Hz, 2H), 7.95 (*d*, *J* = 15.7 Hz, 1H, Hα), 7.87 (*s*, 1H), 7.87–7.85 (*m,* 4H) 7.78 (*d*, *J* = 7.6 Hz, 1H), 7.74 (*d*, *J* = 15.6 Hz, 1H, Hβ), 7.62 (*s*, 1H), 7.51–7.48 (*m*, 1H), 7.25 (*dd*, J = 8.2, 2.3 Hz, 1H), 3.85 (*s*, 3H, OCH_3_). ^13^C (150 MHz, DMSO-*d*_6_): 189.00; 159.61; 144.17; 139.05; 137.19; 136.05; 134.61 (2C); 129.98; 128.34; 127.93; 122.45; 121.12; 119.27; 113.05; 55.41. HMRS (ESI^+^, methanol) *m*/*z* calculated for C_16_H_15_BO_4_: 282.10, found [M + H]^+^ = 283.1136, Rt = 7.43. FTIR (KBr) *ν*max 3376 (B-OH boronic acid); 1653 (C=O α,β-unsaturated ketone); 1585 (C=C aromatic); 1325 (B–O boronic acid); 1027 (C-O methoxyl).

*(E)-(4-(3-(3,4-dimethoxyphenyl)-3-oxoprop-1-en-1-yl)phenyl)boronic acid* (**12**)

Compound **12** was purified by chromatography column with hexane/ethyl acetate (3:7 *v*/*v*) as eluents, leading to obtaining the pure product as a pale-yellow solid with a 35% yield; mp: 120–122 °C; ^1^H NMR (600 MHz, DMSO-*d*_6_): δ 8.17 (*s*, 2H, B-OH boronic acid), 7.99 (*d*, *J* = 15.6 Hz, 1H, Hα), 7.92 (*dd*, *J* = 8.5, 2.0 Hz, 1H), 7.88–7.82 (*m*, 4H), 7.71 (d, *J* = 15.5 Hz, 1H, Hβ), 7.62 (*d*, *J* = 2.0 Hz, 1H), 7.11 (*d*, *J* = 8.5 Hz, 1H), 3.88 (*s*, 3H, OCH_3_), 3.87 (*s*, 3H, OCH_3_). ^13^C (150 MHz, DMSO-*d*_6_): 187.35; 153.28; 148.82; 143.09; 136.19; 134.53 (2C); 130.48; 127.76 (2C); 123.45; 122.27; 110.91; 110.76; 55.80; 55.62. HMRS (ESI^+^, methanol) *m*/*z* calculated for C_17_H_17_BO_5_: 312.11, found [M + H]^+^ = 313.1245, Rt = 4.67. FTIR (KBr) *ν*max 3439 (B-OH boronic acid); 1650 (C=O α,β-unsaturated ketone); 1570 (C=C aromatic); 1346 (B–O boronic acid); 1149 (C-O methoxyl); 1006 (C-O methoxyl).

### 4.3. Determination of Partition Coefficient (Log P)

The determination of the partition coefficient was conducted in accordance with the OECD Guidelines for the Testing of Chemicals, Test No. 117: Partition Coefficient (n-octanol/water), HPLC Method [34]. The assay was performed using a Shimadzu^®^ high-performance liquid chromatography (HPLC) system equipped with a UV–VIS detector. Analyses were carried out under isocratic conditions, using a mobile phase composed of methanol and water (70:30, *v*/*v*) at a flow rate of 1.0 mL/min. The injection volume was set at 20 μL, and detection was performed at a wavelength of 210 nm. A C18 column (18 cm) was employed for the separation. To construct the calibration curve of log K versus log P, the following reference compounds were used: aniline (log P 0.9), acetanilide (log P 1.0), chlorobenzene (log P 2.8), naphthalene (log P 3.6), biphenyl (log P 4.0), triphenylamine (log P 5.7), and DDT (log P 6.5). The retention factor (K) was calculated using the expression (Tr − T_0_)/T_0_, where Tr corresponds to the retention time of the analyte, and T_0_ represents the dead time, defined as the average time taken by the mobile phase to elute through the chromatographic column. All analyses were performed in triplicate to ensure reproducibility.

### 4.4. Biological Evaluation

#### 4.4.1. Cell Lines

The SCC-25 (ATCC^®^ CRL-1628™), Detroit-562 (ATCC^®^ CCL-138™), and THP-1 (ATCC^®^ TIB-202™) cells were purchased from Rio de Janeiro Cell Bank (Duque de Caxias, Brazil), and NOK-si was kindly provided by Prof. Carlos Rossa from the School of Dentistry—UNESP. All the cells were cultivated according to standard conditions [35,36]. Human colorectal adenocarcinoma HCT116 cell lines (ATCC^®^ CCL-247™) expressing p53 and its p53-null isogenic derivative (HCT116 p53^−/−^) were provided by B. Vogelstein (The Johns Hopkins Kimmel Cancer Center, Baltimore, MD, USA).

#### 4.4.2. Cytotoxicity Assay on HNC Cell Lines (MTT Protocol)

Cells (NOK-si, SCC-25, and Detroit-562) were grown in a DMEM medium (Dulbecco’s Modified Eagle Medium), supplemented according to the needs of each lineage, as recommended by its cell banks, with 2 mM L-glutamine, 10% or 20% fetal bovine serum (FBS), 1% antibiotic–antimycotic solution (5 mg/mL penicillin, 5 mg/mL streptomycin, and 10 mg/mL neomycin), 4.5 g/L of glucose, and 3.7 g/L of sodium bicarbonate (NaHCO_3_). All cell lines were cultured in flasks at 37 °C in 5% carbon dioxide (CO_2_) and 95% of relative humidity. The assays were conducted between the third- and the seventh-cell passage, with sub-cultures every 3–4 days to maintain exponential growth. The cell viability was checked using the trypan blue dye exclusion assay for all experiments, where over 95% of the cells were viable at the beginning of the tests. NOK-si, SCC-25, and Detroit-562 were initially thawed and cultured in 25 cm^2^ cell culture flasks. Cell growth was monitored using a microscope, and the culture medium was changed periodically. Upon reaching confluency, cell dissociation was achieved by adding 5 mL of trypsin (Sigma-Aldrich, St Louis, MO, USA) and incubating the bottles in a CO_2_ incubator for 10 min. Subsequently, the cells were transferred to 15 mL Falcon tubes and centrifuged at 2000 rpm for 10 min. Cell counting was performed using a Countess II FL automated cell counter (ThermoFischer, Waltham, MA, USA). The cells were seeded at a density of 30,000 cells per well in 96-well plates. The plates were then incubated at 37 °C in 5% CO_2_ for 24 h. Following this, test compounds were incubated for another 24 h at 10 different concentrations: 50, 25, 12.5, 6.25, 3.12, 1.56, 0.78, 0.39, 0.19, and 0.097 µg/mL. After the specified time, MTT reagent (3 mg/mL) (Sigma-Aldrich, St Louis, MO, USA) dissolved in phenol-free RPMI 1640 was added and incubated for 4 h. The formed MTT crystals were solubilized using 2-propanol P.A. (Sigma-Aldrich, St Louis, MO, USA) with agitation. Absorbance was read at 562 nm using an EZ Read 400 microplate reader (Biochrom, Cambridge, UK) and ADAP 2.0 Biochrom software (version 2.0). IC_50_ values and graphs were calculated using Excel and Prism software (version 10.4.1). The MTT assay results were evaluated for normality using the Shapiro–Wilk test, followed by one-way ANOVA with Tukey’s post hoc test at a significance level of 5%.

#### 4.4.3. Cytotoxicity Assay on HCT p53^+/+^ and HCT p53^−/−^ Cell Lines

HCT p53^+/+^ and HCT p53^−/−^ cell lines were seeded in 96-well plates, with a concentration of 5000 cells/well, and allowed to adhere overnight. Afterwards, they were treated with serial dilutions of the compounds for an additional 48 h (compounds were prepared at 30 mM, in DMSO, and tested with a starting concentration of 50μM and 15 μM). Cells passed then through a series of essential steps (fixation with 10% trichloroacetic acid, for 1 h at 4 °C; staining with 0.4% sulforhodamine B (SRB); wash with 1% acetic acid; and solubilization with 10 mM Tris-Base); subsequently, absorbance was measured at 510 nm in a microplate reader (Biotek Instruments Inc., Synergy, MX, USA). IC_50_ values were determined using GraphPad Prism software, Version 7.0 (La Jolla, CA, USA).

#### 4.4.4. Apoptosis/Necrosis Assay

For the assessment of the type of cell death, the treatment groups were 44.3 μM, 22.1 μM, and 9.45 μM compound **5**, and the negative control without any treatment (only DMEM medium). Then, after 24 h of treatment, cell suspensions of 1 × 10^6^ mL**^–^**^1^ (SCC-25) of each experimental condition were labeled, and the assay was performed according to the manufacturer’s protocol with the FITC Annexin V/Dead Cell Apoptosis Kit (Life Technologies, Eugene, OR, USA). Annexin V-FITC conjugated (510 nm) and propidium iodide (PI, 610 nm) fluorescence intensity were detected by automatic reading with the Countess™ II FL Automated Cell Counter (Life Technologies, Carlsbad, CA, USA). The cell population was calculated as a percentage of apoptotic cells labeled only with annexin V-FITC conjugated (green), necrotic cells labeled only as PI (red), and late apoptotic/necrotic cells (green and red).

#### 4.4.5. Anti-Inflammatory Assay

For this assay, THP-1 (BCRJ cód. 0234) cells were cultured in Roswell Park Memorial Institute (RPMI-1640, Sigma-Aldrich; Saint Louis, MO, USA) media supplemented with 2 mM L-glutamine, 1 mM sodium pyruvate, 1.50 g/L of sodium bicarbonate, and 10% fetal bovine serum. For obtaining Macrophage-like cells, 250,000 cells per well were plated in a 24-well cell culture plate. A total of 20 ng/mL of Forbol 12-miristato-13-acetato (PMA) was added to each well, and the cells were incubated for 48 h at 37 °C with 5% CO_2_. After the 48 h, the wells were washed with 1X PBS, and fresh RPMI-1640 media was added. The cells were then incubated for 24 h at 37 °C and 5% CO_2_. For the cell’s stimulations, after the 24 h, the well was washed with PBS and fresh RPMI1640 media without fetal bovine serum added. For the positive controls of the assay, 3 wells were stimulated with LPS (1 ng/µL) diluted in the same volume and media used for the drugs stimulation [37]. Two different concentrations (2 and 4 µg/mL) were evaluated for each molecule, which were determined to be non-cytotoxic through a cell viability assay. After a 24 h period, the supernatant was removed and used in the cytokine quantification assay. A kit for inflammatory cytokines, BD^®^ Cytometric Bead Array CBA Human Inflammatory, was employed for this assay, containing the following human cytokines: IL-8, IL-1β, IL-6, and TNF. Standard cytokines were prepared in 9 different concentrations: 1:256, 1:128, 1:64, 1:32, 1:16, 1:8, 1:4, 1:2, and 1:1 (“top standard”). Analysis was performed using a Flow Cytometer FACS Aria Fusion BD (Bioscence, Houston, TX, USA). Data analysis was conducted using Prism software (version 10.4.1).

### 4.5. DMPK Assays

#### 4.5.1. Equipment

DMPK analyses were performed using LC-MS/MS (liquid chromatography–tandem mass spectrometry). The chromatography system used was Prominence UFLC (Shimadzu Corporation, Kyoto, Japan) and the interface with an LCMS-8045 triple quadrupole mass spectrometer (Shimadzu Corporation, Kyoto, Japan) with an electrospray ionization source (ESI).

#### 4.5.2. Determination of Kinetic Solubility

To determine kinetic solubility, stock solutions of test compounds and controls [Amiodarone hydrochloride (Sigma-Aldrich, St. Louis, MO, USA), Diclofenac (Oakwood Chemical, Estill, SC, USA), Chloramphenicol (Sigma-Aldrich, St. Louis, MO, USA), and Itraconazole (Sigma-Aldrich, St. Louis, MO, USA)] were prepared at 10 mM in DMSO. A volume of 5 µL from each stock solution was transferred to designated wells of a 96-well incubation plate in duplicate. Subsequently, 195 µL of phosphate-buffered saline (PBS) at pH 7.4 or 2.0 was added to achieve a final compound concentration of 250 µM (final DMSO concentration ≤ 2.5%). The plate was sealed and subjected to shaking at 200 rpm for 24 ± 1 h at room temperature (25 °C). Following the incubation period, the plate was centrifuged at 3000 rpm for 15 min at 25 °C to remove precipitates. A 100 µL aliquot of the supernatant from each well was transferred to a corresponding well in a new 96-well plate. The samples were subsequently analyzed by an LCMS-8045 system (Shimazu, Kyoto, Japan). To quantify the solubilized fraction, a calibration curve was prepared for each test compound and control. From the 10 mM stock solution, an intermediate standard solution (0.5 mM) was prepared in a 1:1 acetonitrile:water mixture. Serial dilutions of this intermediate solution were performed to obtain final concentrations of 50, 40, 20, 2, and 1 µM. The equation derived from the calibration curve (y = mx + b) was used to determine the experimental concentrations. The chromatographic analysis was conducted using an LCMS-8045 system (Shimazu, Kyoto, Japan) equipped with a Supelco Ascentis Express C18 column (3 cm × 2.1 mm, 5 µm). The mobile phase consisted of (A) water + 0.05% formic acid and (B) acetonitrile + 0.05% formic acid. The elution was carried out in binary gradient mode with the following program: 0 min, 98% A; 1.2 min, 2% A; 2.0 min, 2% A; re-equilibration time: 0.6 min, 98% A. The total run time was 2 min, with a flow rate of 0.6 mL/min and an injection volume of 5 µL.

#### 4.5.3. Parallel Artificial Membrane Permeability Assays (PAMPA)

The parallel artificial membrane permeability assay (PAMPA) was performed using a 96-well pre-coated PAMPA plate system (Corning, Bedford, MA, USA). Solutions of test compounds and controls [metoprolol (Sigma-Aldrich, St. Louis, MO, USA) and nadolol (Sigma-Aldrich, St. Louis, MO, USA)] were prepared by diluting 10 mM stock solutions in DMSO with phosphate-buffered saline (PBS) at pH 6.5 to obtain a final concentration of 10 μM (final DMSO concentration ≤ 1%). The donor compartment was loaded with 300 μL of the prepared compound solutions, while the acceptor compartment received 200 μL of PBS pH 7.4. The plate was then assembled, and the system was incubated at 37 °C, with shaking at 100 rpm for 5 h. Samples of the initial donor solution (T0) were collected (10 μL) and transferred to an analysis plate. A quenching solution [10% water and 90% methanol: acetonitrile (50:50) + 50 nM tolbutamide (Sigma-Aldrich, St. Louis, MO, USA) as internal standard] was added (300 μL), along with 60 μL of PBS buffer pH 6.5. The analysis plate was stored at −20 °C. At the end of the incubation period, samples were collected from the donor and acceptor compartments and added to the analysis plate, following the same quenching procedure. The final concentrations of the compounds in the donor, acceptor, and T0 wells were quantified by an LCMS-8045 system (Shimazu, Kyoto, Japan). Chromatographic analysis was performed using a Supelco Ascentis Express C18 column (3 cm × 2.1 mm, 5 μm). The mobile phase consisted of (A) water + 0.1% formic acid and (B) acetonitrile + 0.1% formic acid. The elution was carried out in binary gradient mode with the following program: 0 min, 95% A; 0.05 min, 95% A; 0.3 min, 2% A; 0.7 min, 2% A; 0.8 min, 95% A; 1.15 min, 95% A; 2.0 min, 95% A. The total run time was 2 min, with a flow rate of 0.7 mL/min and an injection volume of 10 μL.

#### 4.5.4. Determination of Distribution Coefficient (eLogD)

The determination of the experimental distribution coefficient (eLogD) was based on the retention time of molecules in the stationary phase using high-performance liquid chromatography (HPLC). Chromatographic analysis was performed on a Supelco Ascentis Express RP Amide HPLC column (5 cm × 2.1 mm, 2.7 μm), with a binary gradient mobile phase consisting of (A) 5% methanol in 10 mM ammonium acetate pH 7.4 and (B) 100% methanol. The mobile phase was eluted as follows: 0 min, 95% A; 0.3 min, 100% A; 5.2 min, 0% A; 5.6 min, 0% A; 5.8 min, 100% A; 7.0 min, 100% A. The total run time was 7 min, with a sample injection volume of 5 μL. Test compounds were prepared by diluting stock solutions (1.0 mg/mL in DMSO) in a mixture of mobile phases A:B (1:1 *v*/*v*) containing an internal standard (200 nM), achieving a final concentration of 1.0 μg/mL (final DMSO concentration ≤ 1%). The prepared solutions were transferred to a 96-well plate in duplicate, which was then sealed and introduced into the autosampler for analysis. Eight reference compounds with known LogD values, ranging from −1.86 to 6.1, were used to establish a calibration curve: acyclovir (Oakwood Chemical, Estill, SC, USA), atenolol (Sigma-Aldrich, St. Louis, MO, USA), antipyrine (Sigma-Aldrich, St. Louis, MO, USA), fluconazole (Sigma-Aldrich, St. Louis, MO, USA), metoprolol (Sigma-Aldrich, St. Louis, MO, USA), ketoconazole (Sigma-Aldrich, St. Louis, MO, USA), tolnaftate (Target Mol, Wellesley Hills, MA, USA), and amiodarone (Sigma-Aldrich, St. Louis, MO, USA). Additionally, loratadine (Sigma-Aldrich, St. Louis, MO, USA), LogD 4.4, and chloramphenicol (Sigma-Aldrich, St. Louis, MO, USA), LogD 1.14, were included as control compounds. These reference and control compounds were injected at the beginning and end of the experimental procedure. A calibration graph was obtained by plotting the retention times of the reference compounds against their respective LogD values. The resulting equation (y = mx + b) was used to calculate the eLogD_7.4_ values for the test compounds and controls. The retention time of each analyte was substituted into “x” in the equation, and the resulting “y” represented the corresponding eLogD value. The eLogD values of the control compounds were required to remain within ±0.5 of their established values. The determination coefficient (R^2^) of the calibration curve had to be ≥0.90 to ensure data reliability.

#### 4.5.5. Metabolic Stability in Mouse (CD-1) and Human Liver Microsomes

The metabolic stability of test compounds and controls [imipramine (Sigma-Aldrich, St. Louis, MO, USA), verapamil (Sigma-Aldrich, St. Louis, MO, USA), and warfarin (Sigma-Aldrich, St. Louis, MO, USA)] were assessed using human pooled liver human microsomes (20 mg/mL) (GIBCO, Frederick, MD, USA) and pooled liver CD1 mouse microsomes (20 mg/mL) (GIBCO, Frederick, MD, USA). Intermediate solutions of compounds were prepared by diluting stock solutions (10 mM in DMSO) in phosphate buffer (50 mM, pH 7.4) to a final concentration of 36 μM (final DMSO concentration ≤ 1%). Microsomal suspensions were thawed and diluted in phosphate buffer to obtain a final concentration of 0.25 mg/mL. A solution of NADPH (Sigma-Aldrich, St. Louis, MO, USA) at 0.5 μM was also prepared in phosphate buffer. The reaction mixture was prepared in a 96-well plate in the following order: 2.8 μL of intermediate compound solution, 185 μL of microsome suspension, and 10 μL of NADPH solution. The plate was incubated at 37 °C under shaking (100 rpm). Samples (25 μL) were collected at timepoints 0 (immediately after addition of NADPH), 5, 10, 20, 30, and 60 min. The reactions were stopped by adding quenching solution (75 μL) consisting of acetonitrile:methanol (1:1) with an internal standard (50 nM). Samples were centrifuged (3500 rpm, 30 min) to remove precipitated microsomal proteins, and the supernatant fractions were analyzed by an LCMS-8045 system (Shimazu, Kyoto, Japan). The chromatographic analysis was performed using a Supelco Ascentis Express C18 column (3 cm × 2.1 mm, 5 μm). The mobile phases consisted of water + 0.1% formic acid (A) and acetonitrile + 0.1% formic acid (B). The mobile phase was eluted in binary gradient mode as follows: 0 min, 95% A; 0.05 min, 95% A; 0.3 min, 2% A; 0.7 min, 2% A; 0.8 min, 95% A; 1.15 min, 95% A; 2.0 min, 95% A. The run time was 2 min, with a sample injection volume of 10 μL and a flow rate of 0.7 mL/min. Metabolic stability was assessed by calculating the peak area ratios (analyte/internal standard) and converting them into the percentage of the compound remaining, using the area ratio at time 0 as 100%. From these data, an exponential decay curve (y = e^(−kt)) of % remaining versus incubation time was obtained. The decay rate constant (k) was determined and used to calculate the half-life (T½ = ln(2)/k) in minutes and intrinsic clearance (CLint = k × 1000/0.25) in μL/min/mg using non-linear regression analysis.

## 5. Conclusions

In this work, 12 chalcones were synthesized, with yields ranging from 22% to 60%. Among them, 11 novel compounds were designed and obtained as boronic chalcones containing different substituents on the A-ring. The anticancer activity of all these compounds was evaluated against a HNC model cell line (SCC-25). Compound **5** emerged as the lead candidate due to its potent and selective cytotoxicity, exhibiting an IC_50_ of 17.9 µM against SCC-25 cells and 58.9 µM against normal cells (NOK-si cell line), resulting in a selectivity index (SI) of 3.29. Subsequently, this compound was also tested against the Detroit-562 cell line, a metastatic HNC model, and showed an IC_50_ of 33.1 µM. Based on these results, compound 5 was selected for further evaluation of its anti-inflammatory potential and ADME properties. Surprisingly, this compound showed an impressive anti-inflammatory profile. In comparison with compound 1, which lacks the boronic acid moiety, it was possible to determine the importance of this group in achieving the potent anti-inflammatory activity observed. Taken altogether, compound 5 stands out as the lead candidate due to its potent and selective cytotoxicity, p53-independent mechanism, and dual anticancer/anti-inflammatory activity. Furthermore, compound 5 fulfills Lipinski’s Rule of Five, with a determined *Log* P value of 2.92. Further modifications to improve metabolic stability will be necessary to enhance its drug-like properties for potential clinical applications. Our findings highlight the crucial role of the boronic acid moiety in improving the biological activity and optimizing the physicochemical properties of chalcone-based compounds, supporting its potential for drug development.

## Figures and Tables

**Table 1:** Cytotoxicity of compounds **1**–**12** (IC_50_ values in μM) and Log P values. Data are mean SEM of three independent experiments.

Compounds	SCC-25 IC_50_ (µM)	NOK-si IC_50_ (µM)	Detroit IC_50_ (µM)	SI *	eLog P ***
**(1)**	24.9 ± 1.09	101.5 ± 0.17	– **	4.08	3.73
**(2)**	133.7 ± 12.29	133.1 ± 3.25	–	1.00	2.68
**(3)**	45.2 ± 13.71	36.2 ± 10.69	–	1.88	1.49
**(4)**	9.8 ± 1.00	18.5 ± 4.43	31.6 ± 3.13	1.88	1.81
**(5)**	17.9 ± 0.35	58.9 ± 10.63	33.1 ± 5.10	3.29	2.92
**(6)**	27.2 ± 3.84	20.8 ± 2.17	–	0.77	2.30
**(7)**	41.1 ± 4.62	54.0 ± 10.98	–	1.31	3.46
**(8)**	33.4 ± 1.25	27.4 ± 3.05	–	0.82	4.68
**(9)**	108.3 ± 22.46	74.6 ± 14.97	–	0.69	1.18
**(10)**	43.8 ± 4.43	25.7 ± 11.56	–	0.59	1.64
**(11)**	65.9 ± 10.98	96.2 ± 21.26	–	1.46	2.81
**(12)**	38.4 ± 1.63	29.5 ± 6.08	–	0.77	2.16
**5-FU**	-	1800 ± 27.67	-	-	-

* SI = IC_50_ NOK-si/IC_50_ SCC-25. ** – = not determined. *** Experimental Log P.

**Table 2:** Cytotoxicity assessment of the evaluated compounds in HCT116 p53^+/+^ and HCT116 p53^−/−^ cell lines. IC_50_ values were determined after 48 h of treatment (growth obtained with vehicle was set as 100%). Data are mean SEM of three independent experiments.

Compounds	HCT p53^+/+^ IC_50_ (µM)	HCT p53^−/−^ IC_50_ (µM)
**(1)**	6.83 ± 1.15	7.56 ± 0.40
**(2)**	4.4 ± 0.53	3.63 ± 0.47
**(4)**	2.33 ± 0.20	2.16 ± 0.25
**(5)**	2.5 ± 0.17	2.16 ± 0.31
**(7)**	3.63 ± 0.40	4.2 ± 0.35
**(8)**	2.06 ± 0.45	1.70 ± 0.72
**(11)**	3.96 ± 0.55	3.8 ± 0.26

**Table 3:** Physicochemical and permeability properties determined for compound **5**.

LogD_7.4_	Kinect Solubility (KS) pH = 7.4 * (µg/mL)	PAMPA (Mean Pe ** 10^−6^ cm/s)	Human Liver Microsome (HLM)		Mouse Liver Microsome (MsLM)	
			T_1/2_ (min.)	Cl_int_(mic) (µg/min/mg)	T_1/2_ (min.)	Cl_int_(mic) (µg/min/mg)
3.92	>39.65	17.50	48.81	56.80	77.02	36.00

* Classification criteria: <10 µg/mL—Low solubility, 10–60 µg/mL—Moderate Solubility, >60 µg/mL—High Solubility. ** Pe >1.5 × 10^−6^ cm/s—High permeability, Pe < 1.5 × 10^−6^ cm/s—Low permeability.

## Data Availability

Data are contained within the article and Supplementary Materials.

## Supplementary Materials

The following supporting information can be downloaded at: https://www.mdpi.com/article/10.3390/molecules30143032/s1. Figure S1: Cell viability of 5-fluorouracil, Figure S2: Anti-inflammatory assay of compound **1**, Figure S3–S85: ^1^H, ^13^C, Chromatogram, HRMS and FTIR of compounds **1**–**12** (spectra of compounds).
