# Supplementary material for: Design and Synthesis of Boronic Chalcones with Dual Anticancer and Anti-Inflammatory Activity
Source: Molecules. 2025 Jul 19;30(14):3032. doi: 10.3390/molecules30143032 (PMC12301034; doi:10.3390/molecules30143032)
Supplement: Supplementary file 1 [file molecules-30-03032-s001.zip › molecules-3725060-supplementary.pdf]

Supplementary Material

Design and Synthesis of Boronic Chalcones with Dual Anticancer and Anti-Inflammatory Activity

Juliana Romano Lopes [1,*], Freddy Humberto Marin-Dett [1], Rita Alexandra Machado Silva [2], Rafael Consolin Chelucci [3], Lucília Saraiva [2], Maria Emília Sousa [4,5], Leonardo Luiz Gomes Ferreira [3], Adriano Defini Andricopulo [3], Paula Aboud Barbugli [6] and Jean Leandro Dos Santos [1,*]

1 School of Pharmaceutical Sciences, São Paulo State University (UNESP), Araraquara, 14800-903, SP, Brazil; freddy.m.dett@unesp.br
2 LAQV/REQUIMTE, Laboratório de Microbiologia, Departamento de Ciências Biológicas, Faculdade de Farmácia, Universidade do Porto, 4050-313 Porto, Portugal; up201904678@edu.fc.up.pt (R.A.M.S.); lucilia.saraiva@ff.up.pt (L.S.)
3 Laboratory of Medicinal and Computational Chemistry, Physics Institute of São Carlos, University of São Paulo (USP), São Carlos, 13563-120, SP, Brazil; rafaelchelucci@gmail.com (R.C.C.); leonardo@ifsc.usp.br (L.L.G.F.); aandrico@ifsc.usp.br (A.D.A.)
4 Laboratório de Química Orgânica e Farmacêutica, Departamento de Ciências Químicas, Faculdade de Farmácia, Universidade do Porto, 4050-313 Porto, Portugal; esousa@ff.up.pt
5 CIIMAR—Centro Interdisciplinar de Investigação Marinha e Ambiental, Terminal de Cruzeiros do Porto de Leixões, 4450-208 Matosinhos, Portugal
6 School of Dentistry, São Paulo State University (UNESP), Araraquara, 14801-385, SP, Brazil; paula.barbugli@unesp.br
* Correspondence: jromanolopes@gmail.com (J.R.L.); jean.santos@unesp.br (J.L.D.S.); Tel.: +55-16-3301-6962 (J.L.D.S.)

Table of contents	Figures	Pages
Cell viability of 5-fluorouracil	S1	3
Anti-inflammatory assay of compound **1**	S2	3
^{1}H, ^{13}C, Chromatogram, HRMS and FTIR of compound **1**	S3-S8	4-6
^{1}H, ^{13}C, Chromatogram, HRMS and FTIR of compound **2**	S9-S14	7-9
^{1}H, ^{13}C, Chromatogram, HRMS and FTIR of compound **3**	S15-S22	10-13
^{1}H, ^{13}C, Chromatogram, HRMS and FTIR of compound **4**	S23-S29	11-16
^{1}H, ^{13}C, Chromatogram, HRMS and FTIR of compound **5**	S30-S36	17-19
^{1}H, ^{13}C, Chromatogram, HRMS and FTIR of compound **6**	S37-S43	20-22
^{1}H, ^{13}C, Chromatogram, HRMS and FTIR of compound **7**	S44-S50	23-25
^{1}H, ^{13}C, Chromatogram, HRMS and FTIR of compound **8**	S51-S57	26-28

^{1}H, ^{13}C, Chromatogram, HRMS and FTIR of compound **9**	S58-S64	29-31
^{1}H, ^{13}C, Chromatogram, HRMS and FTIR of compound **10**	S65-S71	32-34
^{1}H, ^{13}C, Chromatogram, HRMS and FTIR of compound **11**	S72-S78	35-37
^{1}H, ^{13}C, Chromatogram, HRMS and FTIR of compound **12**	S79-S85	38-40

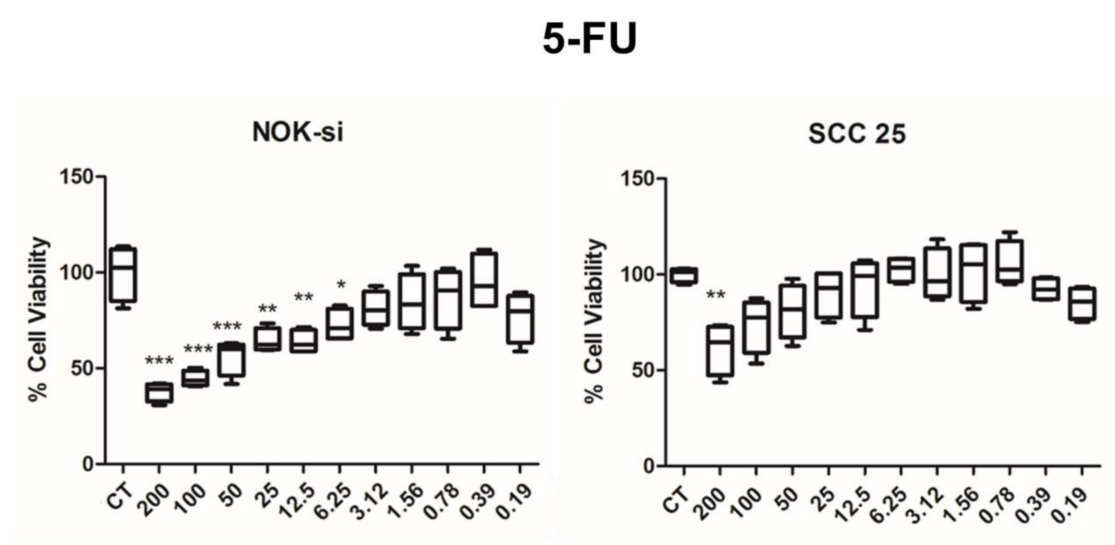

Figure S1. Cell viability of 5-fluorouracil (5-FU) evaluated on NOK-si and SCC-25 cell lines. *p<0.05, **p<0.01, ***p<0.001.

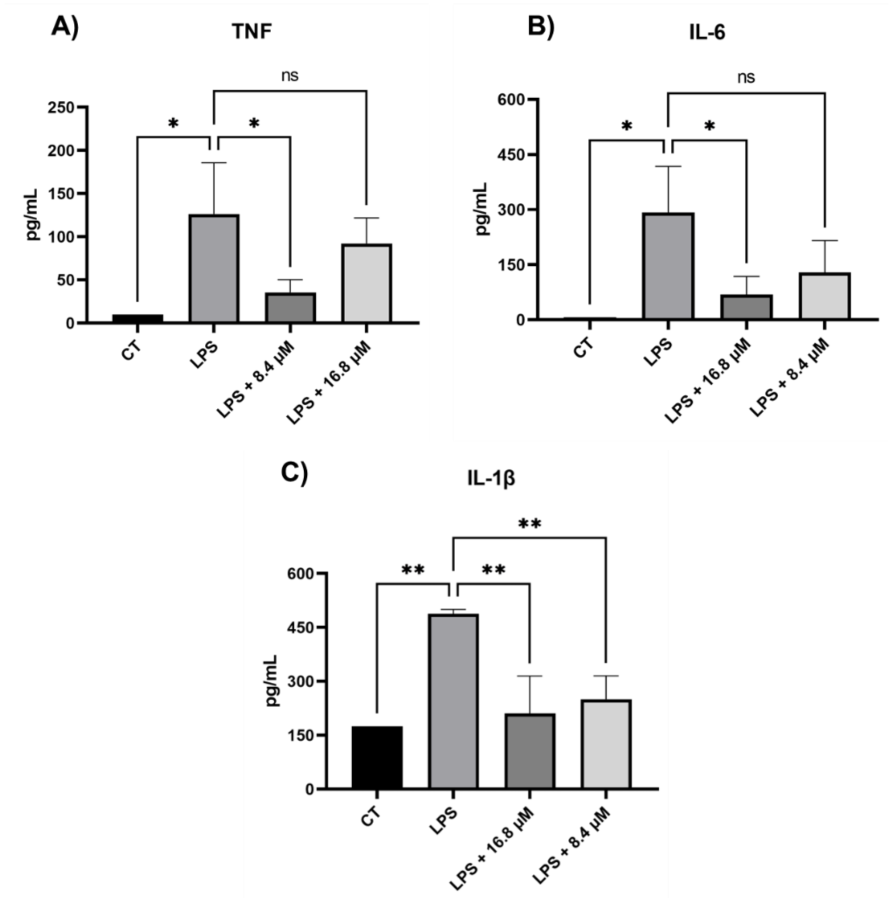

Figure S2. Evaluation of chalcone **1** in reducing levels of A) TNF, B) IL-6 and C) IL-1β in LPS-stimulated THP-1 cells at two different concentrations. Control (CT) = THP-1 cells not stimulated with LPS. *p<0.05, **p<0.01, ***ns*** = not significant

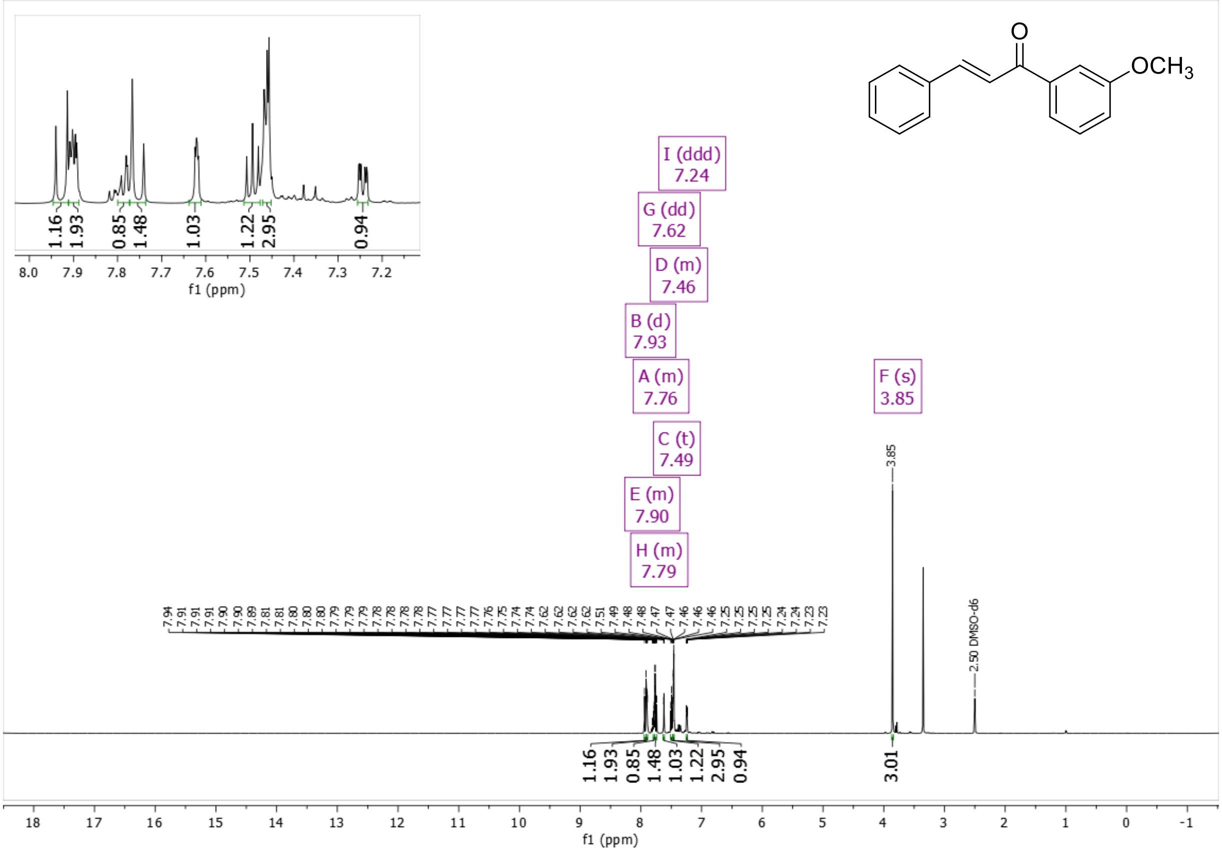

Figure S3. ^{1}H-NMR Spectrum of compound **1** (600 MHz DMSO-*d*6).

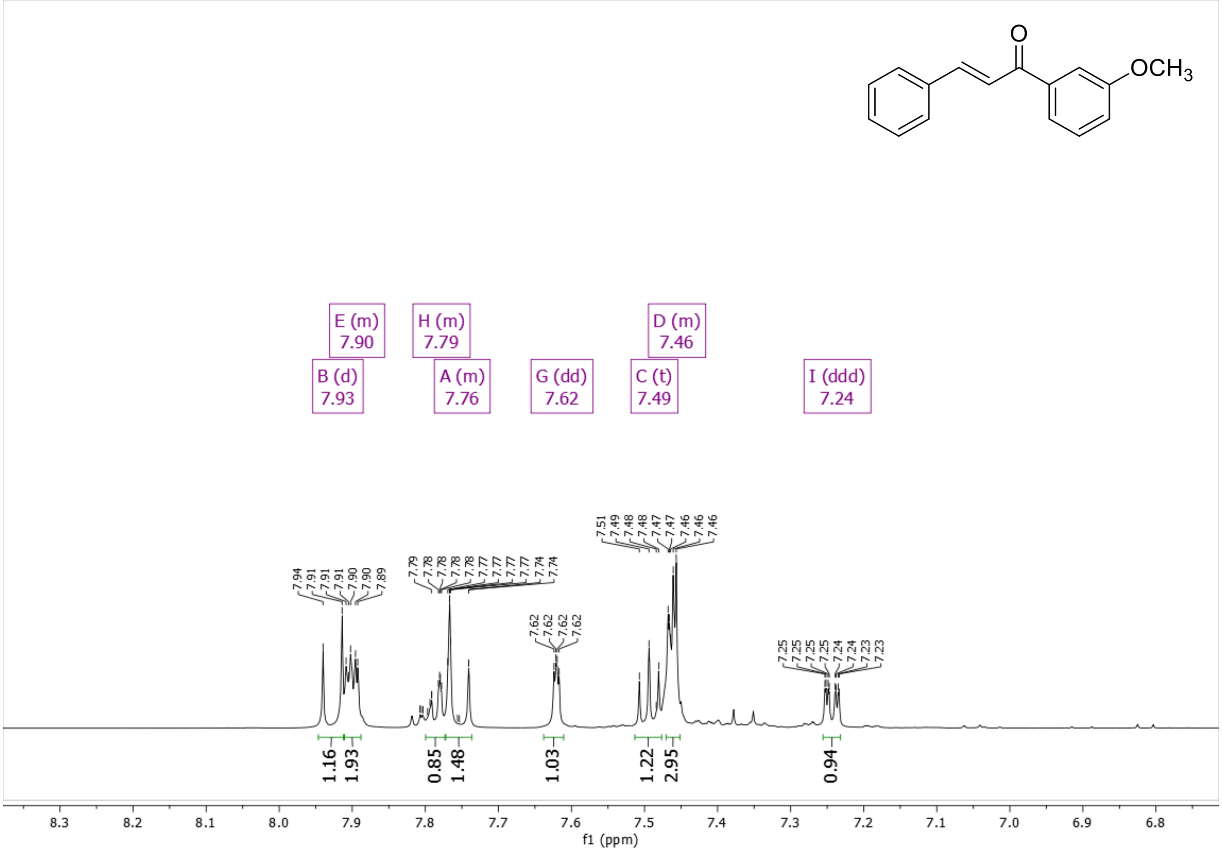

Figure S4. ^{1}H-NMR Spectrum of compound **1** (600 MHz DMSO-*d*6).

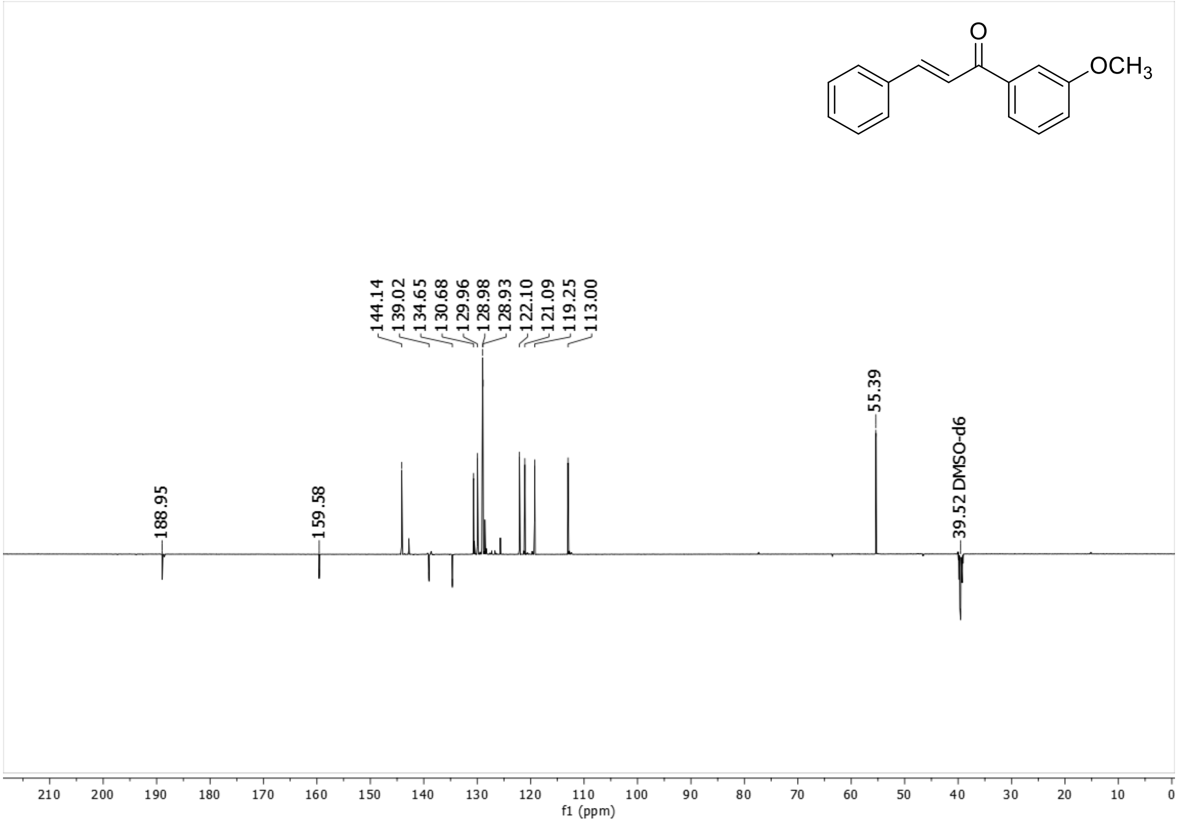

Figure S5. DEPTQ-NMR Spectrum of compound **1** (150 MHz DMSO-*d*6).

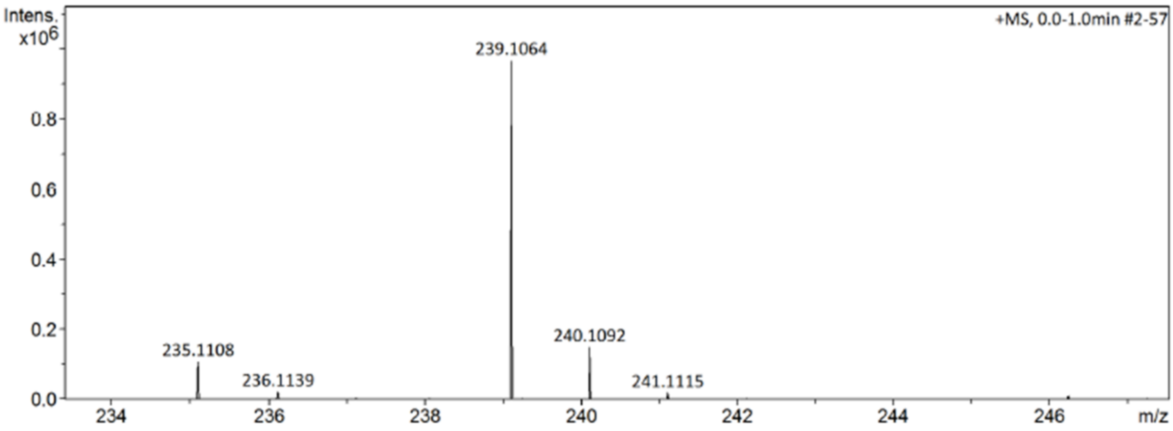

Figure S6. HRMS Spectrum of compound **1** (ESI+).

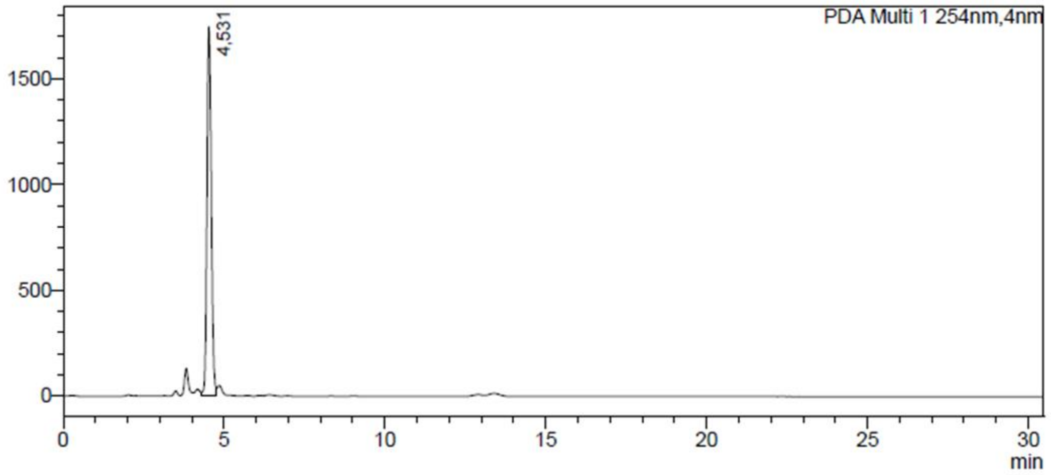

Figure S7. Chromatogram of compound **1**.

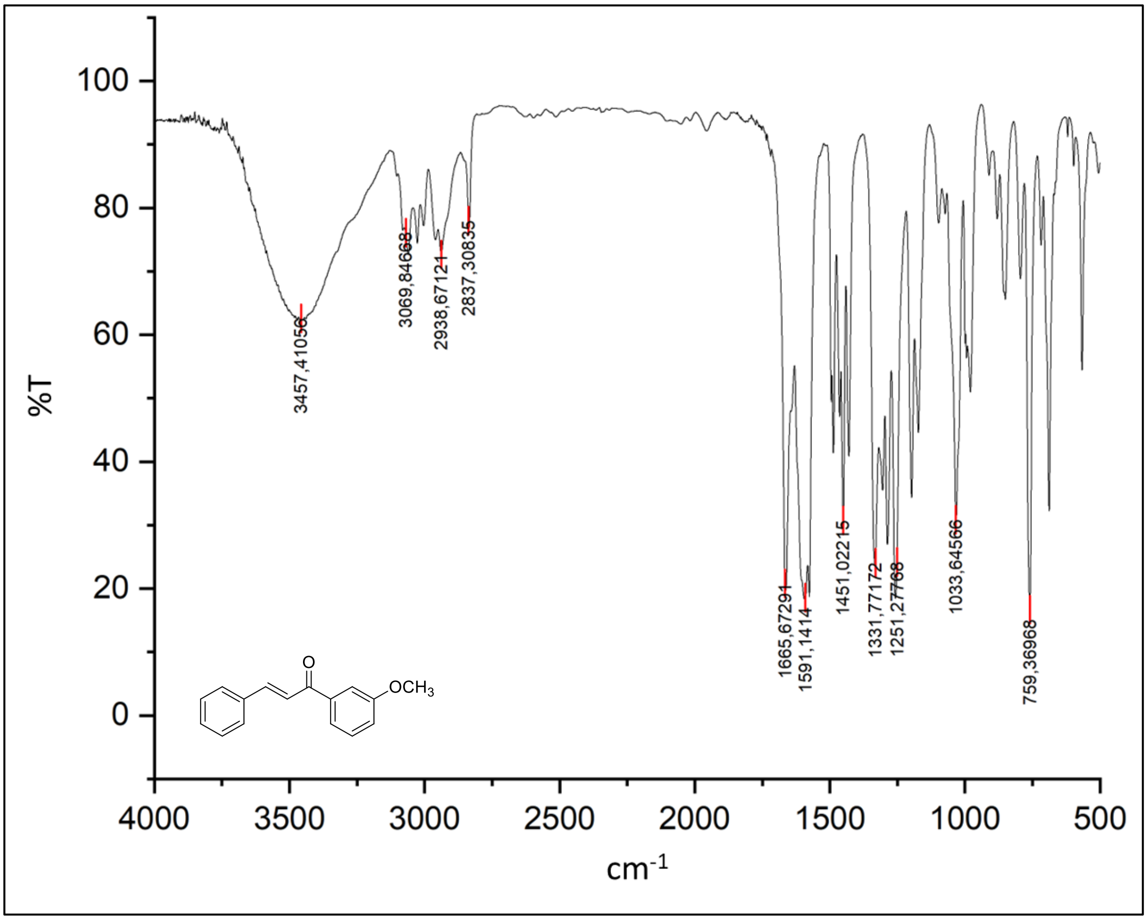

Figure S8. FTIR Spectrum of compound **1**.

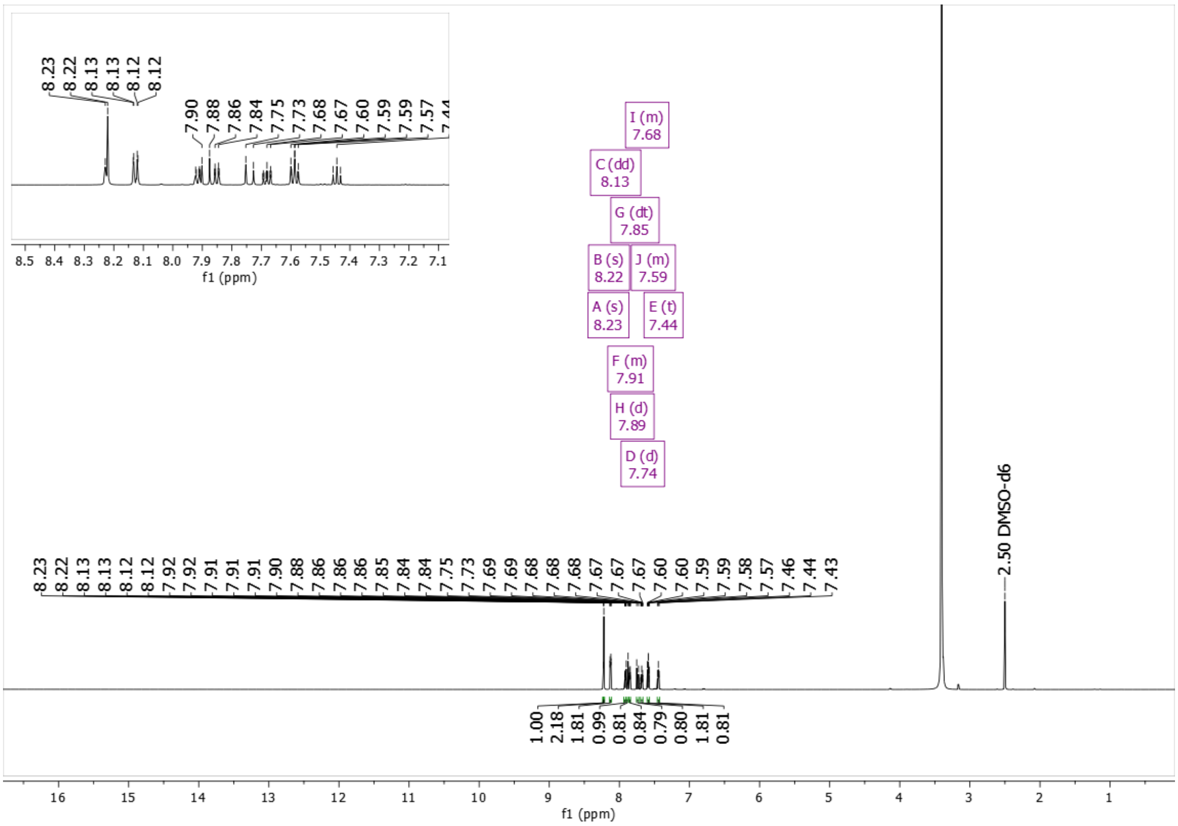

Figure S9. ^{1}H-NMR Spectrum of compound **2** (600 MHz DMSO-*d*6).

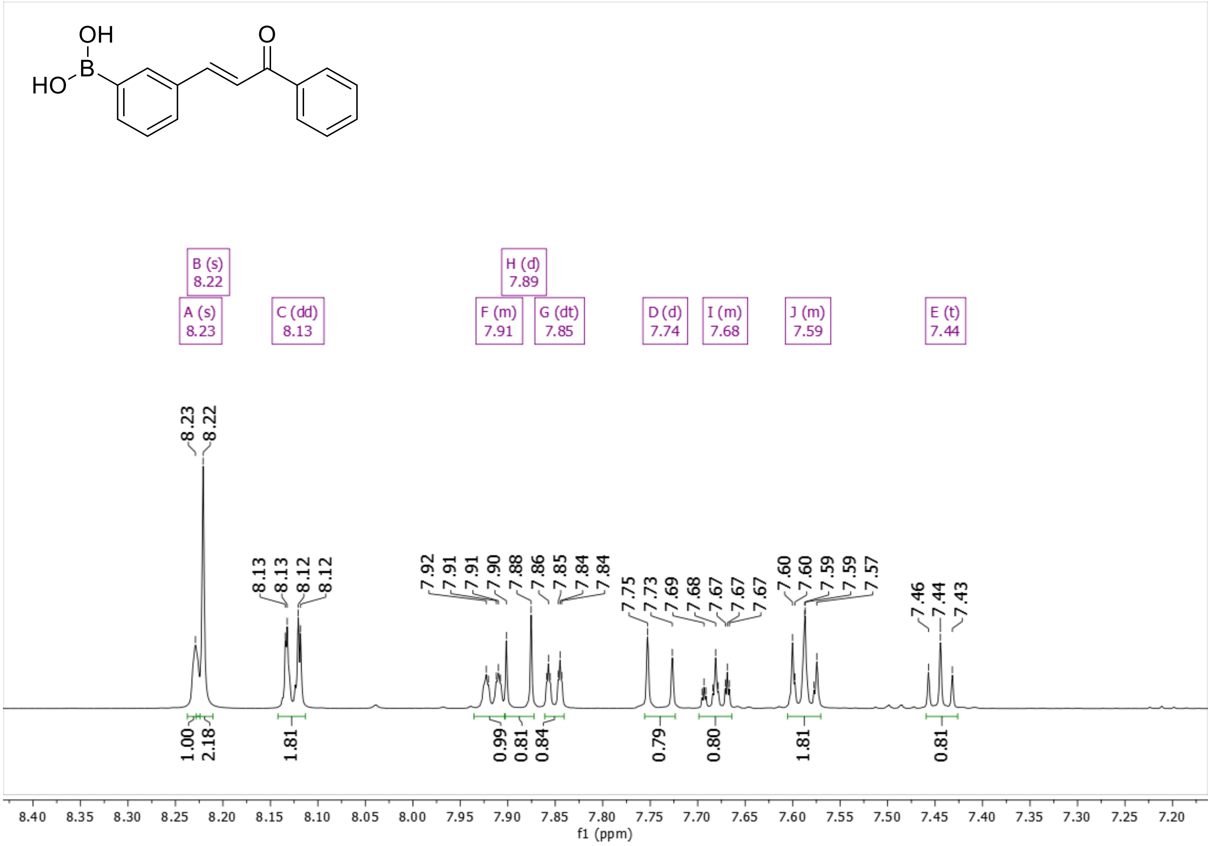

Figure S10. ^{1}H-NMR Spectrum of compound **2** (600 MHz DMSO-*d*6).

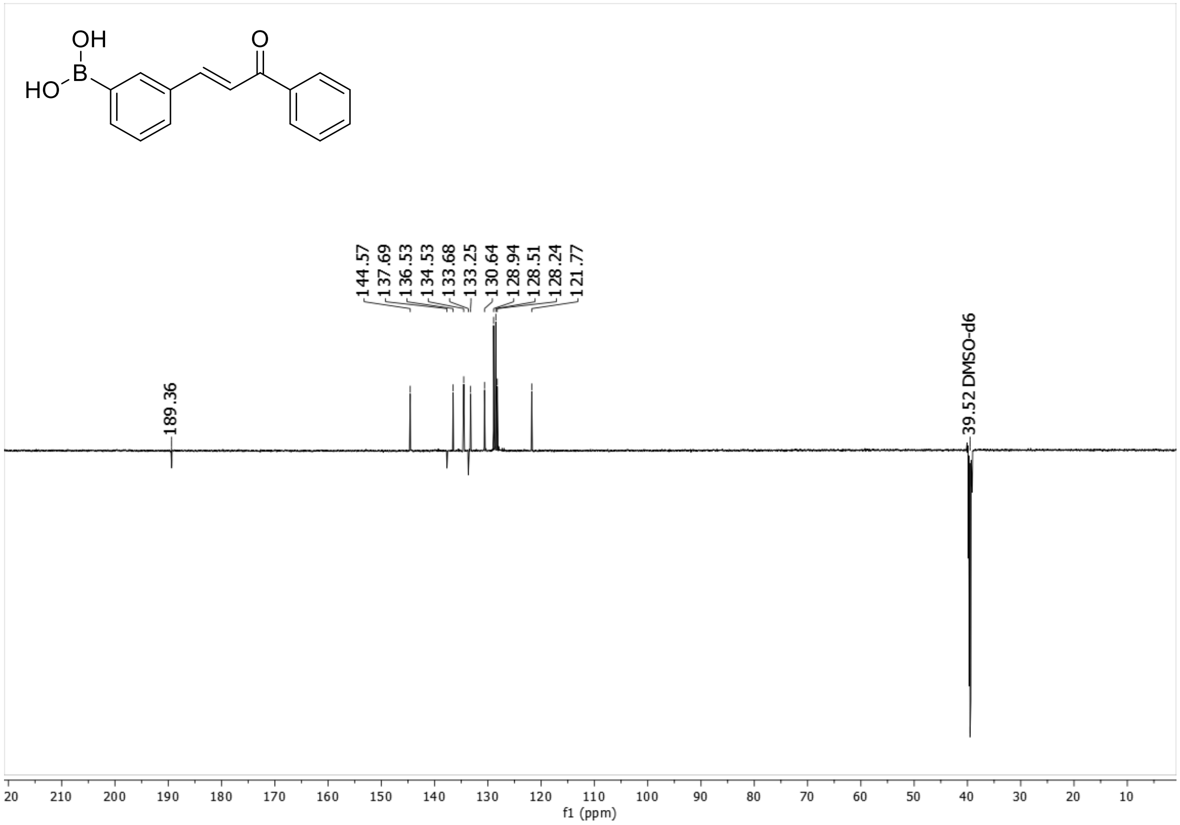

Figure S11. DEPTQ-NMR Spectrum of compound **2** (150 MHz DMSO-*d*6).

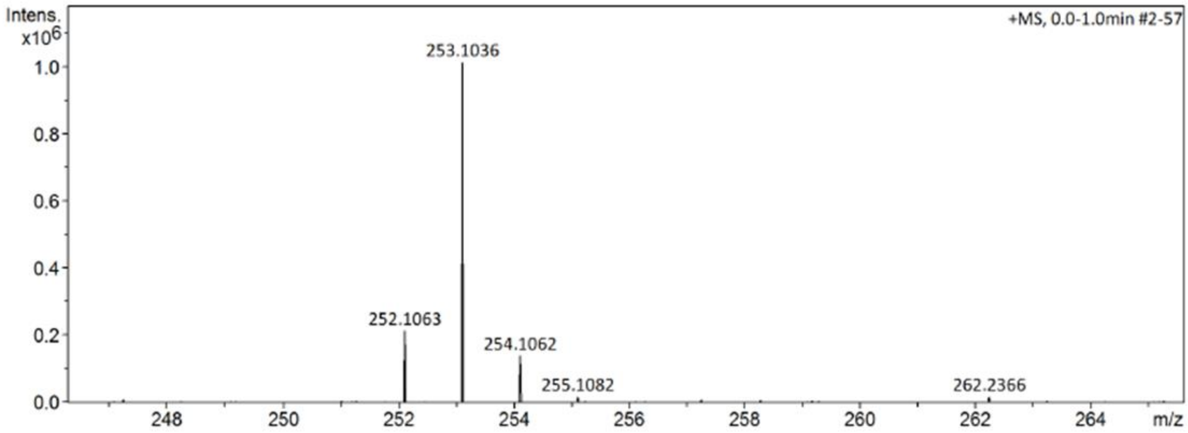

Figure S12. HRMS Spectrum of compound **2** (ESI+).

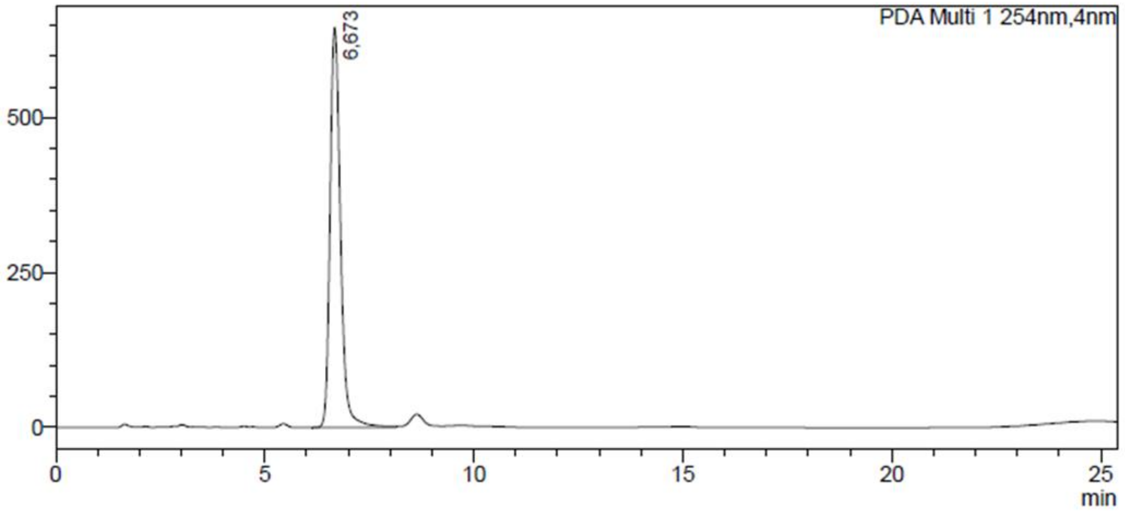

Figure S13. Chromatogram of compound **2**.

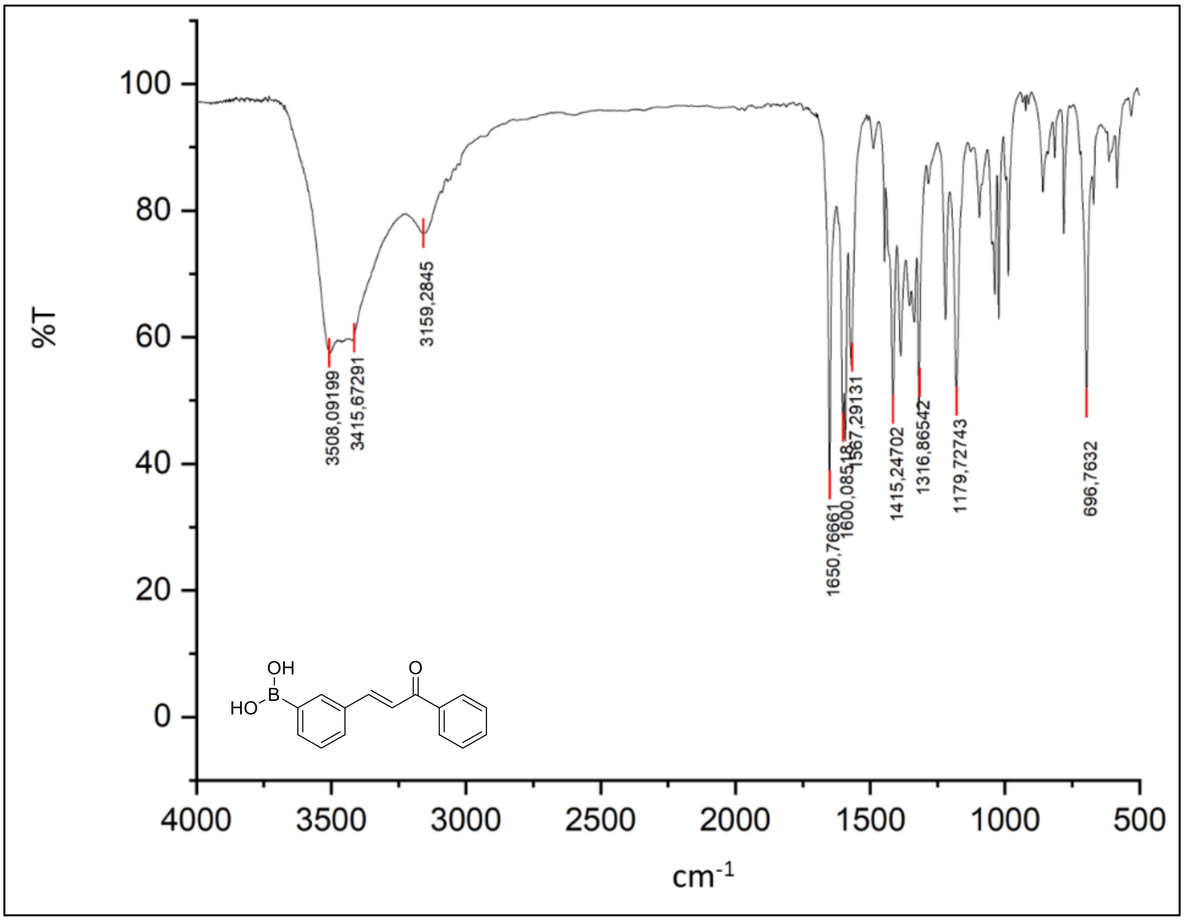

Figure S14. FTIR Spectrum of compound **2**.

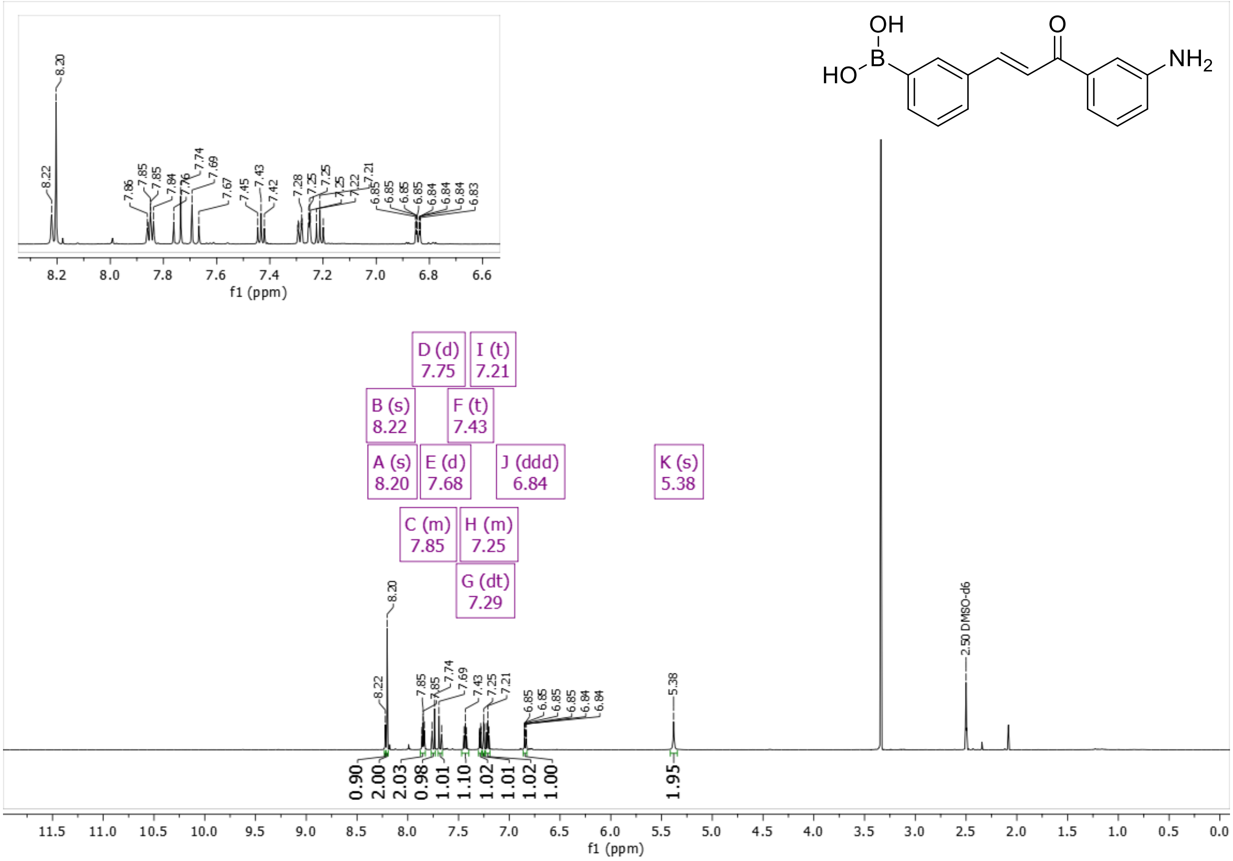

Figure S15. ^{1}H-NMR Spectrum of compound **3** (600 MHz DMSO-*d*6).

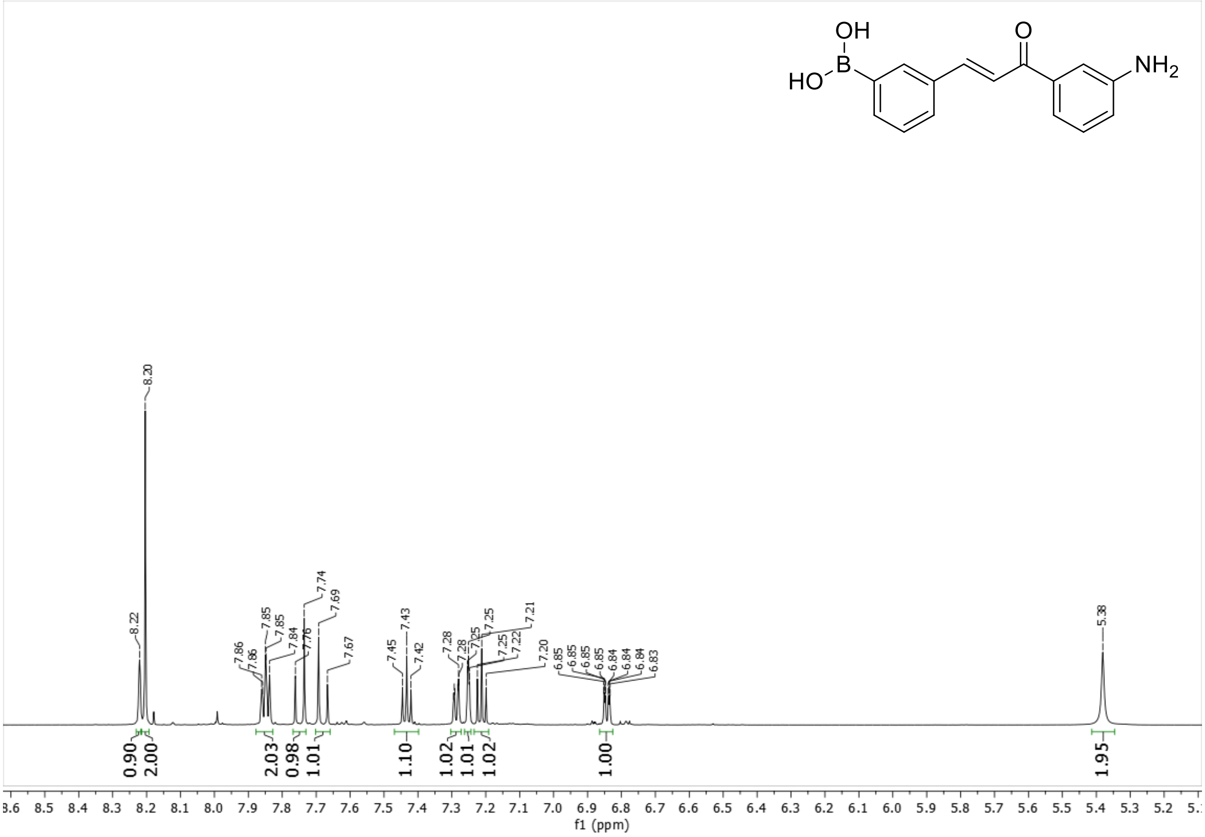

Figure S16. ^{1}H-NMR Spectrum of compound **3** (600 MHz DMSO-*d*6).

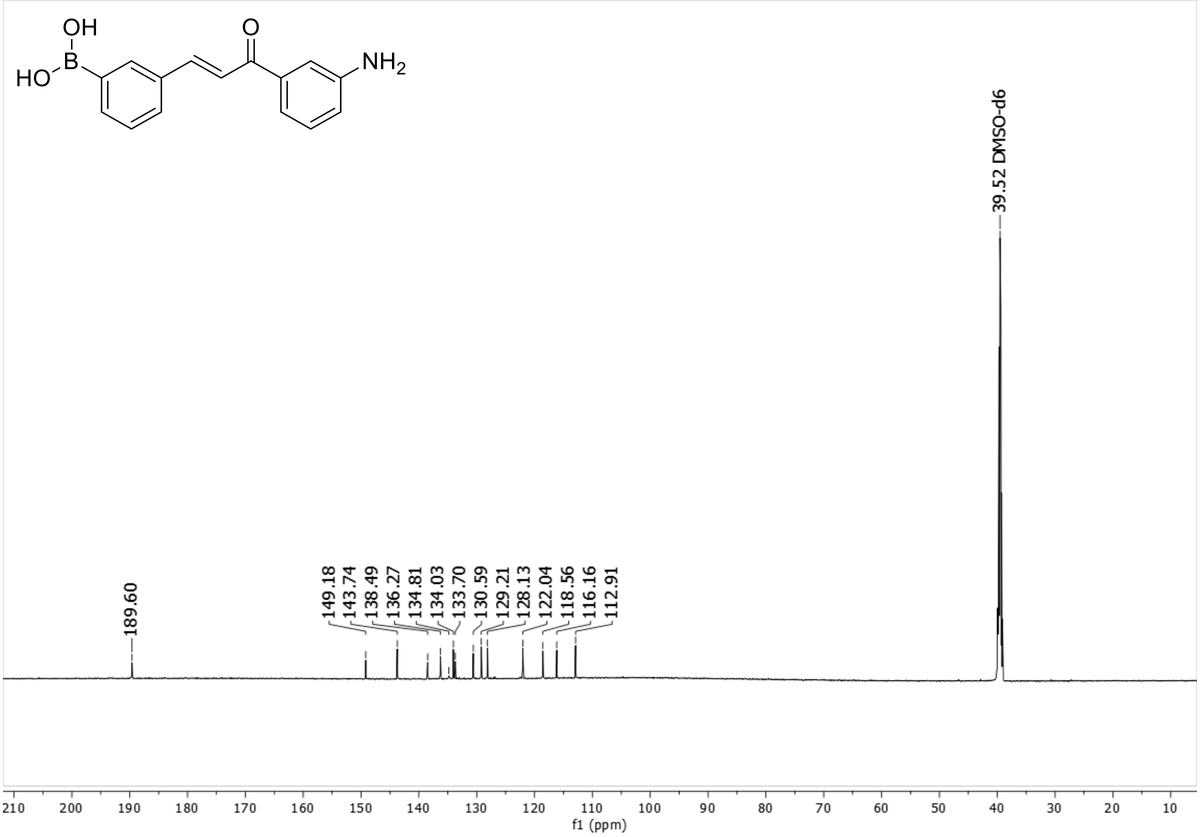

Figure S17. ^{13}C-NMR Spectrum of compound **3** (150 MHz DMSO-*d6*).

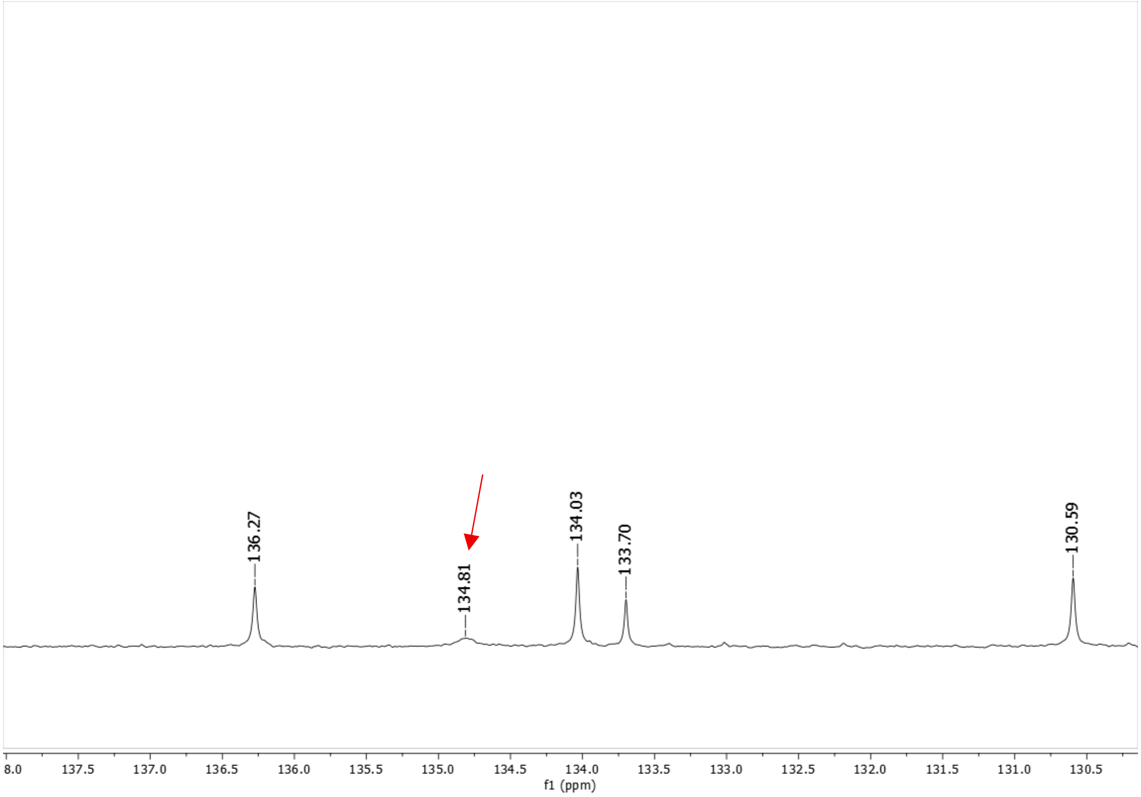

Figure S18. ^{13}C-NMR Spectrum of compound **3** (150 MHz DMSO-*d6*). The red arrow indicates the possible signal of the quaternary carbon directly bonded to boron (C–B).

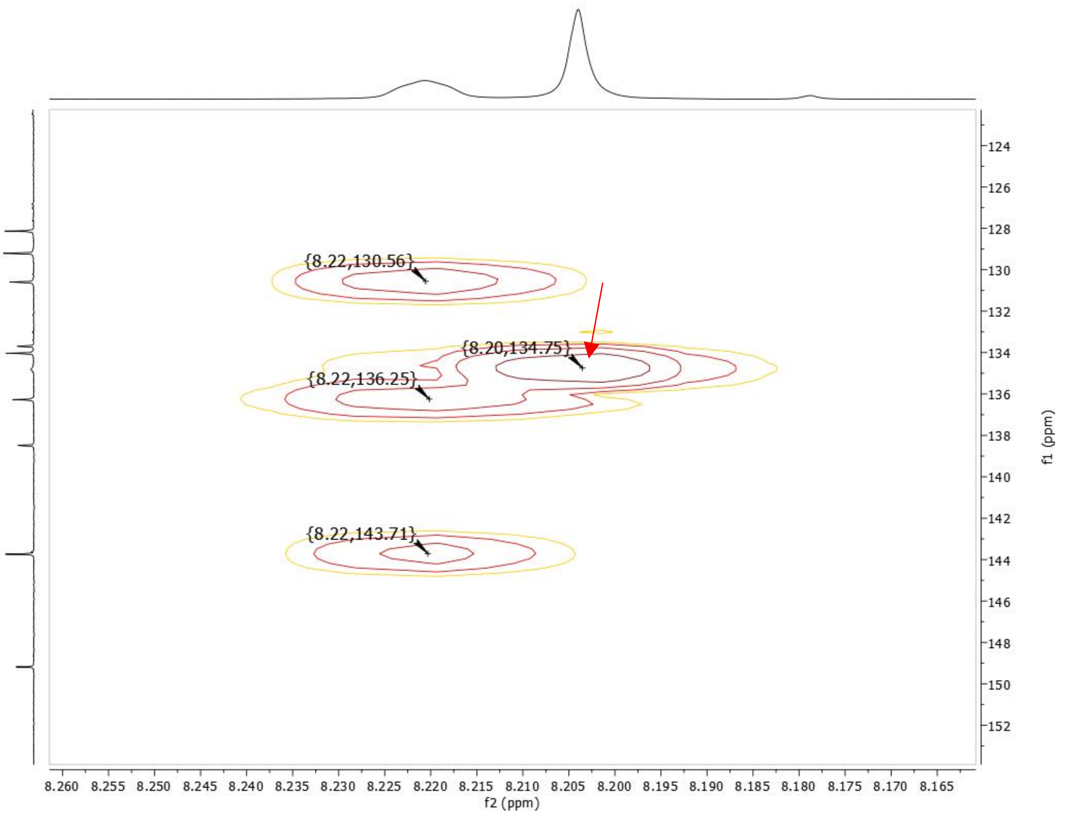

Figure S19. HMBC-NMR Spectrum of compound **3** (150 MHz DMSO-*d*6). The red arrow indicates the possible signal of the quaternary carbon directly bonded to boron (C–B).

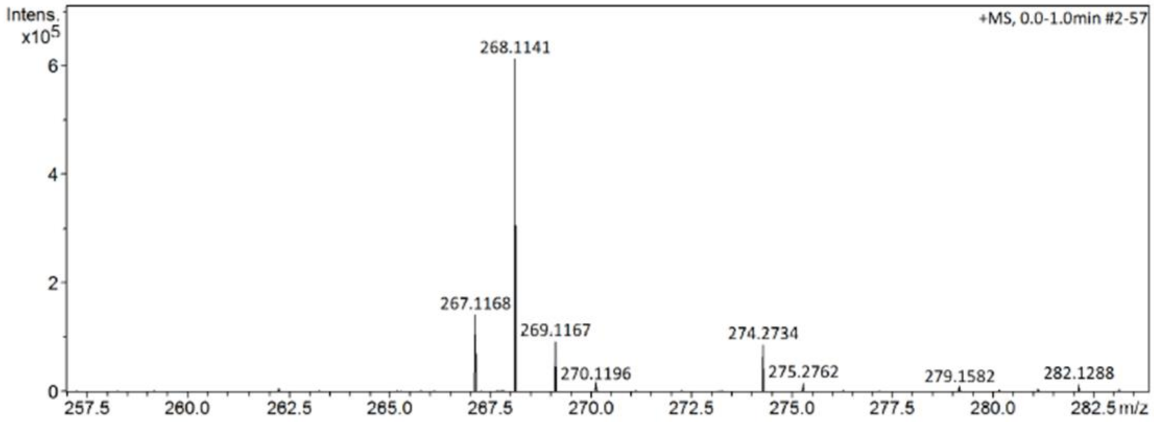

Figure S20. HRMS Spectrum of compound **3** (ESI+).

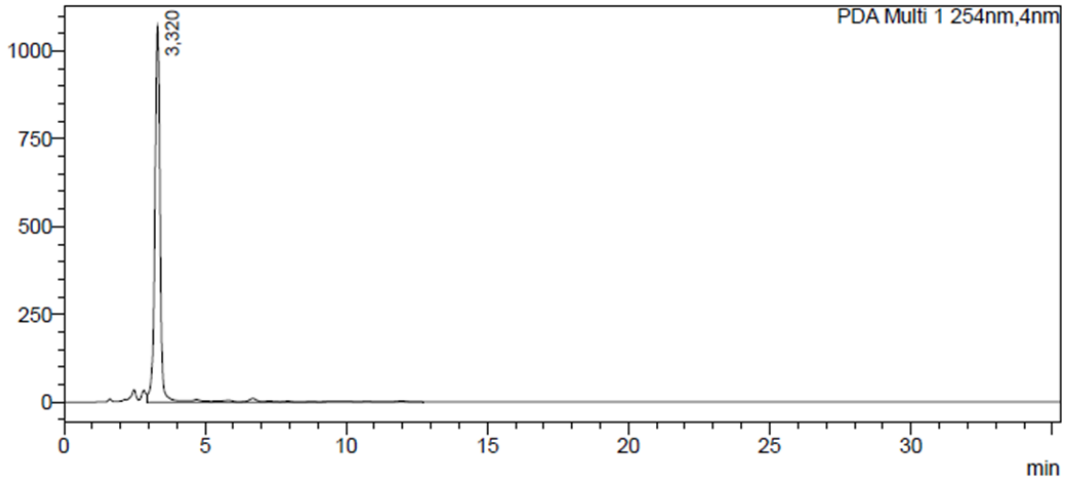

Figure S21. Chromatogram of compound **3**.

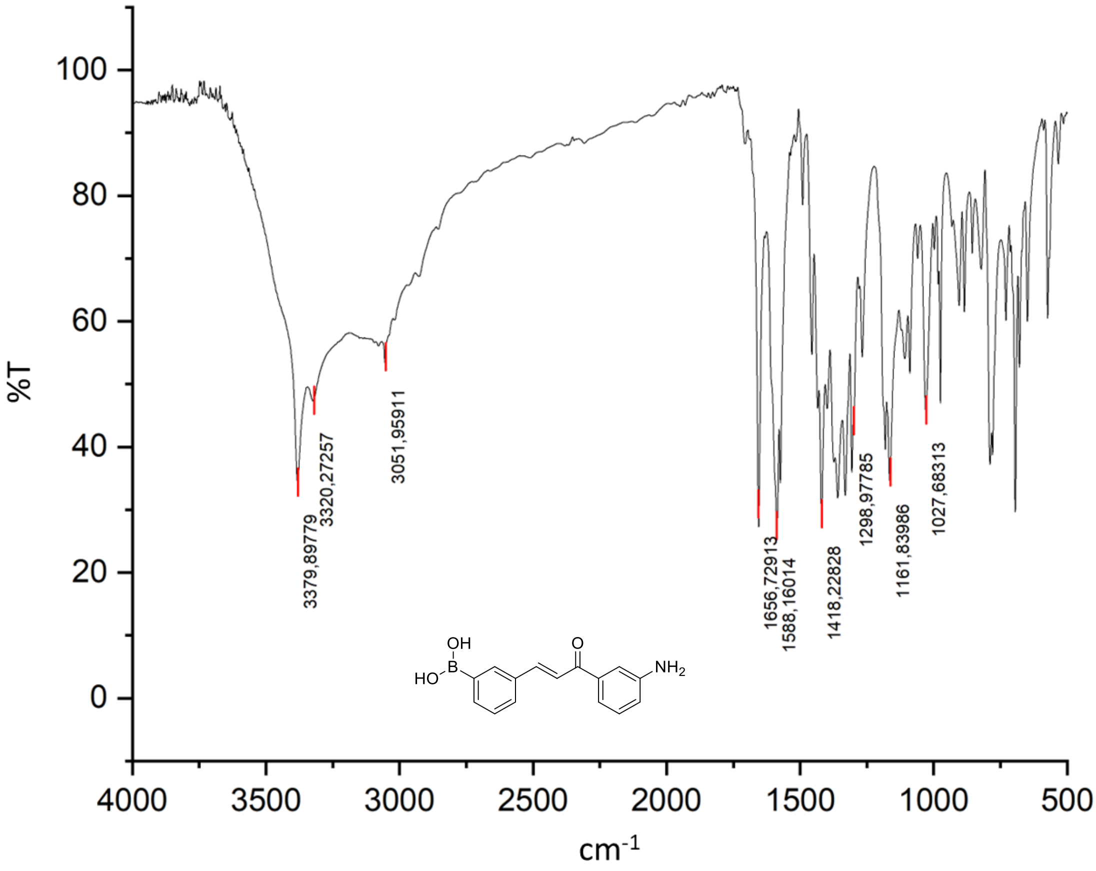

Figure S22. FTIR Spectrum of compound **3**.

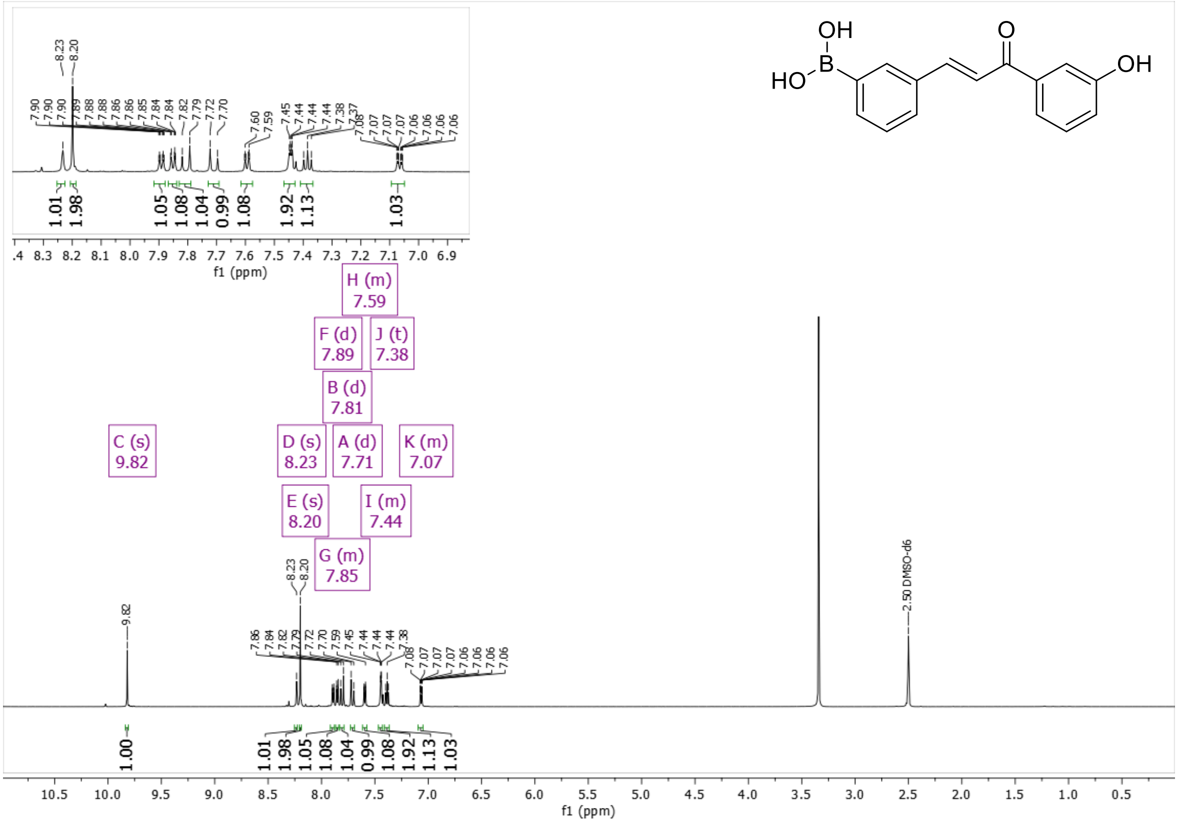

Figure S23. ^{1}H-NMR Spectrum of compound **4** (600 MHz DMSO-*d*6).

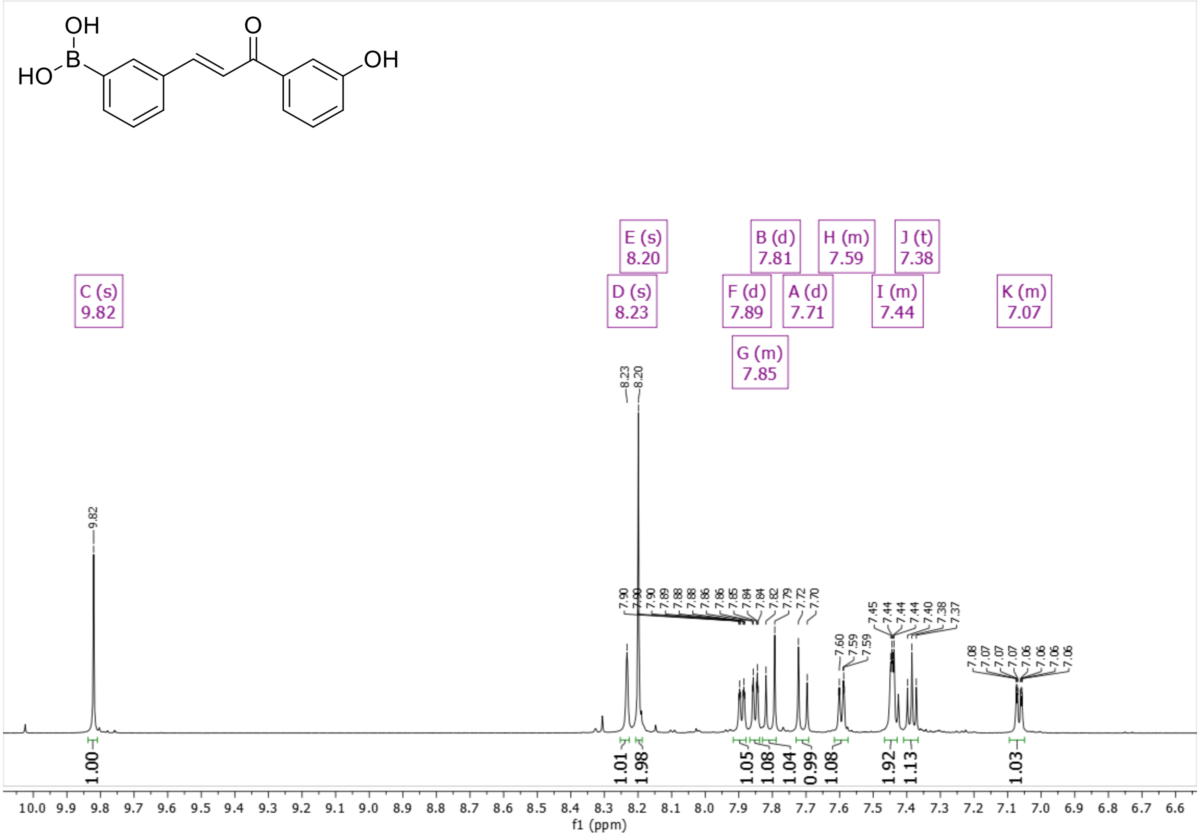

Figure S24. ^{1}H-NMR Spectrum of compound **4** (600 MHz DMSO-*d*6).

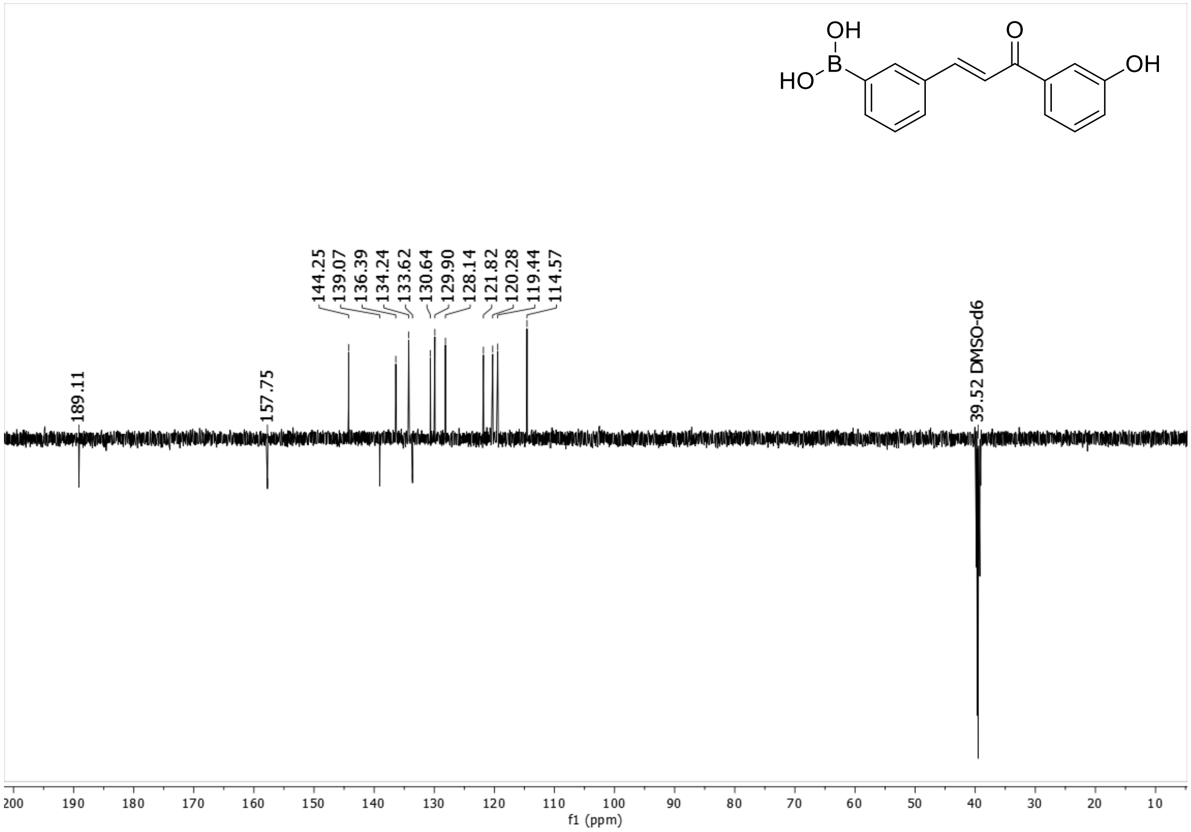

Figure S25.^{13}C-NMR Spectrum of compound **4** (150 MHz DMSO-*d6*).

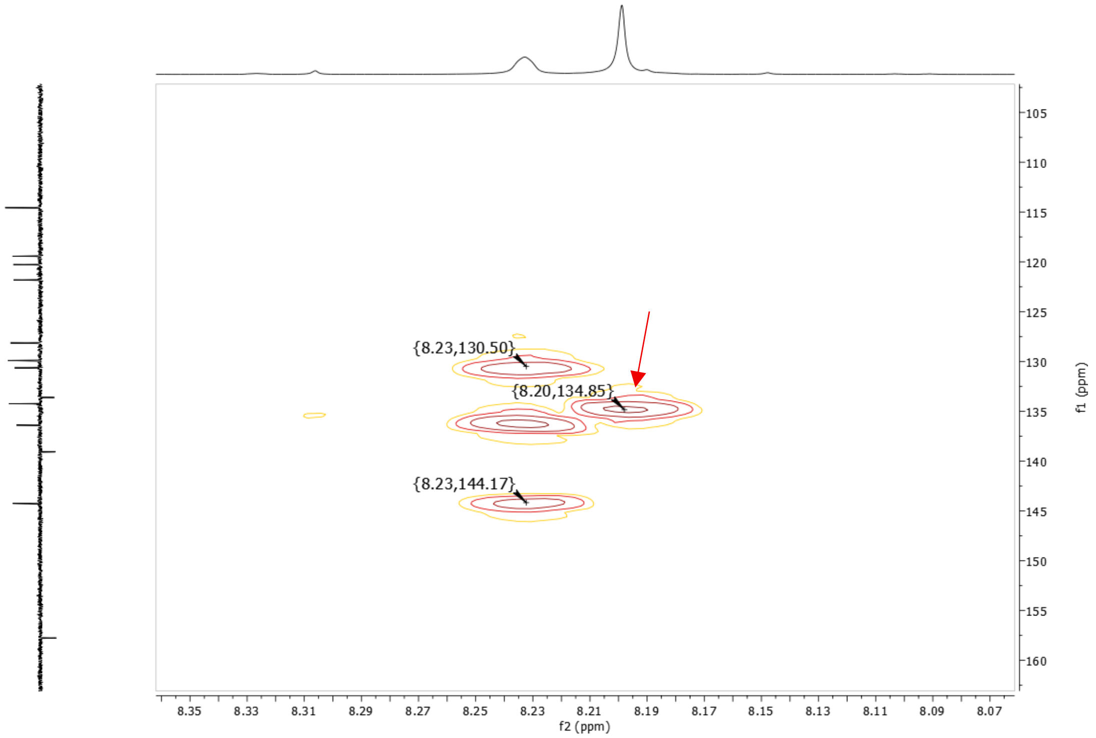

Figure S26. HMBC-NMR Spectrum of compound **4** (150 MHz DMSO-*d6*). The red arrow indicates the possible signal of the quaternary carbon directly bonded to boron (C–B).

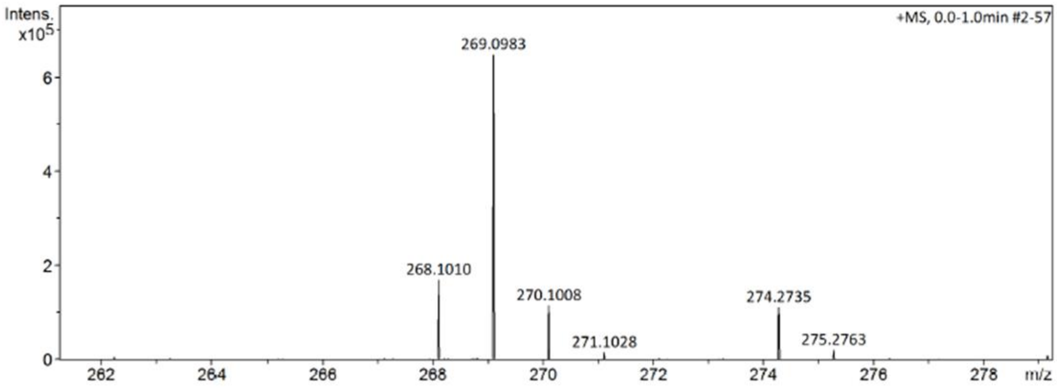

Figure S27. HRMS Spectrum of compound **4** (ESI+).

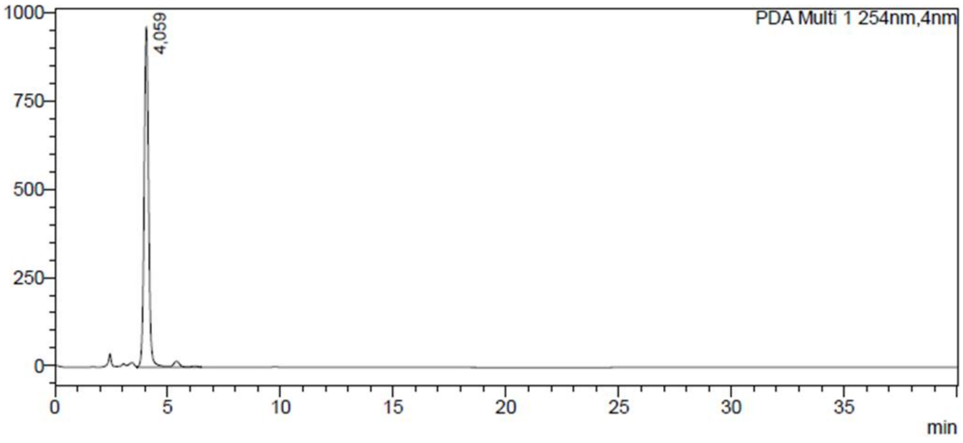

Figure S28. Chromatogram of compound **4**.

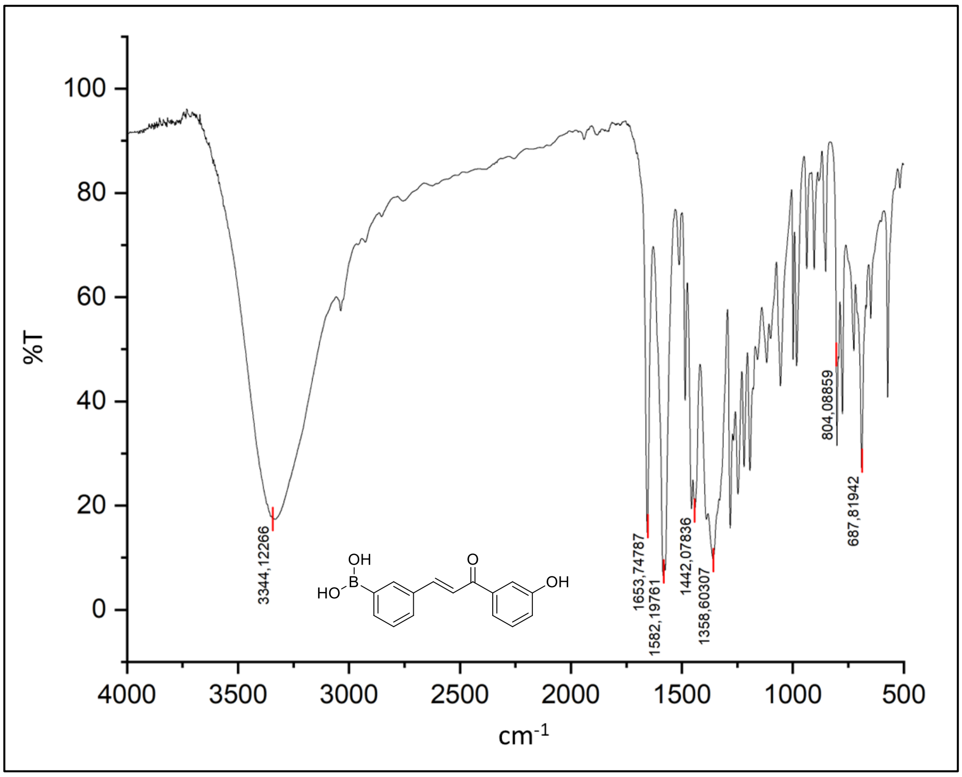

Figure S29. FTIR Spectrum of compound **4**

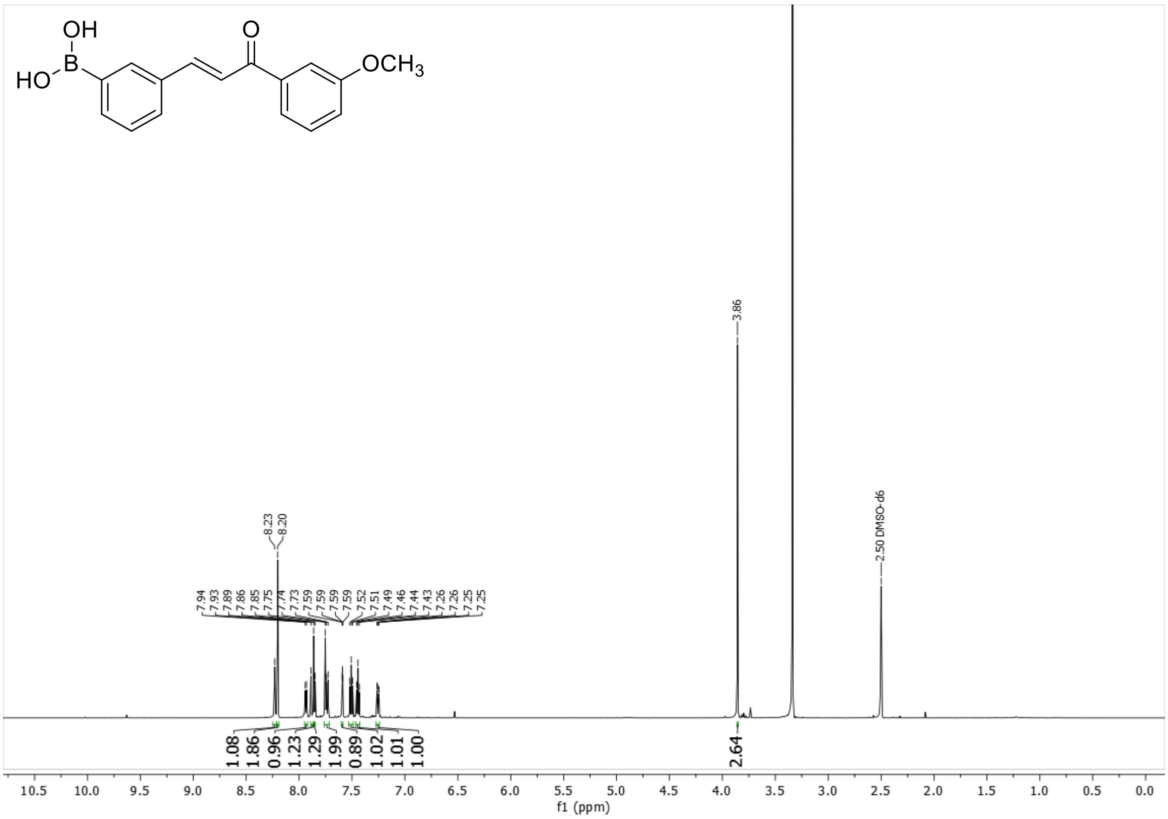

Figure S30. ^{1}H-NMR Spectrum of compound **5** (600 MHz DMSO-*d*6).

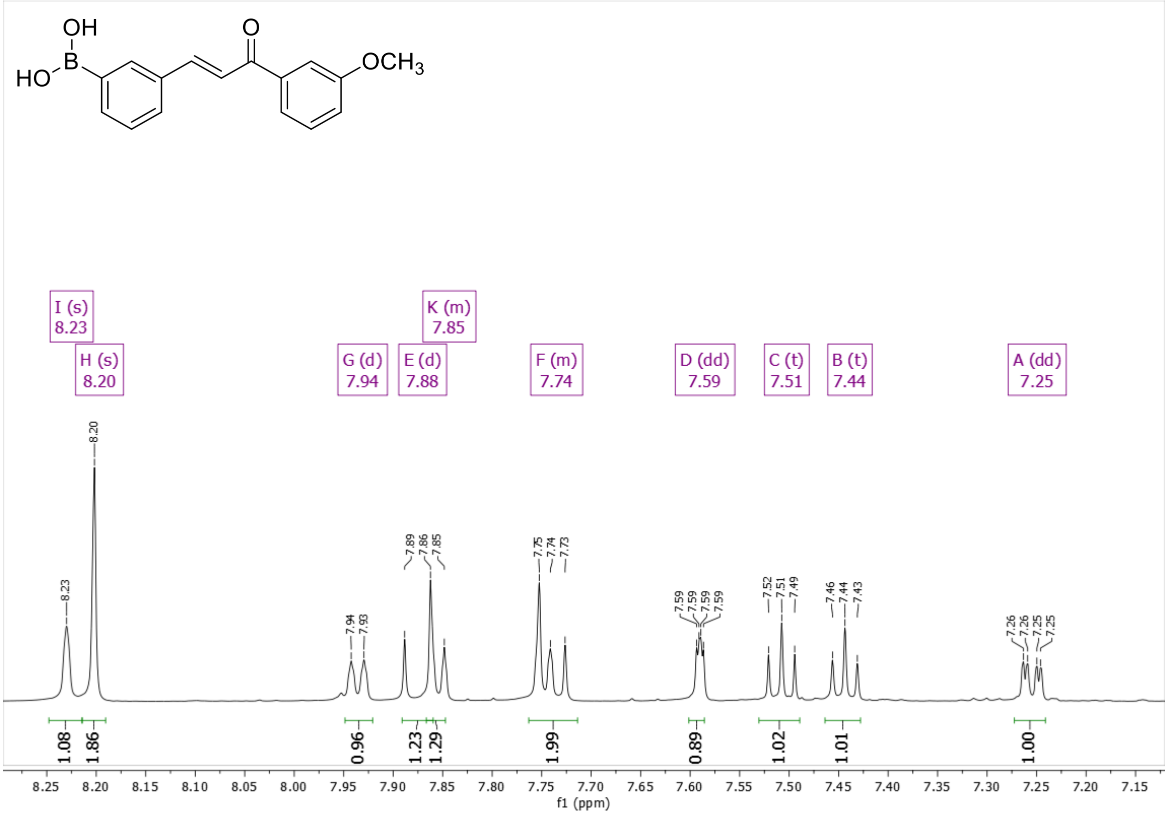

Figure S31. ^{1}H-NMR Spectrum of compound **5** (600 MHz DMSO-*d*6).

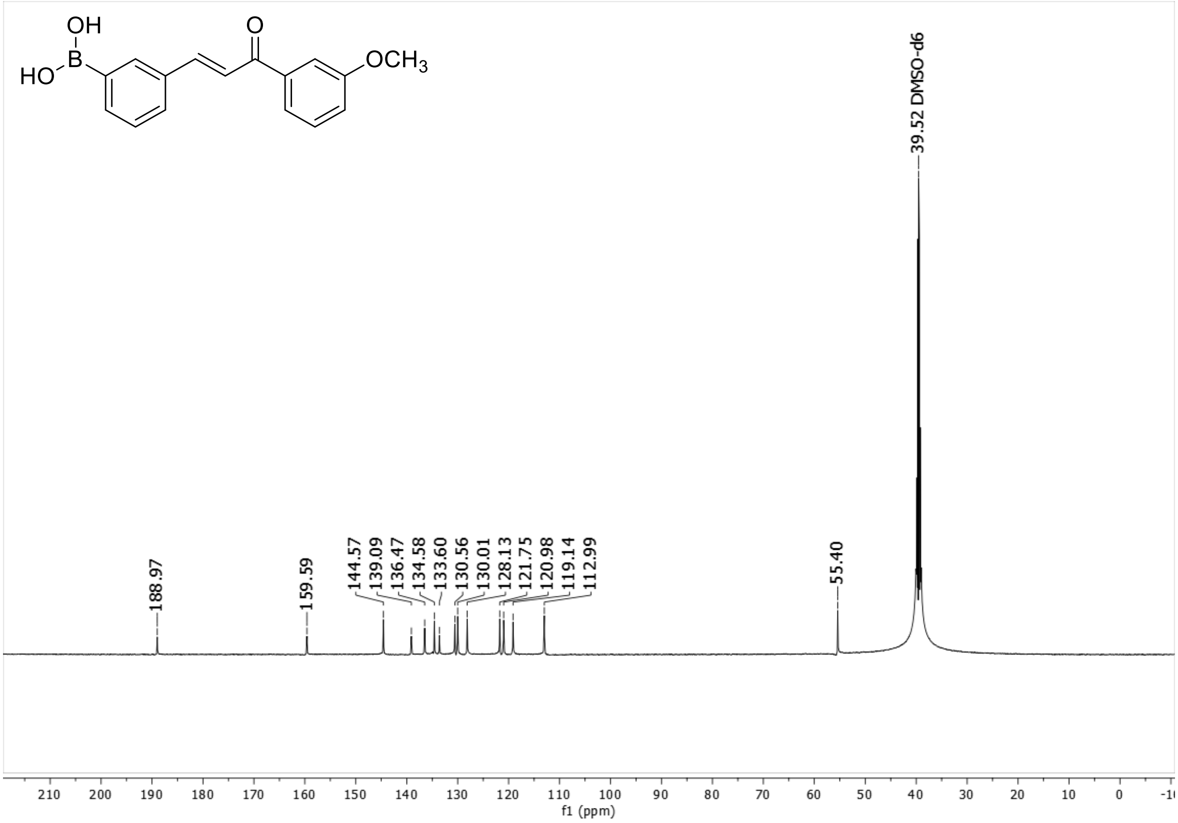

Figure S32. ^{13}C-NMR Spectrum of compound **5** (150 MHz DMSO-*d*6).

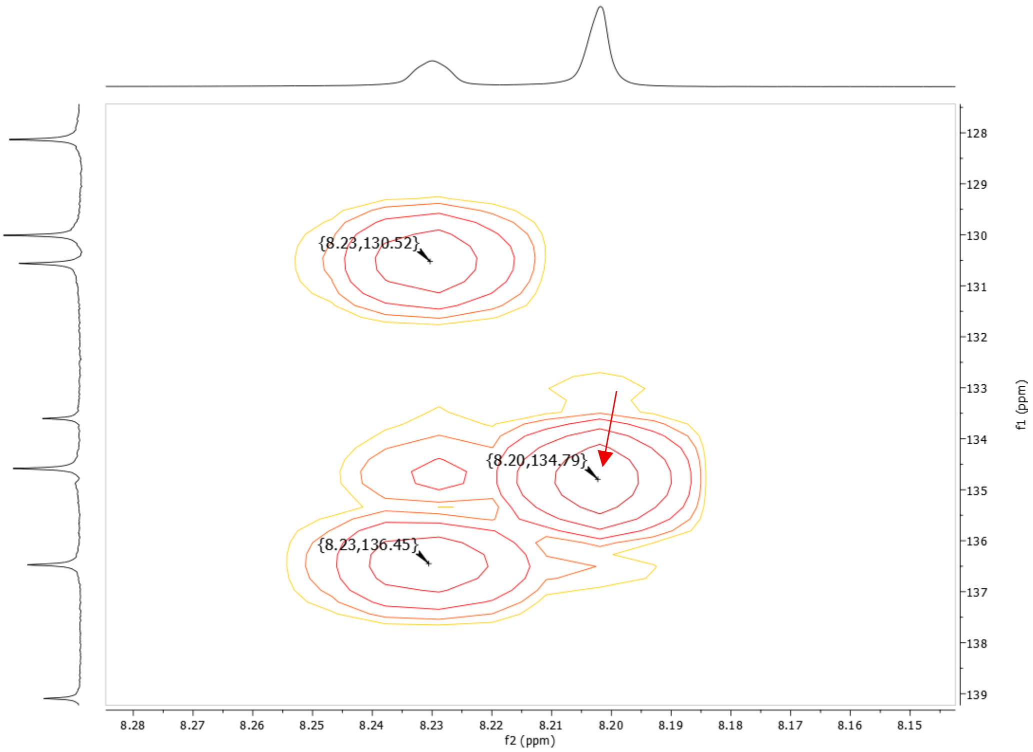

Figure S33. HMBC-NMR Spectrum of compound **5** (150 MHz DMSO-*d*6). The red arrow indicates the possible signal of the quaternary carbon directly bonded to boron (C–B).

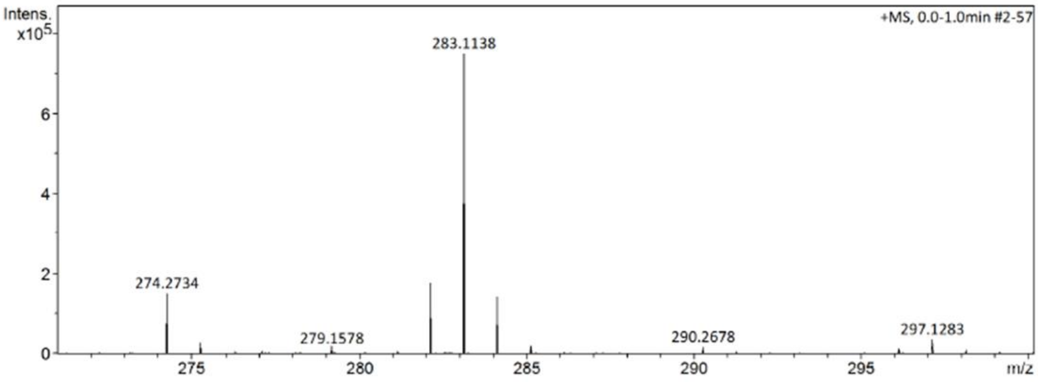

Figure S34. HRMS Spectrum of compound **5** (ESI+).

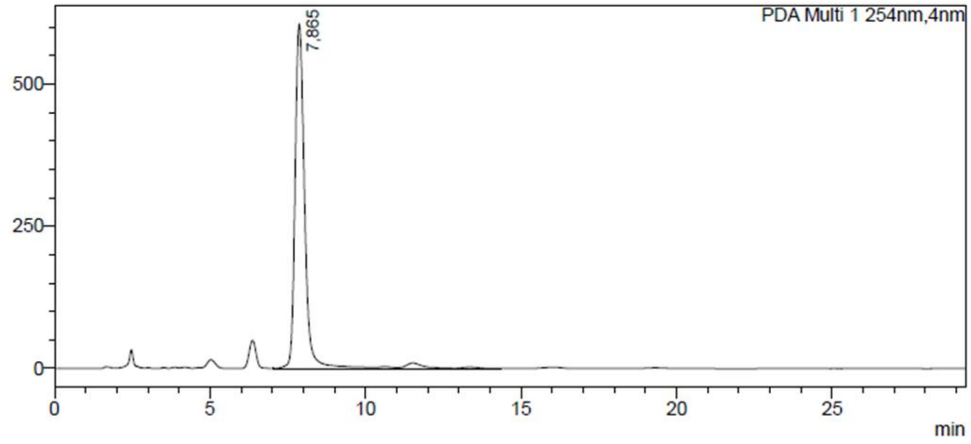

Figure S35. Chromatogram of compound **5**.

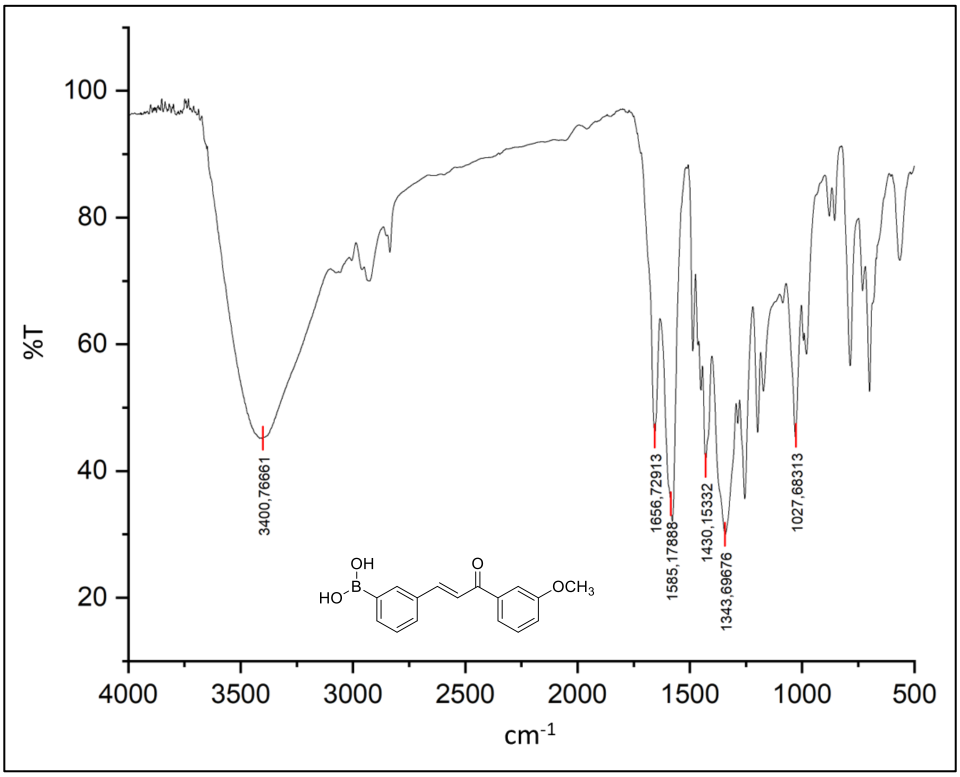

Figure S36. FTIR Spectrum of compound **5.**

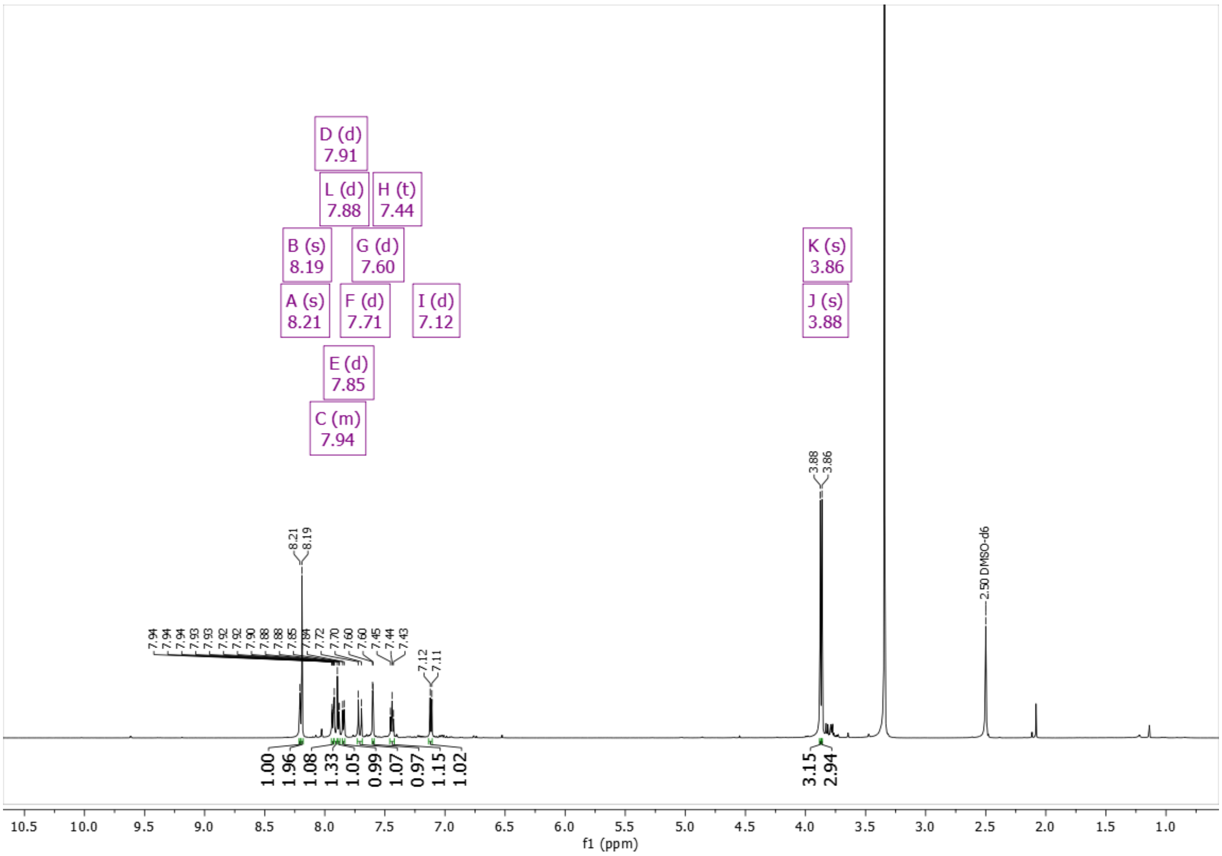

Figure S37. ^{1}H-NMR Spectrum of compound **6** (600 MHz DMSO-*d*6).

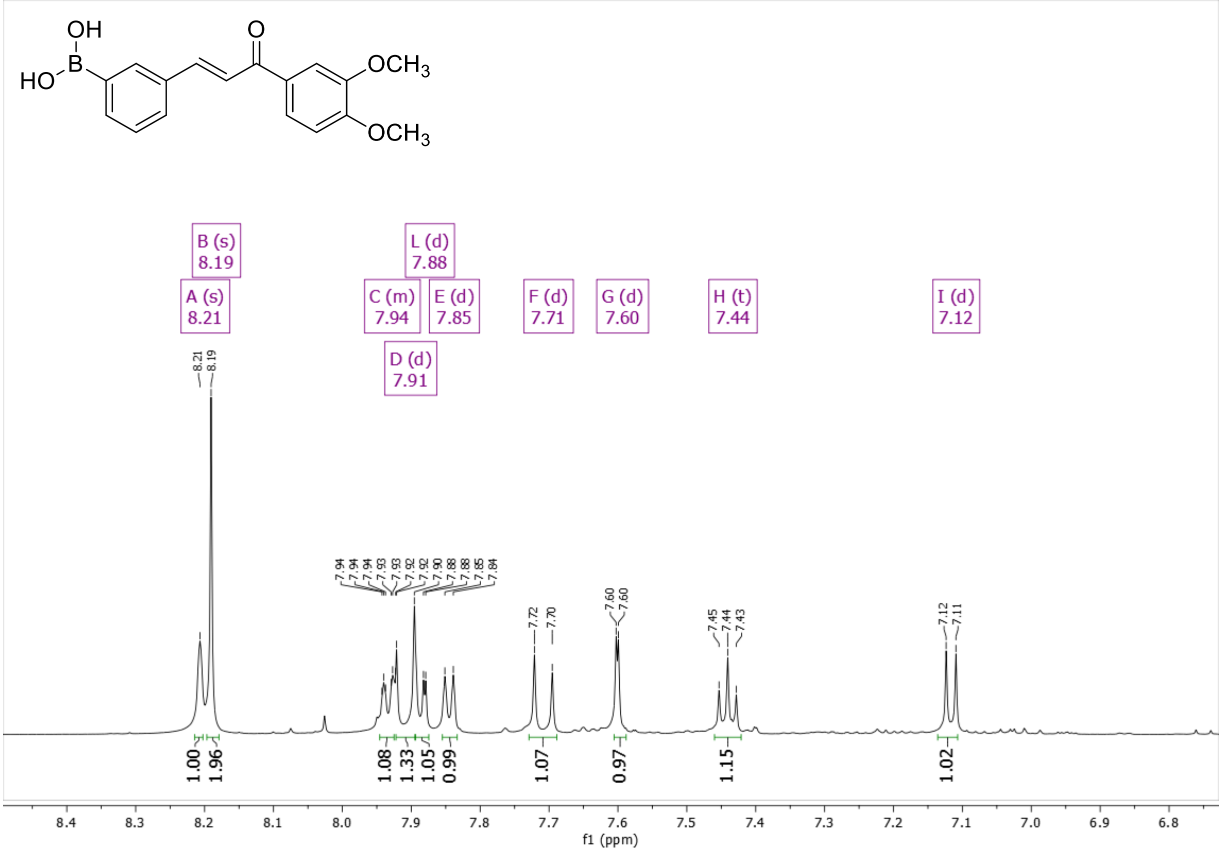

Figure S38. ^{1}H-NMR Spectrum of compound **6** (600 MHz DMSO-*d6*).

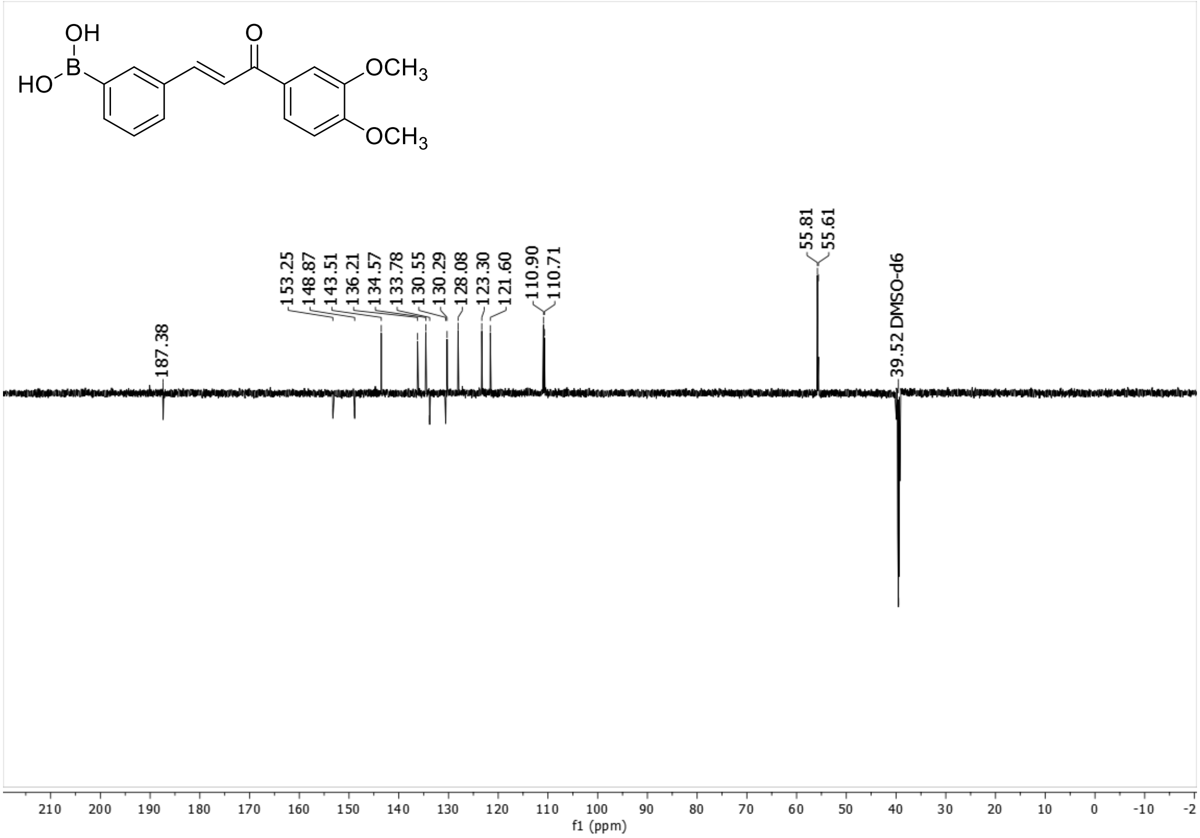

Figure S39. DEPTQ-NMR Spectrum of compound **6** (150 MHz DMSO-*d6*).

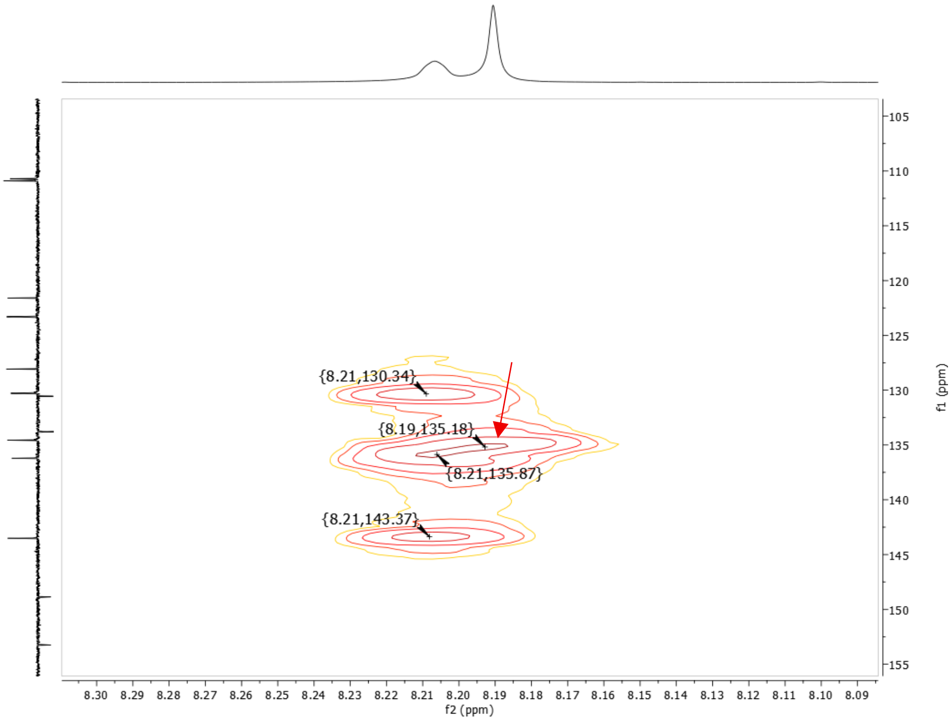

Figure S40. HMBC-NMR Spectrum of compound **6** (150 MHz DMSO-*d6*). The red arrow indicates the possible signal of the quaternary carbon directly bonded to boron (C–B).

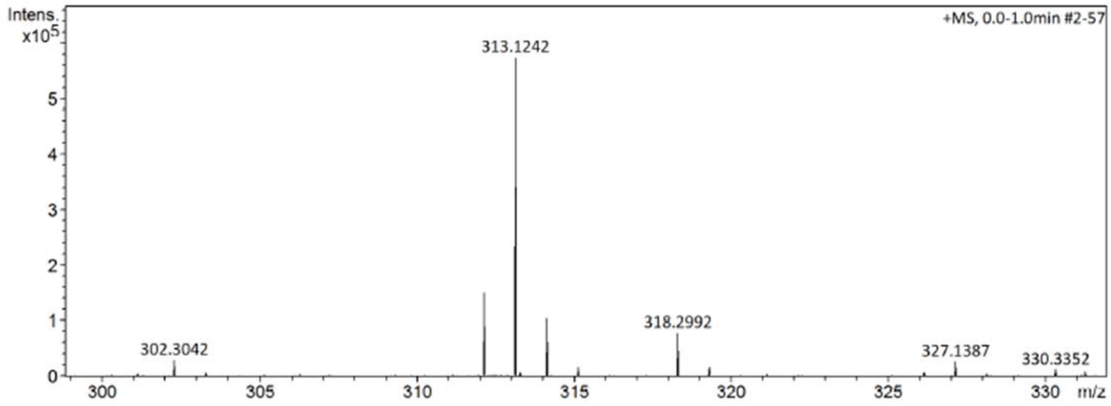

Figure S41. HRMS Spectrum of compound **6** (ESI+).

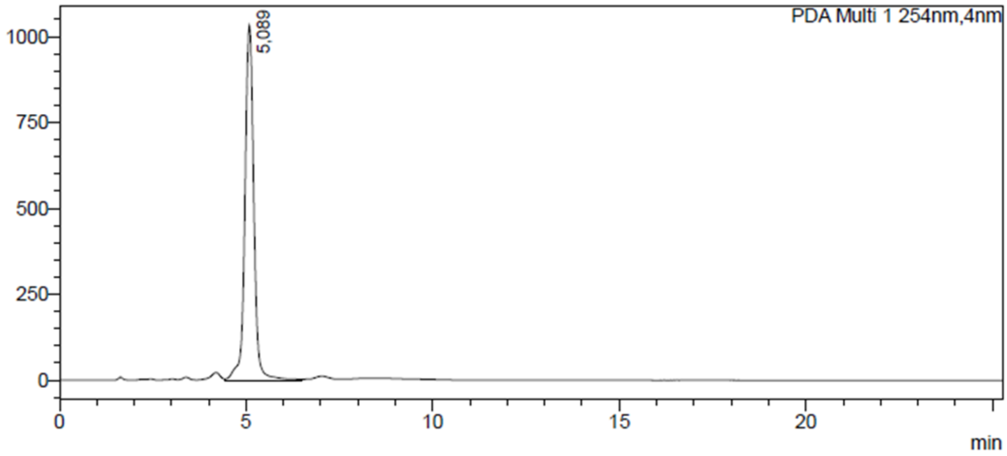

Figure S42. Chromatogram of compound **6**.

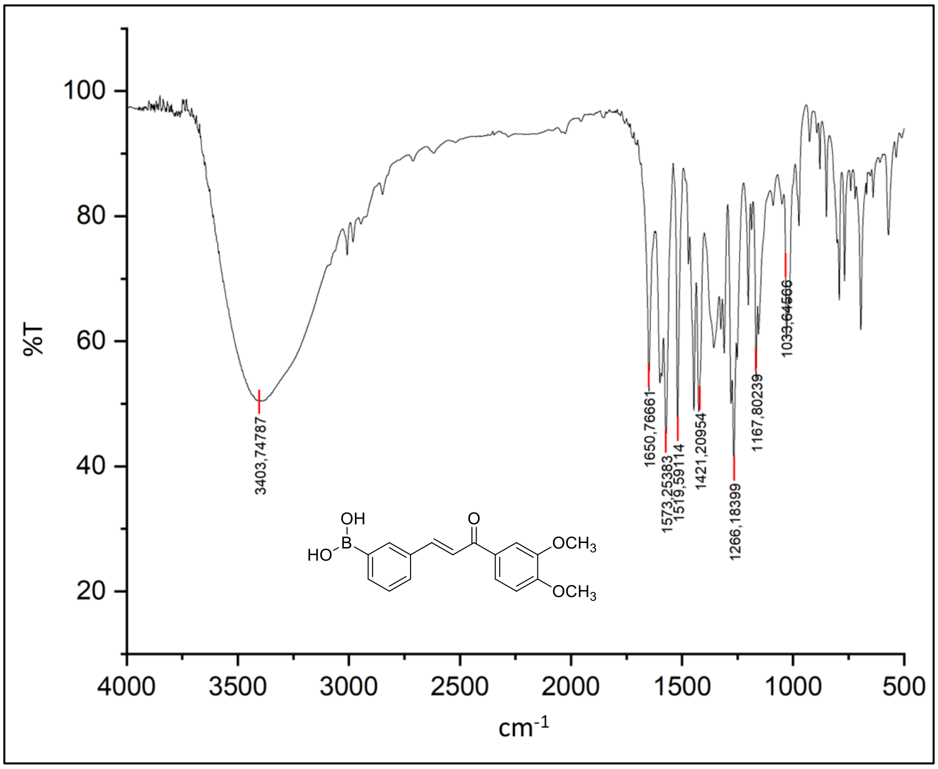

Figure S43. FTIR Spectrum of compound **6.**

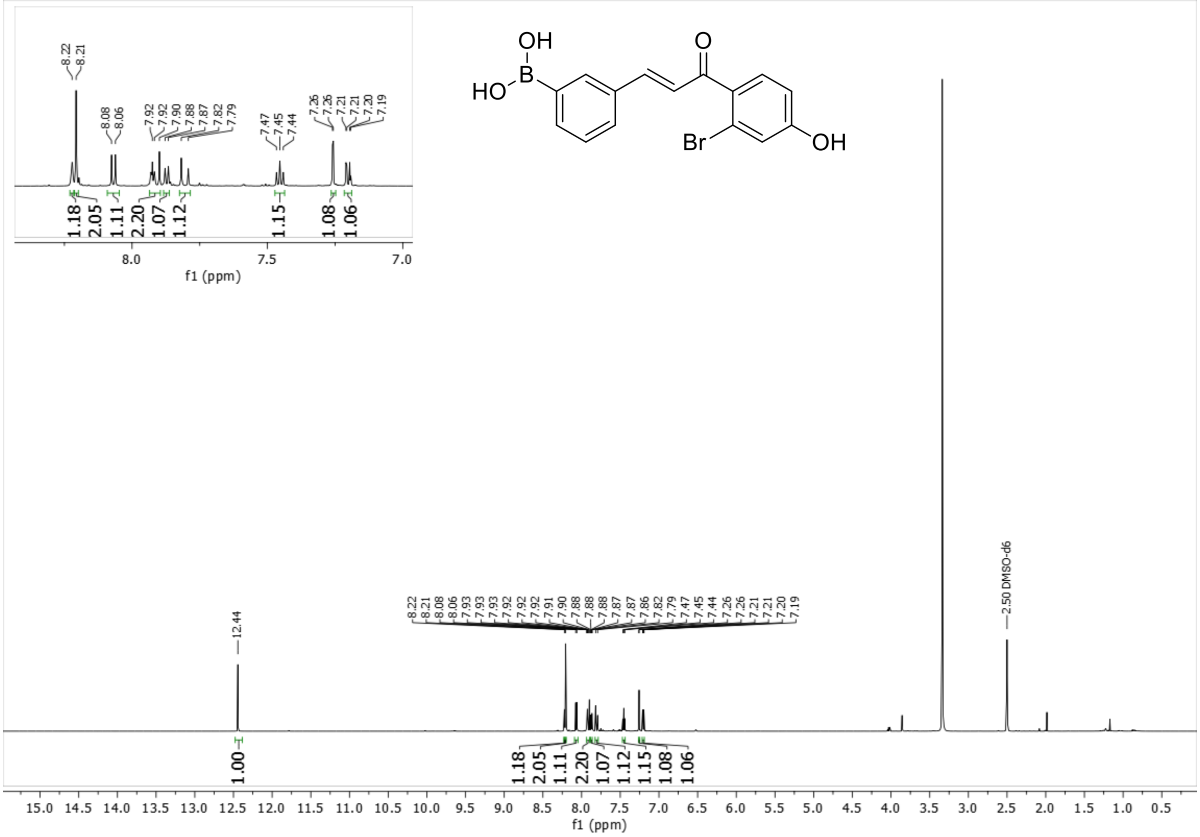

Figure S44. ^{1}H-NMR Spectrum of compound **7** (600 MHz DMSO-*d*6).

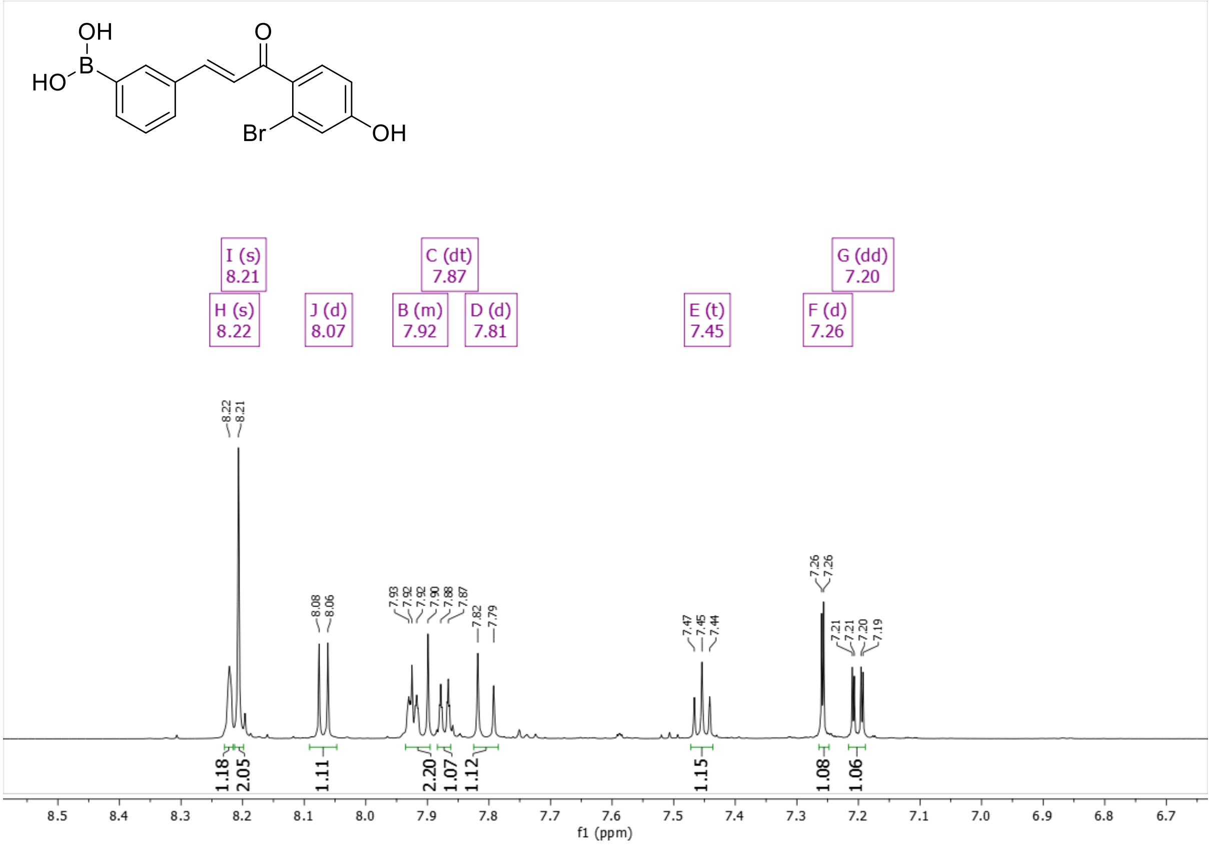

Figure S45. ^{1}H-NMR Spectrum of compound **7** (600 MHz DMSO-*d*6).

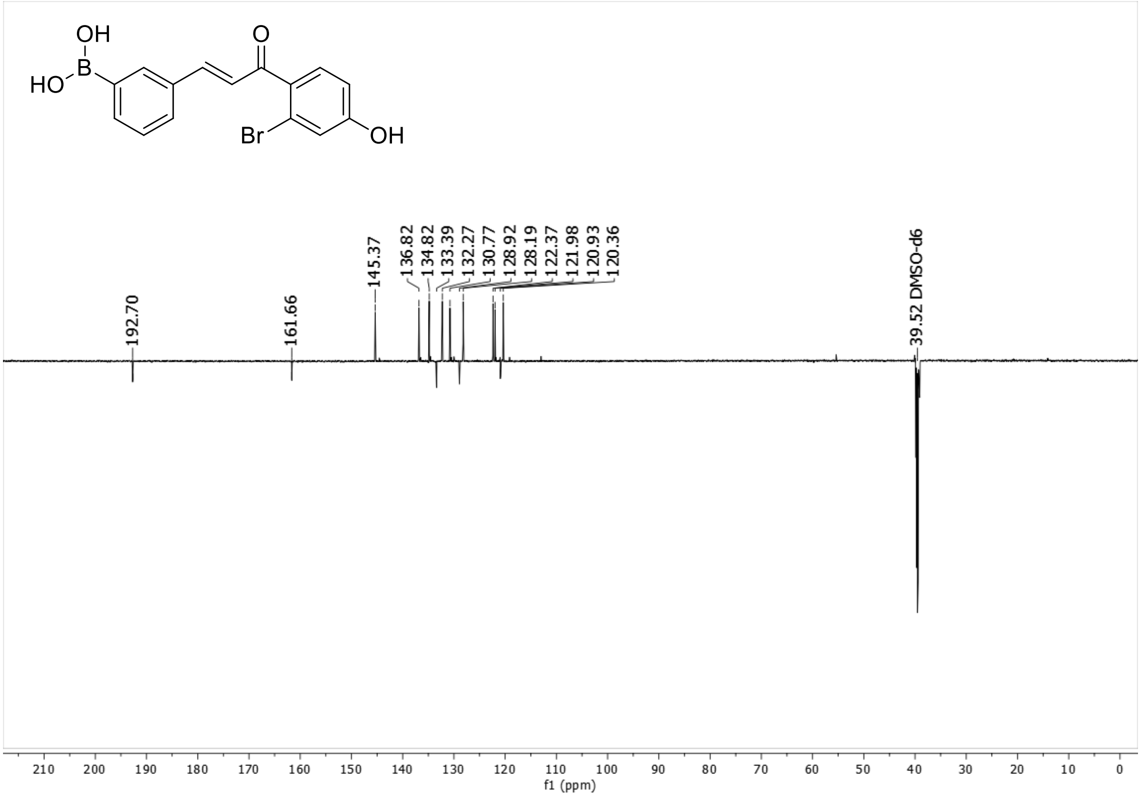

Figure S46. DEPTQ-NMR Spectrum of compound **7** (150 MHz DMSO-*d6*)

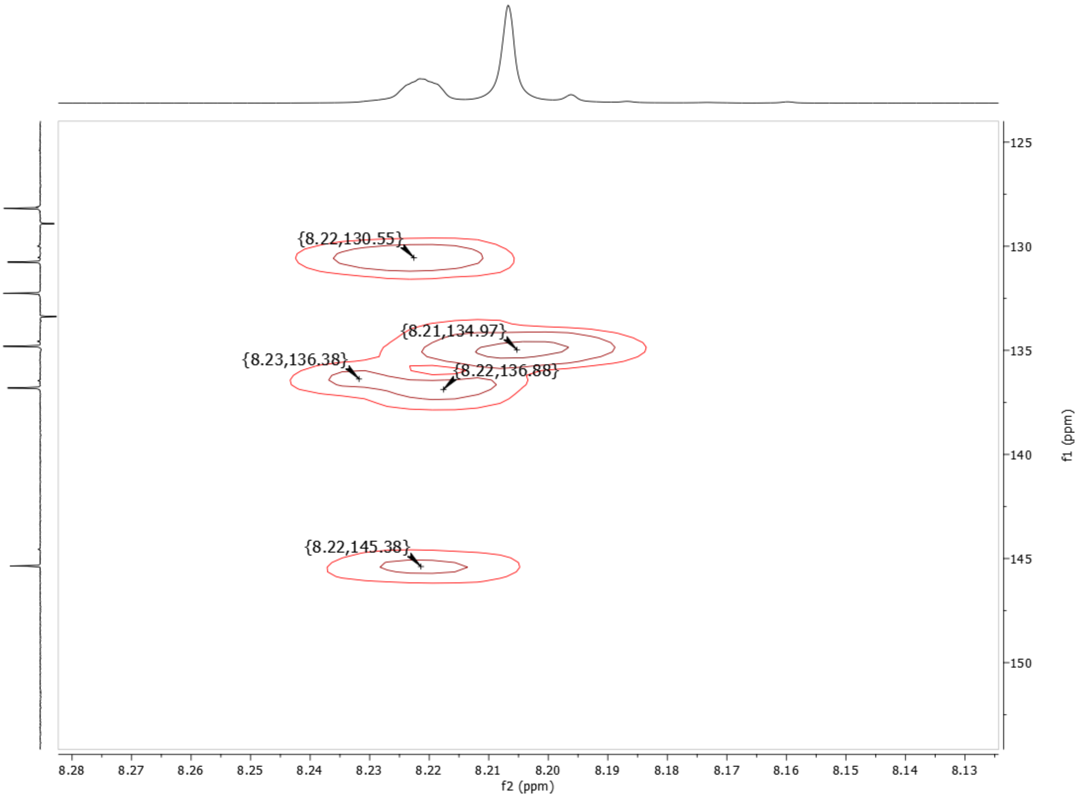

Figure S47. HMBC-NMR Spectrum of compound **7** (150 MHz DMSO-*d6*). The red arrow indicates the possible signal of the quaternary carbon directly bonded to boron (C–B).

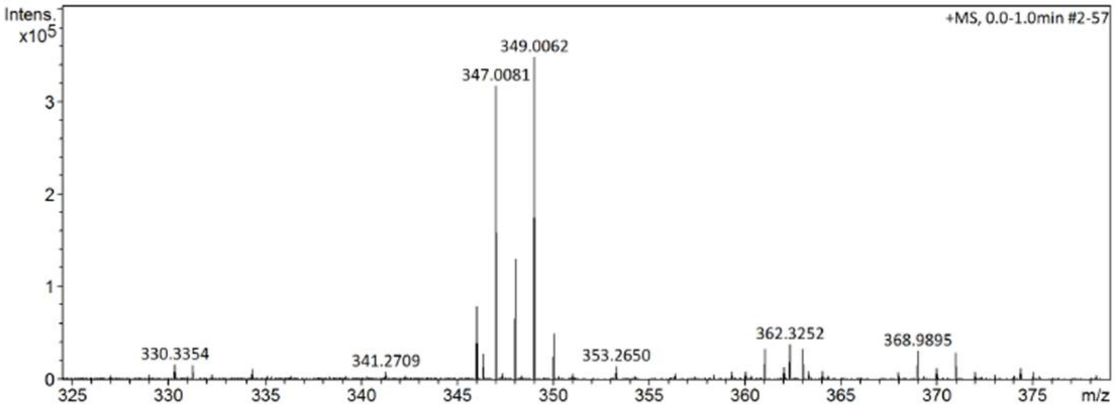

Figure S48. HRMS Spectrum of compound **7** (ESI+).

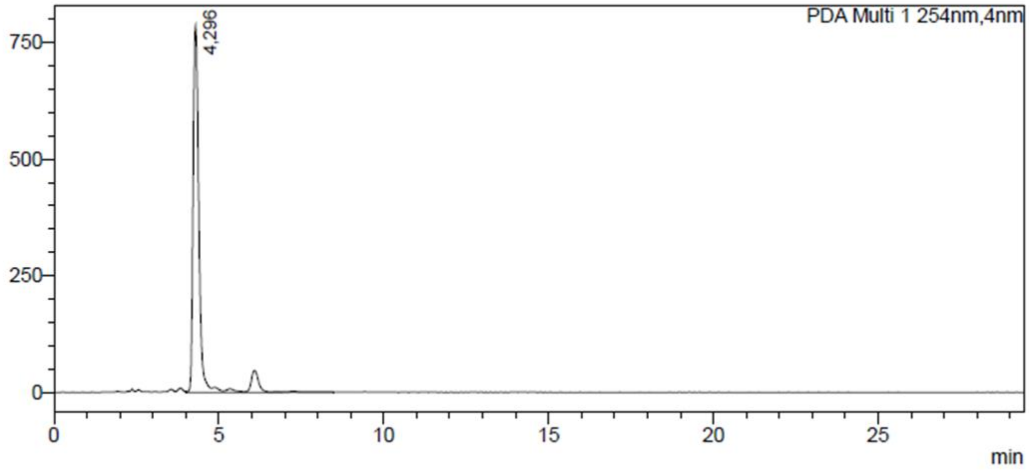

Figure S49. Chromatogram of compound **7**.

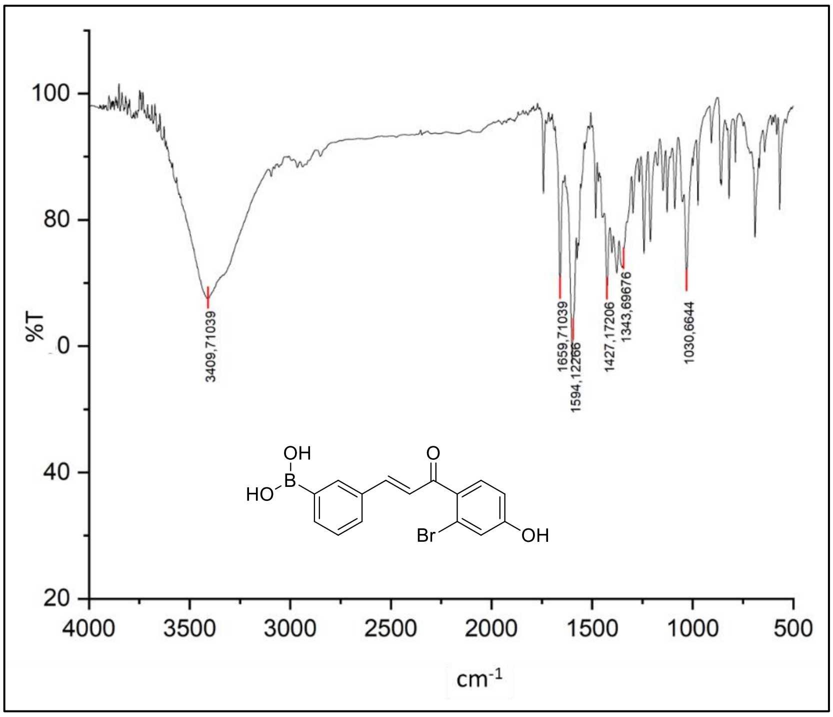

Figure S50. FTIR Spectrum of compound **7.**

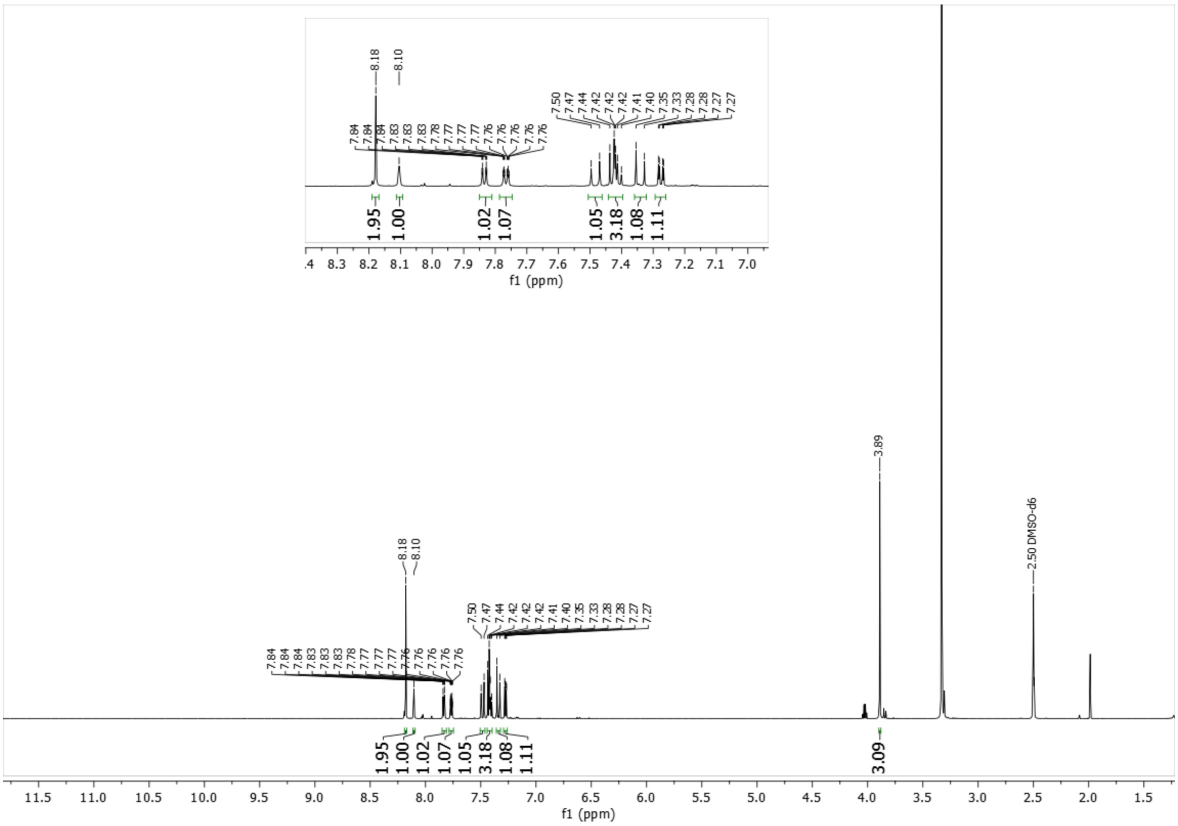

Figure S51. ^{1}H-NMR Spectrum of compound **8** (600 MHz DMSO-*d*6).

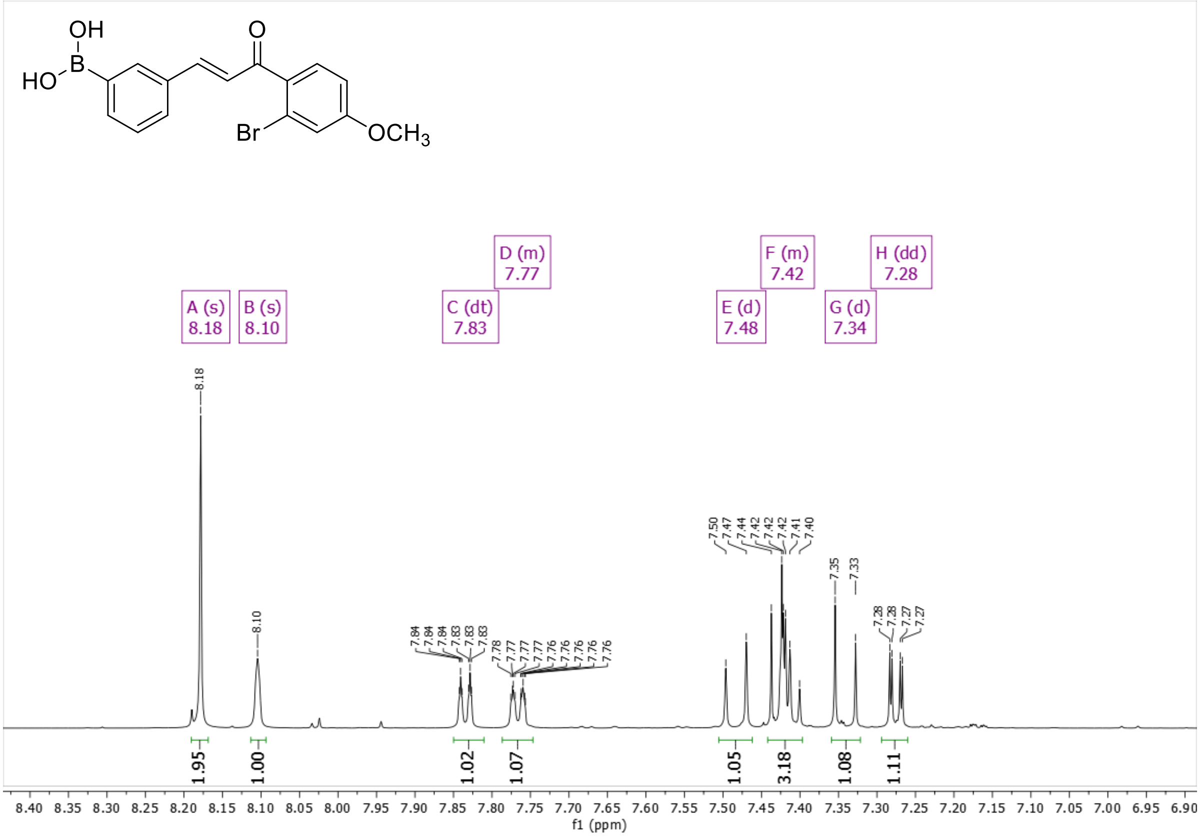

Figure S52. ^{1}H-NMR Spectrum of compound **8** (600 MHz DMSO-*d*6).

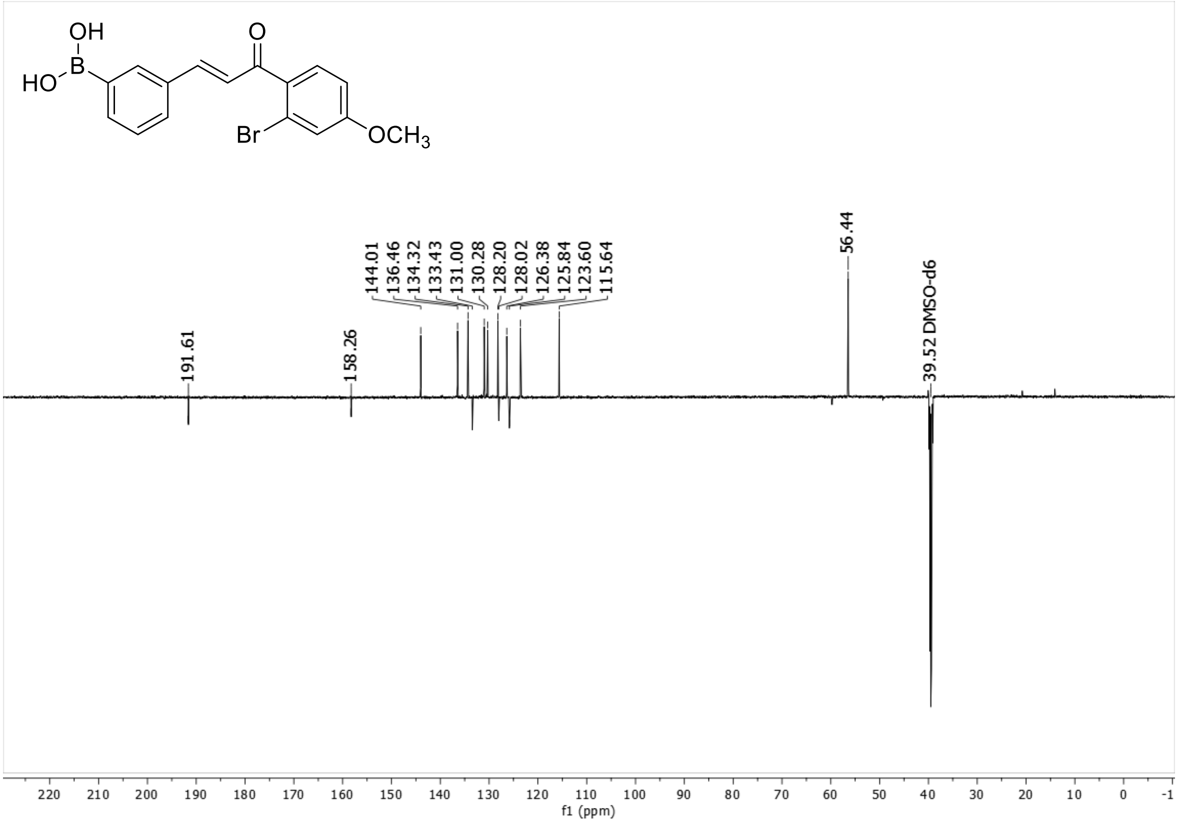

Figure S53. ^{13}C-NMR Spectrum of compound **8** (150 MHz DMSO-*d*6).

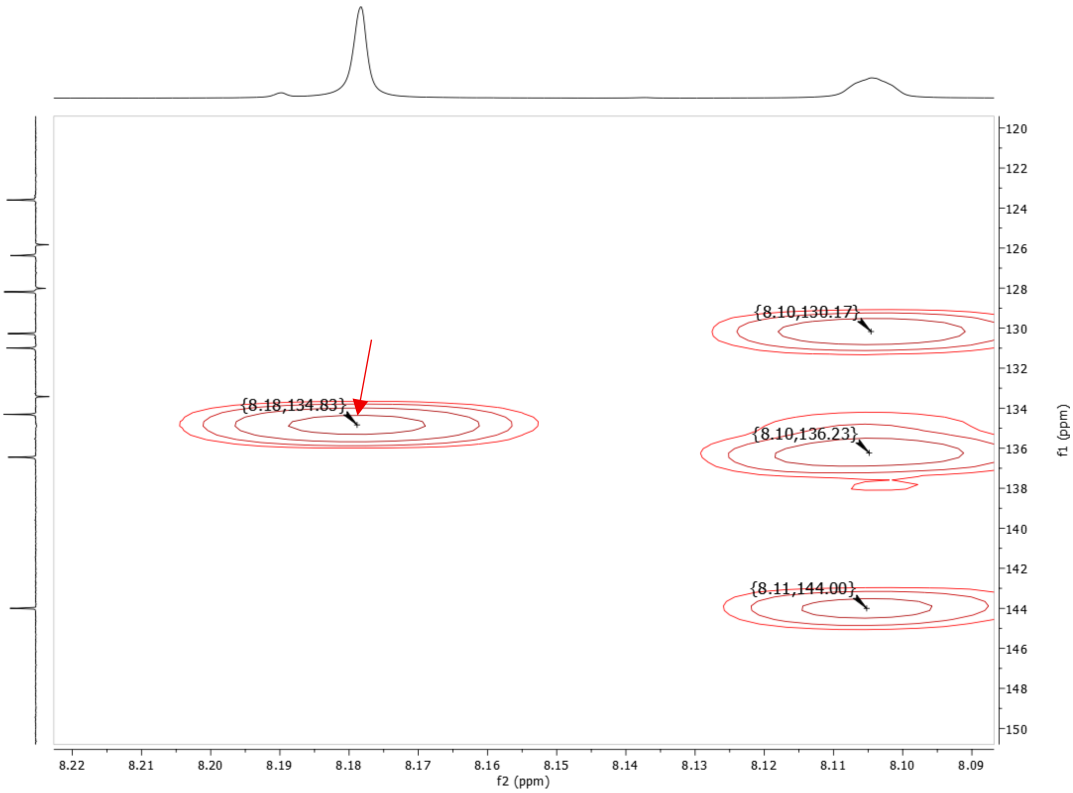

Figure S54. HMBC-NMR Spectrum of compound **8** (150 MHz DMSO-*d*6). The red arrow indicates the possible signal of the quaternary carbon directly bonded to boron (C–B).

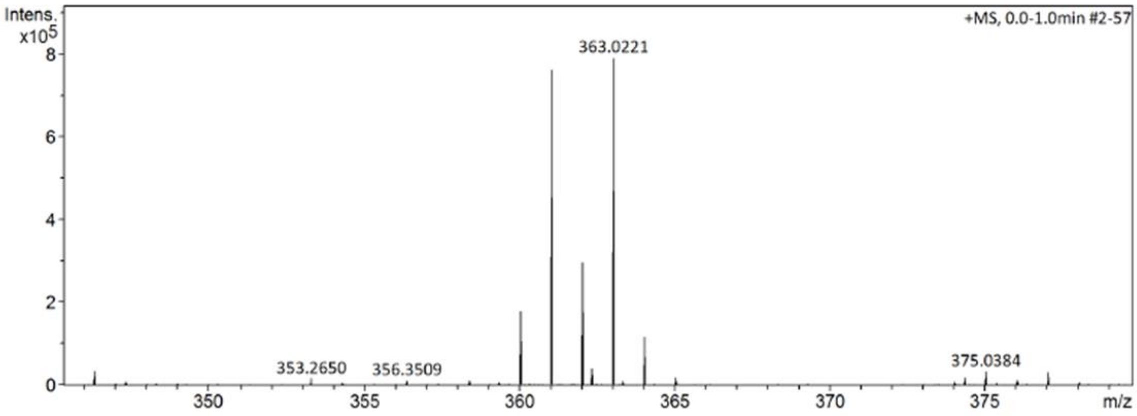

Figure S55. HRMS Spectrum of compound **8** (ESI+).

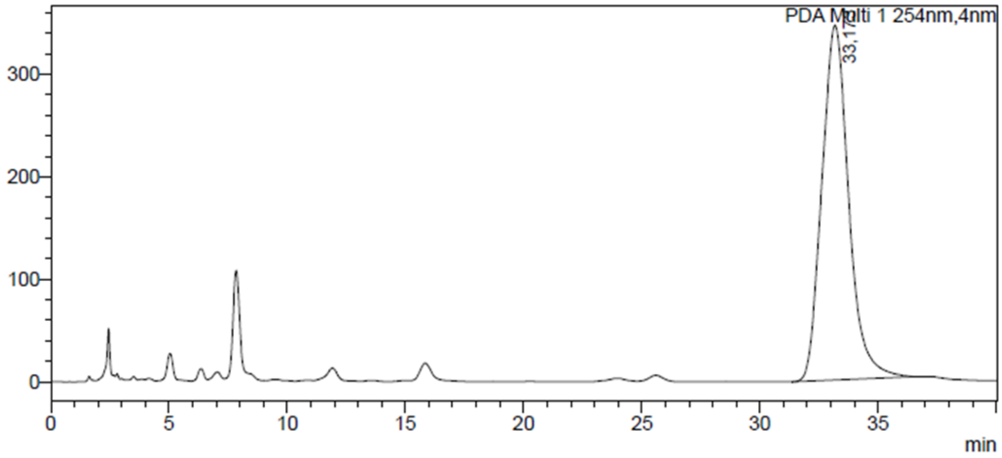

Figure S56. Chromatogram of compound **8**.

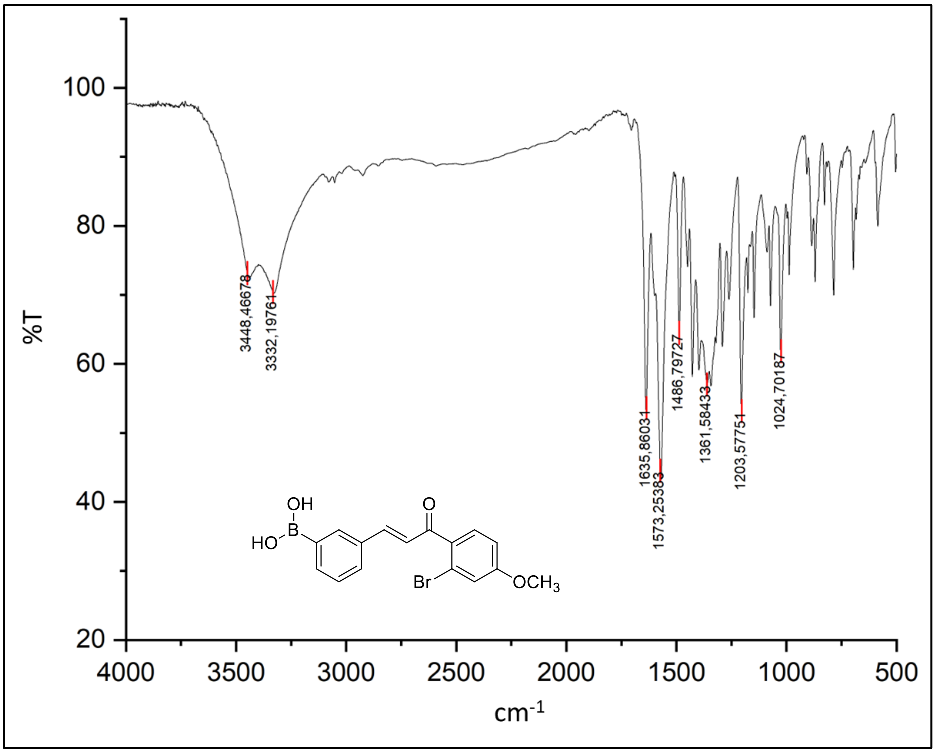

Figure S57. FTIR Spectrum of compound **8.**

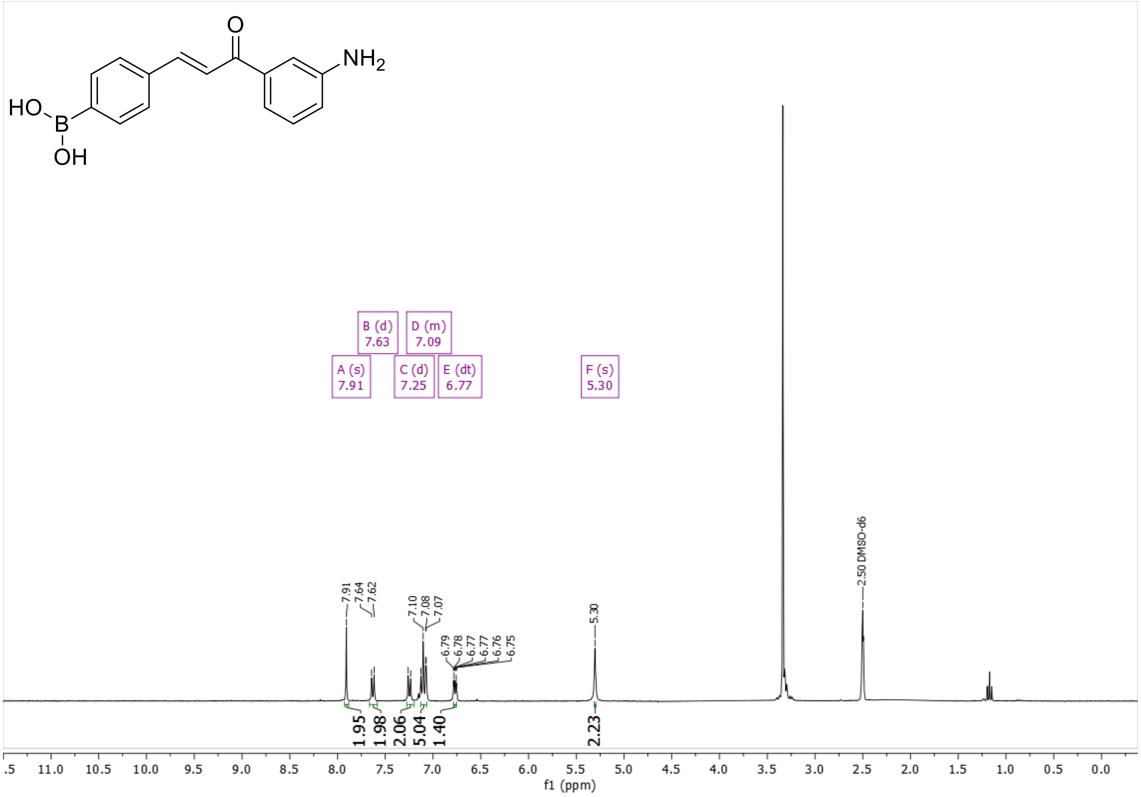

Figure S58. ^{1}H-NMR Spectrum of compound **9** (600 MHz DMSO-*d*6).

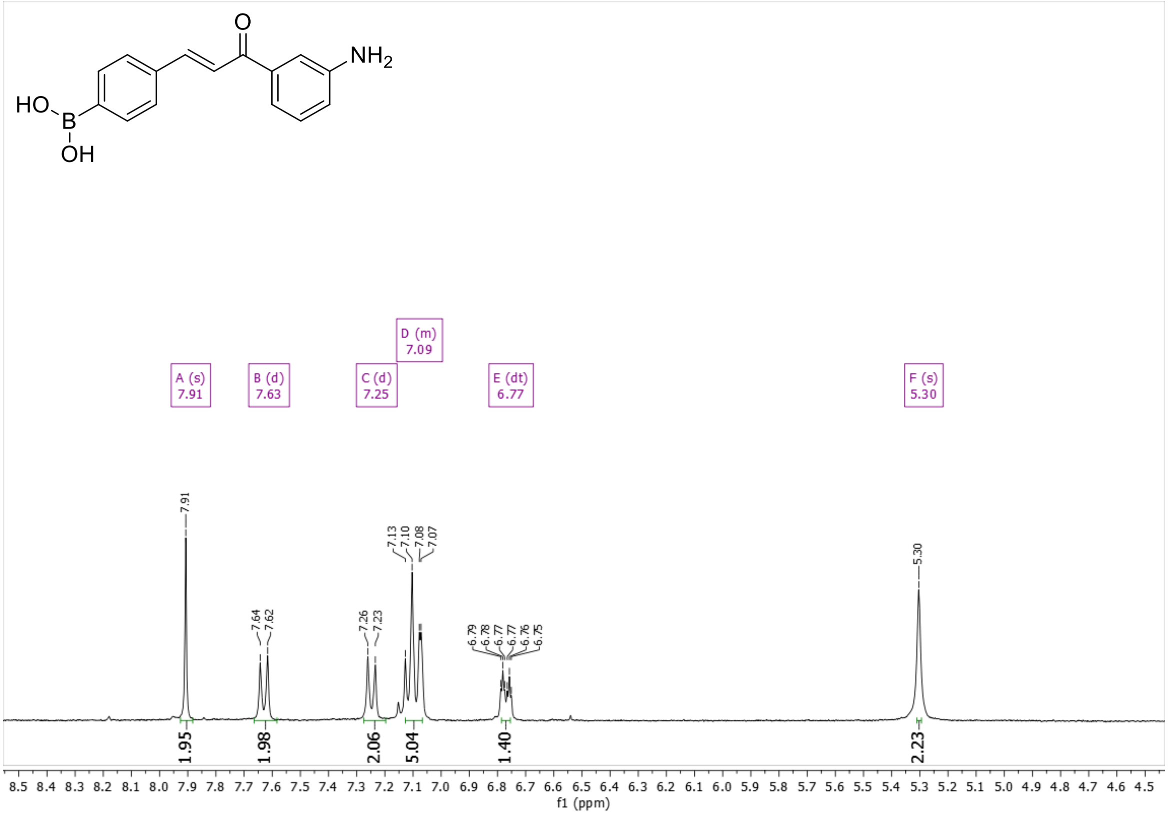

Figure S59. ^{1}H-NMR Spectrum of compound **9** (600 MHz DMSO-*d*6).

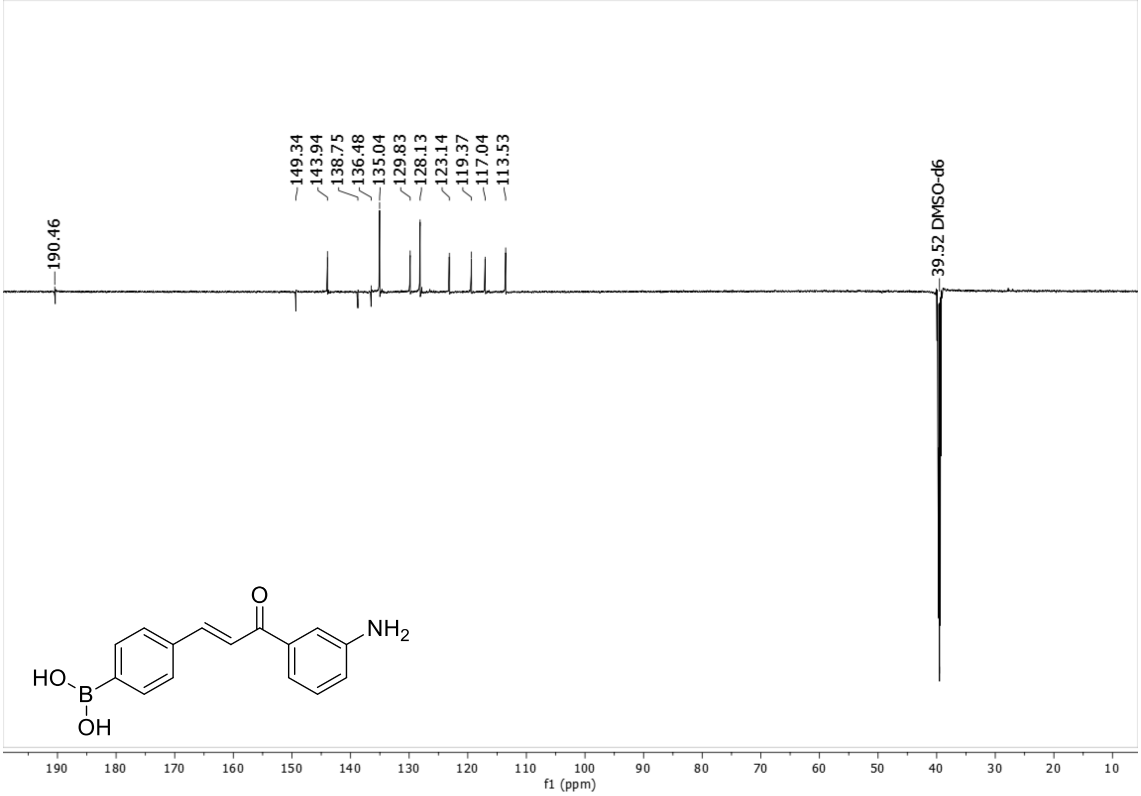

Figure S60. DEPTQ-NMR Spectrum of compound **9** (150 MHz DMSO-*d*6).

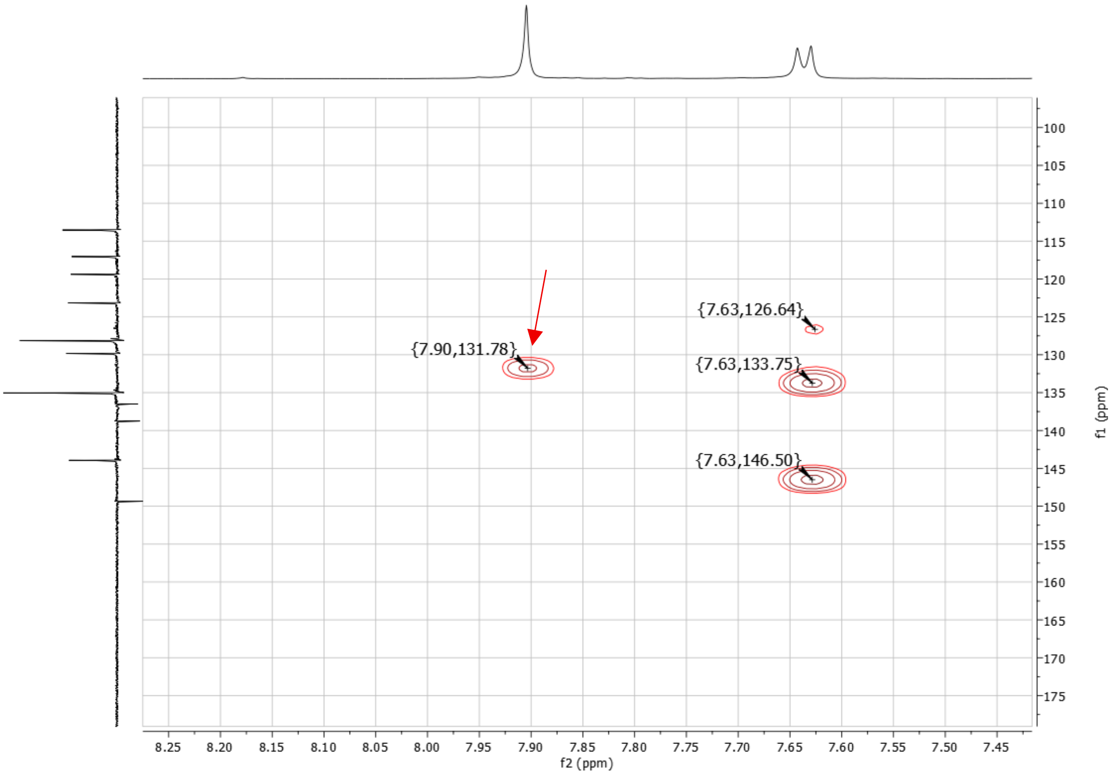

Figure S61. HMBC-NMR Spectrum of compound **9** (150 MHz DMSO-*d*6). The red arrow indicates the possible signal of the quaternary carbon directly bonded to boron (C–B).

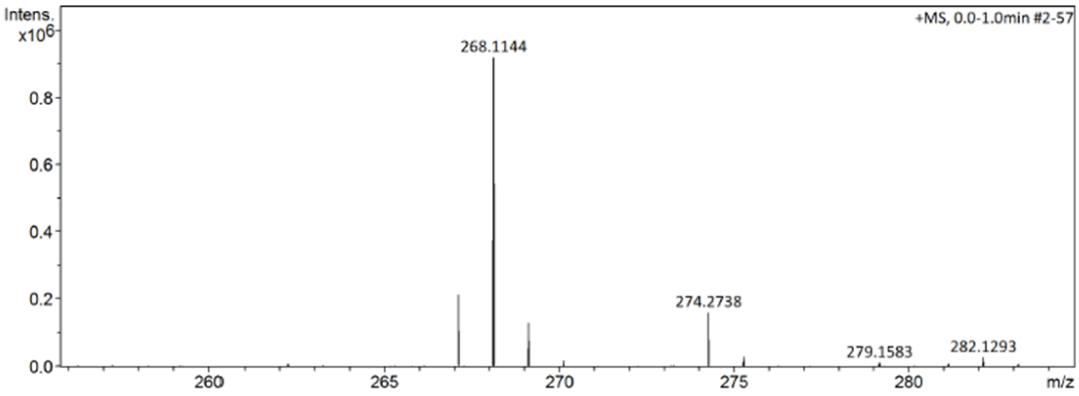

Figure S62. HRMS Spectrum of compound **9** (ESI+).

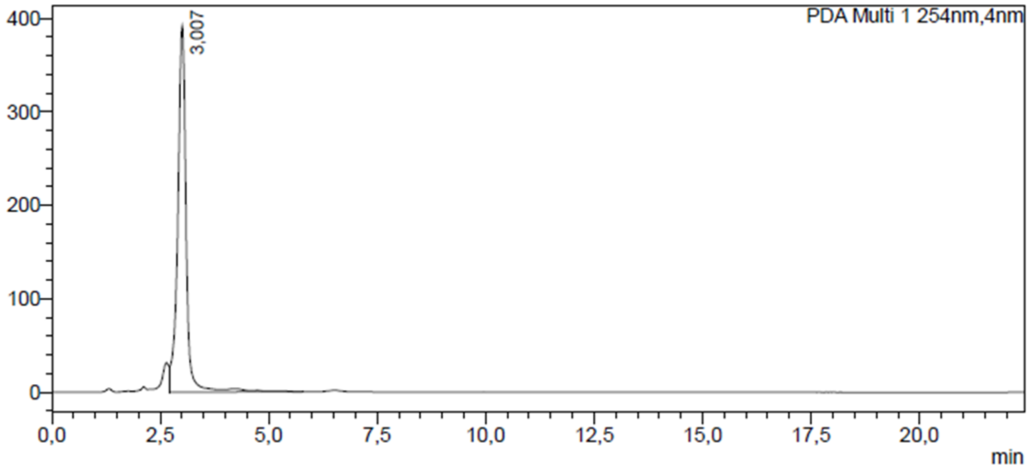

Figure S63. Chromatogram of compound **9**.

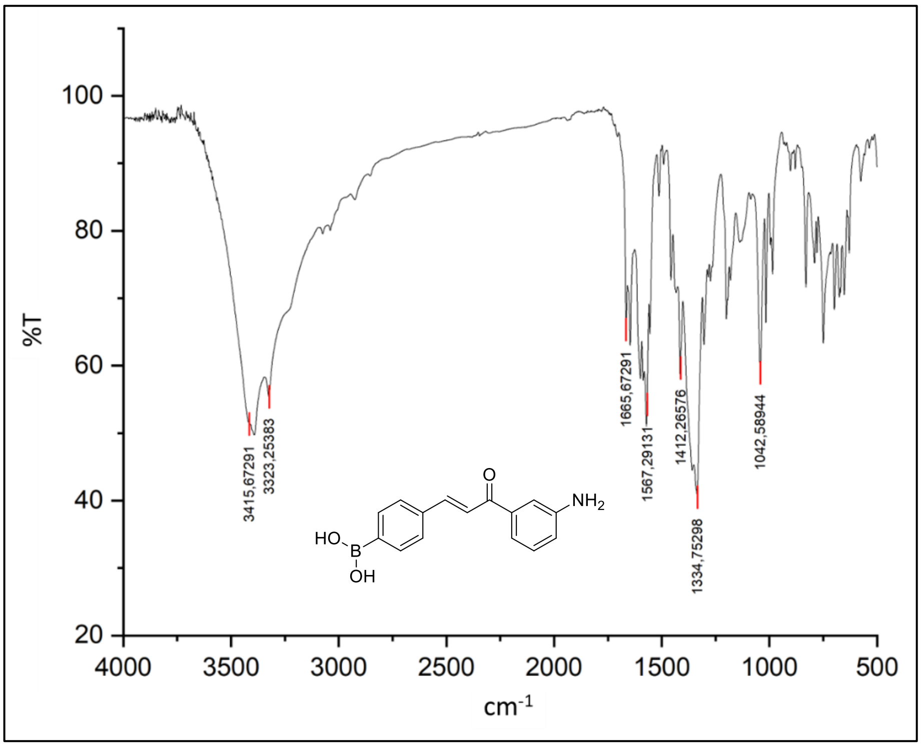

Figure S64. FTIR Spectrum of compound **9.**

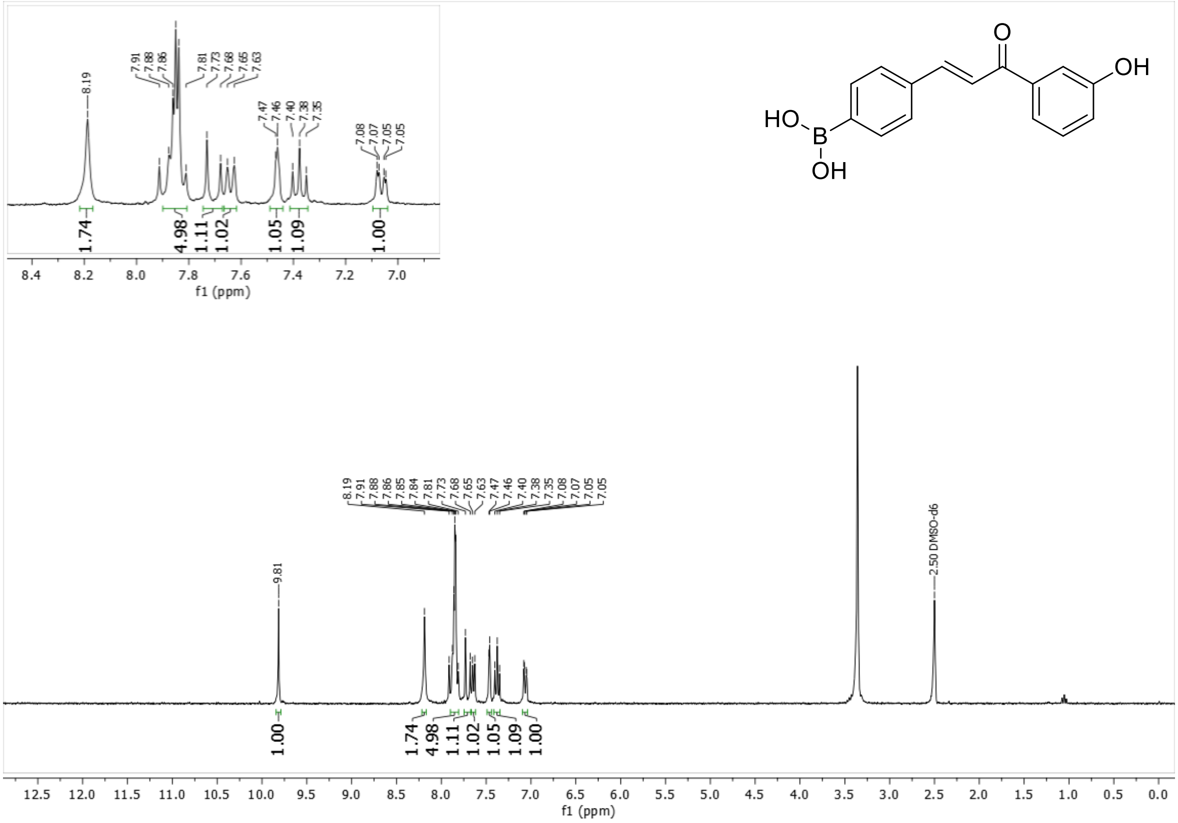

Figure S65. ^{1}H-NMR Spectrum of compound **10** (600 MHz DMSO-*d*6).

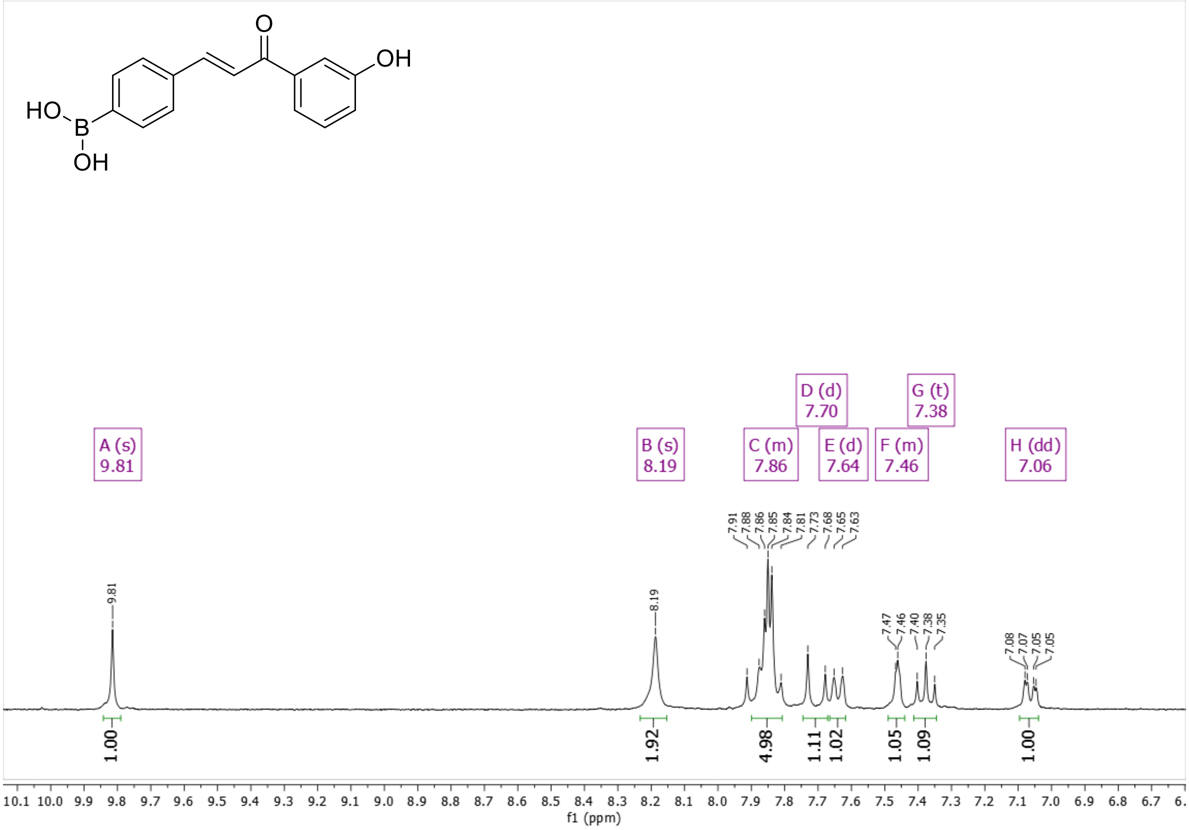

Figure S66. ^{1}H-NMR Spectrum of compound **10** (600 MHz DMSO-*d*6).

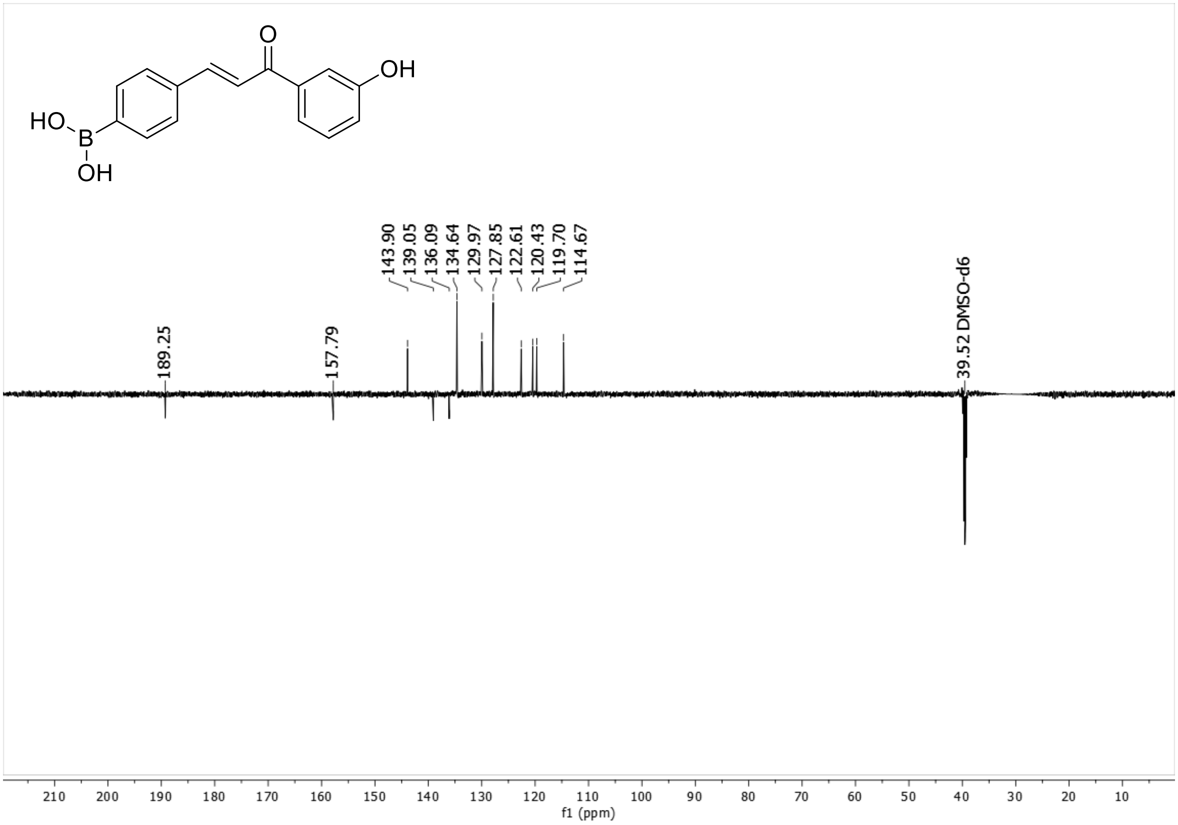

Figure S67. ^{13}C-NMR Spectrum of compound **10** (150 MHz DMSO-*d6*).

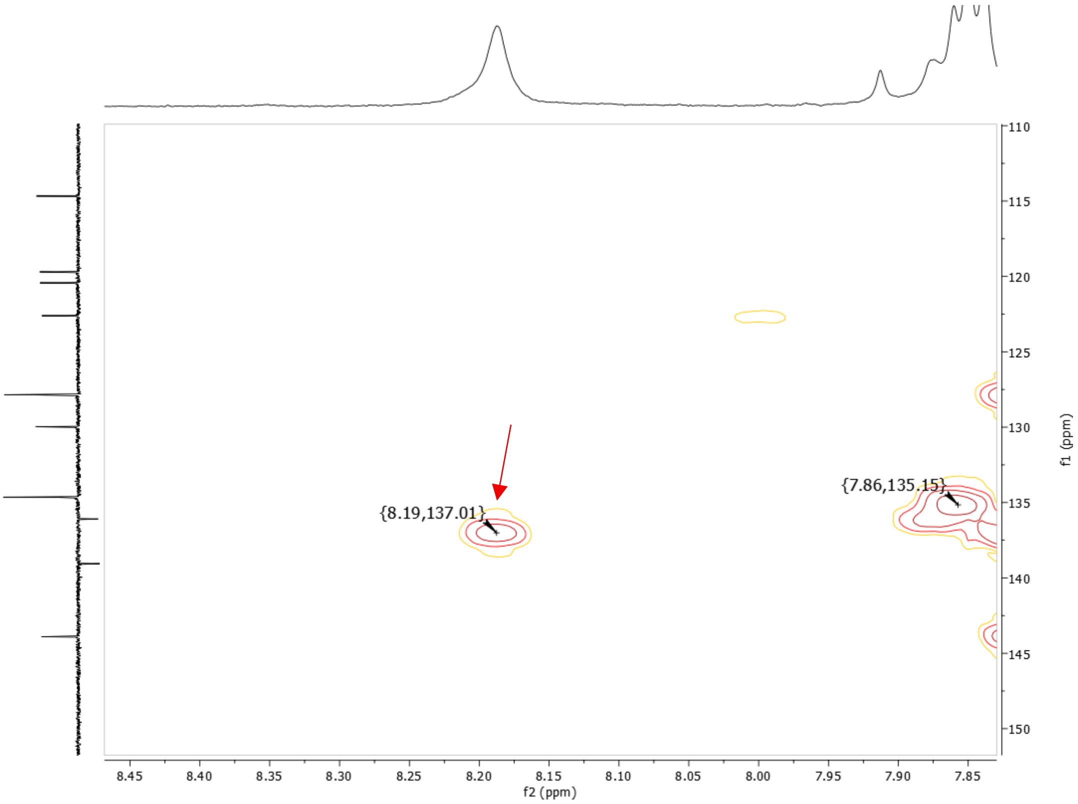

Figure S68. HMBC-NMR Spectrum of compound **10** (150 MHz DMSO-*d6*). The red arrow indicates the possible signal of the quaternary carbon directly bonded to boron (C–B).

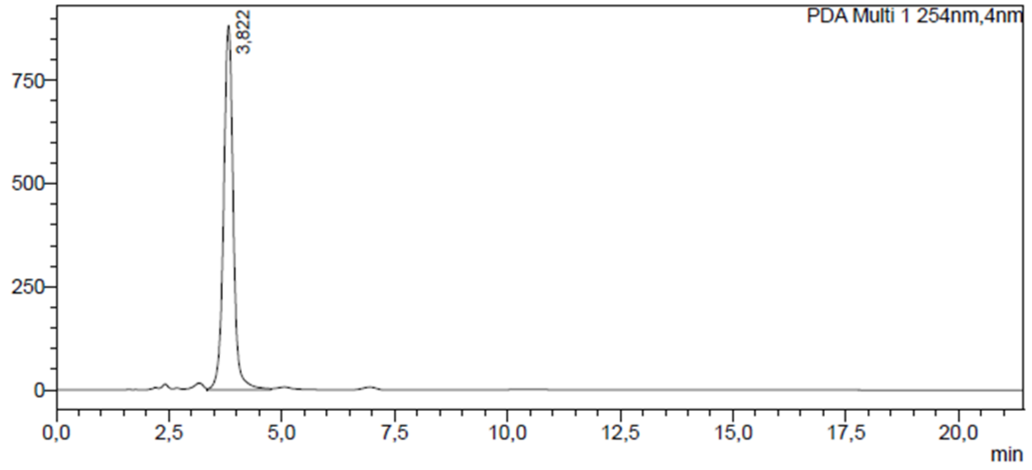

Figure S69. Chromatogram of compound **10**.

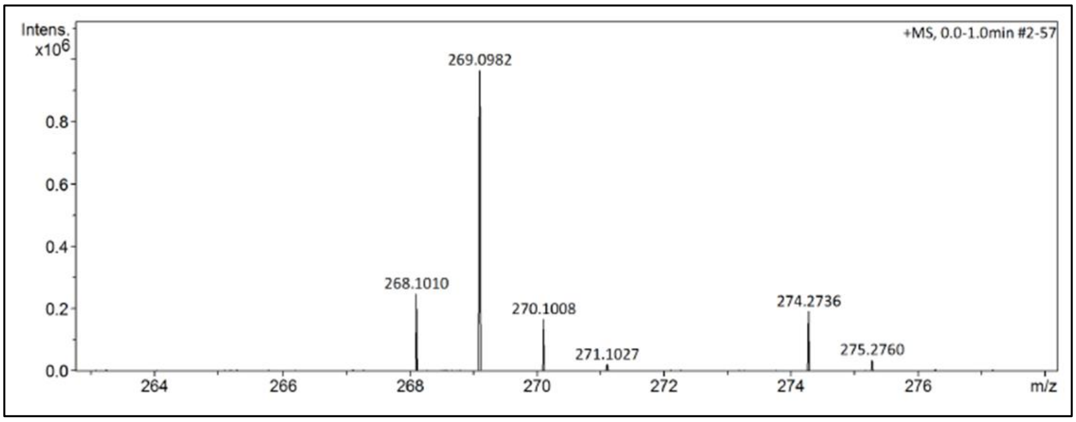

Figure S70. HRMS Spectrum of compound **10** (ESI+).

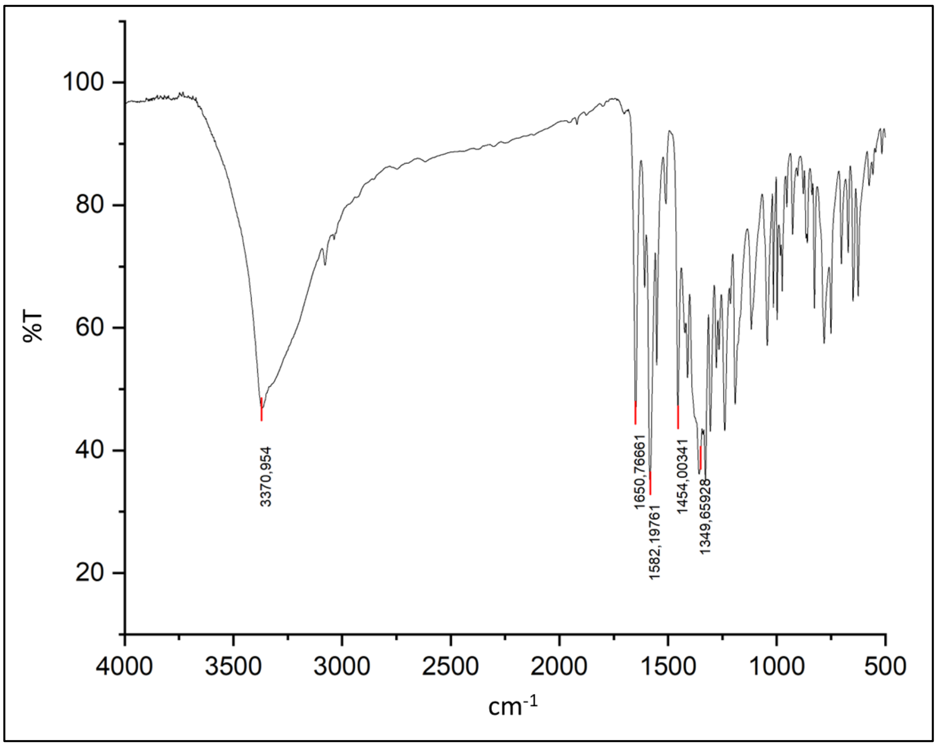

Figure S71. FTIR Spectrum of compound **10.**

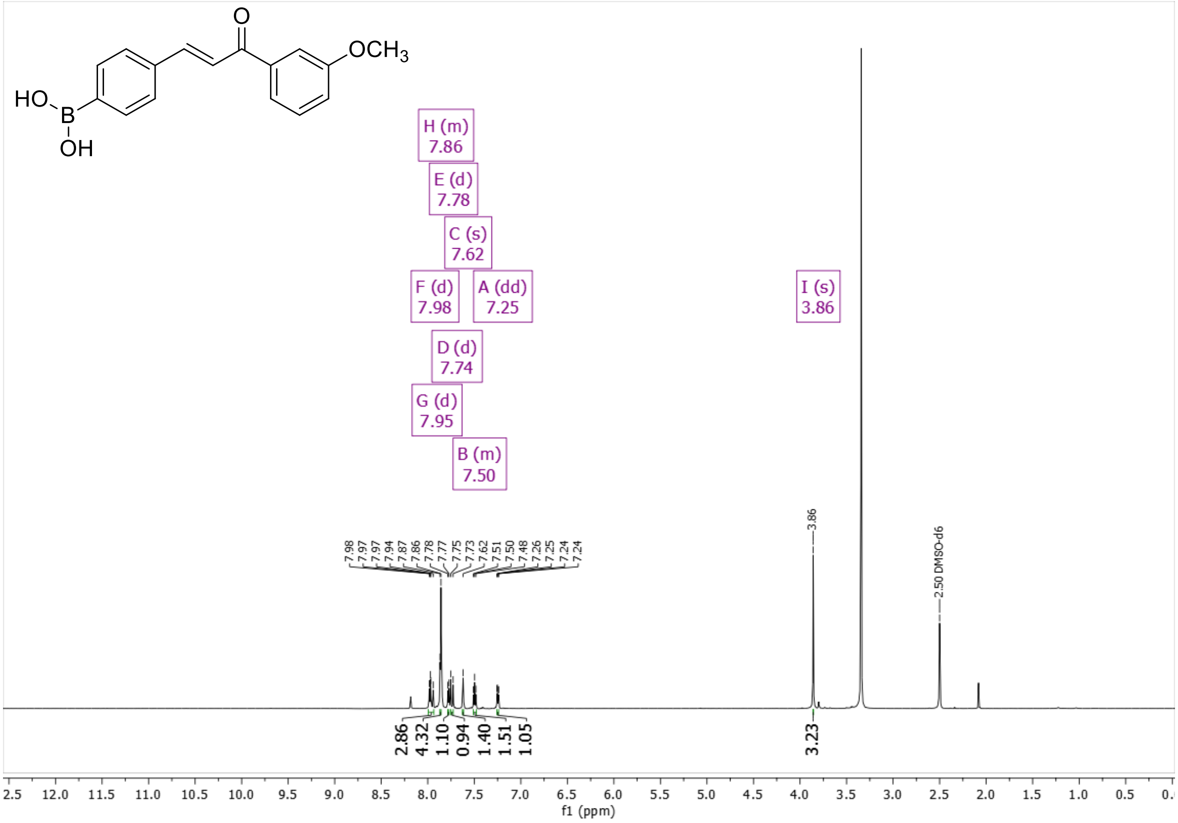

Figure S72. ^{1}H-NMR Spectrum of compound **11** (600 MHz DMSO-*d*6).

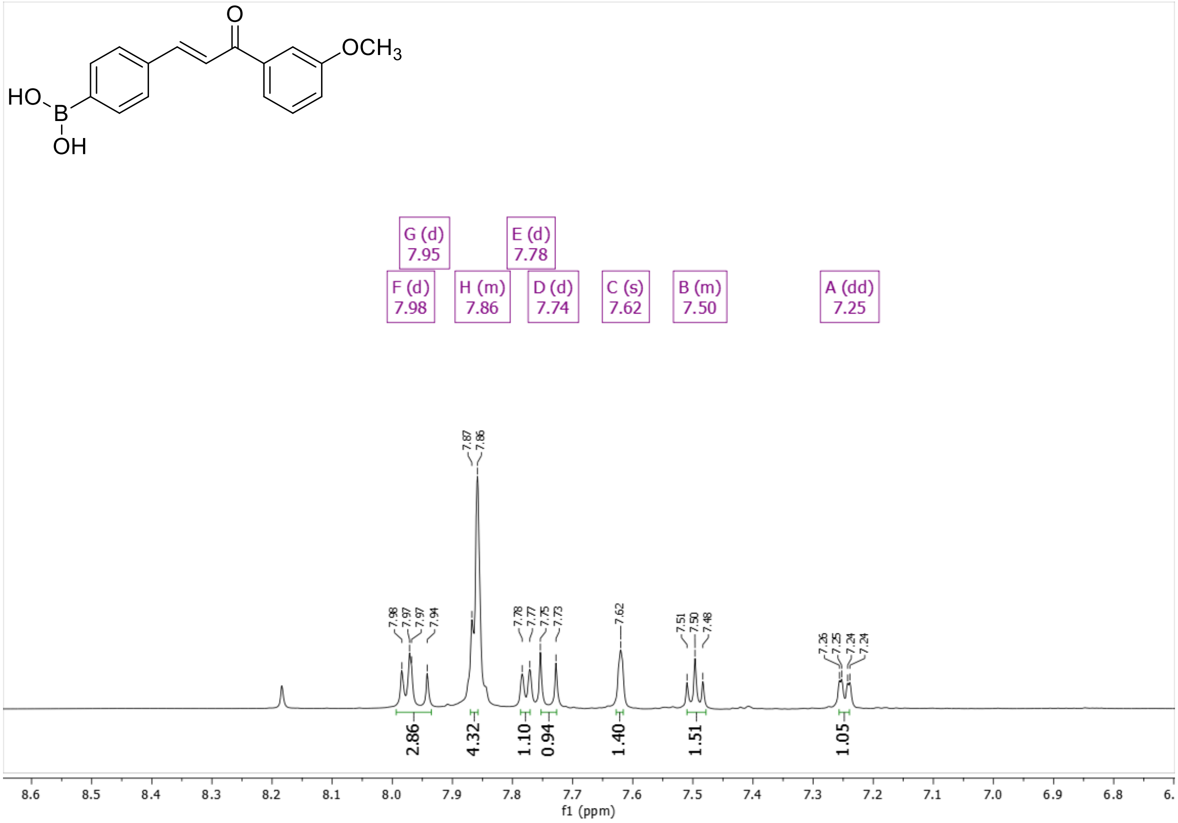

Figure S73. ^{1}H-NMR Spectrum of compound **11** (600 MHz DMSO-*d*6).

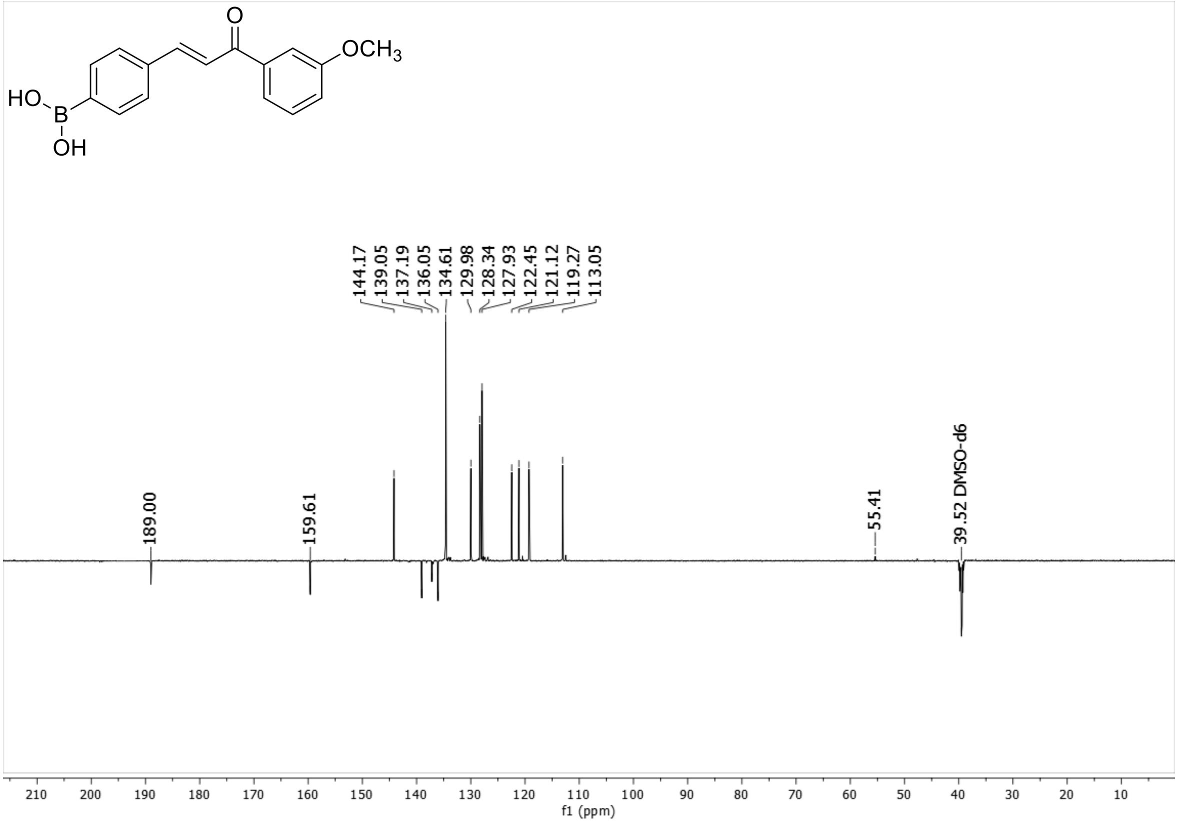

Figure S74. DEPTQ-NMR Spectrum of compound **11** (150 MHz DMSO-*d*6).

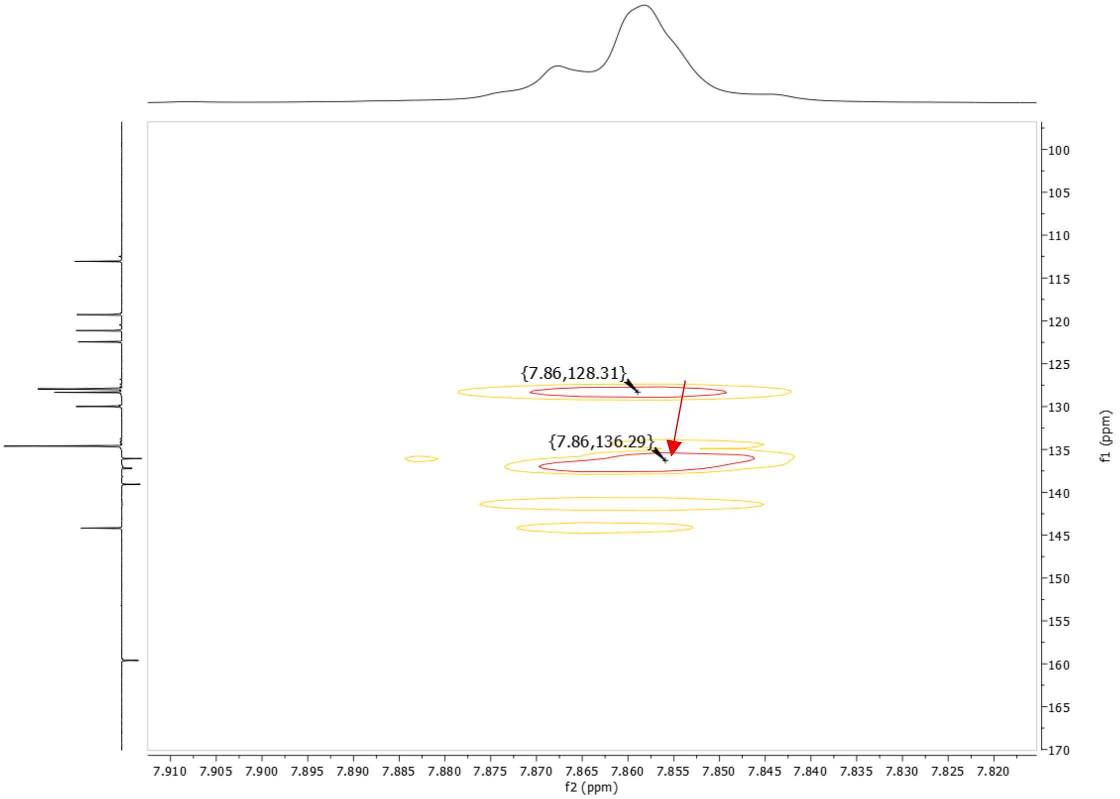

Figure S75. HMBC-NMR Spectrum of compound **11** (150 MHz DMSO-*d*6). The red arrow indicates the possible signal of the quaternary carbon directly bonded to boron (C–B).

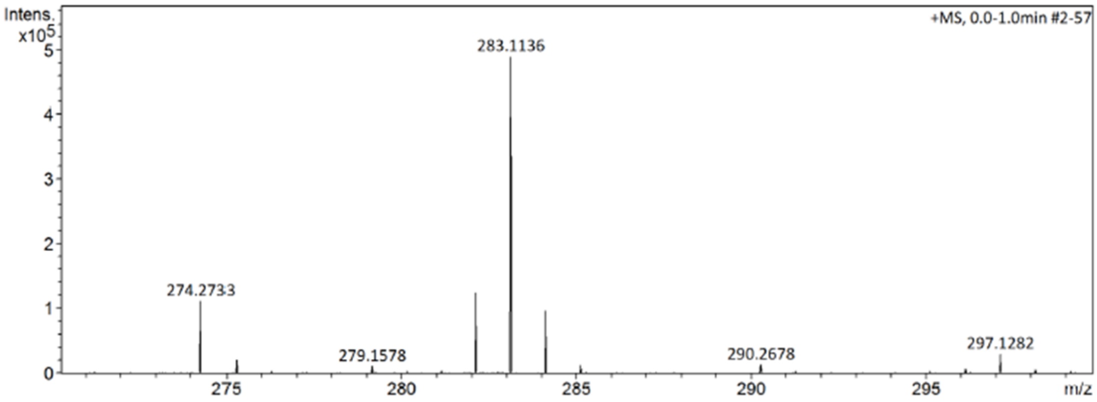

Figure S76. HRMS Spectrum of compound **11** (ESI+).

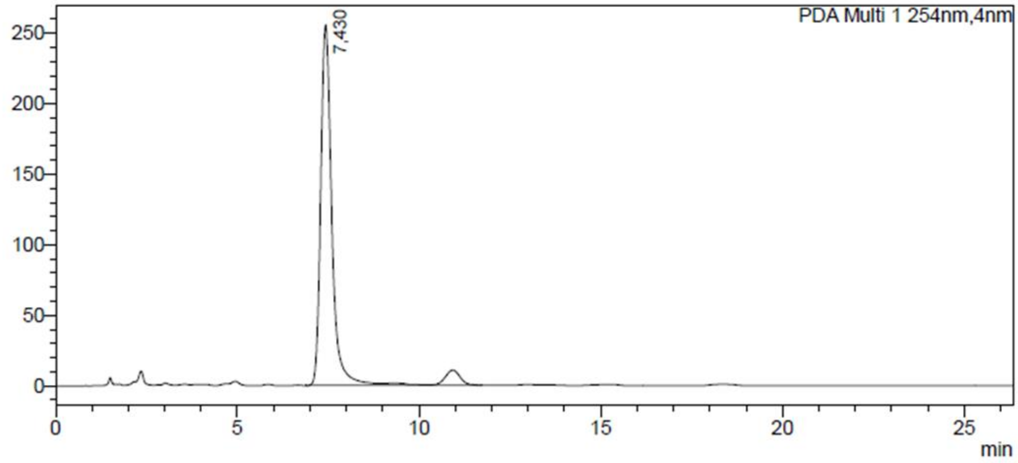

Figure S77. Chromatogram of compound **11**.

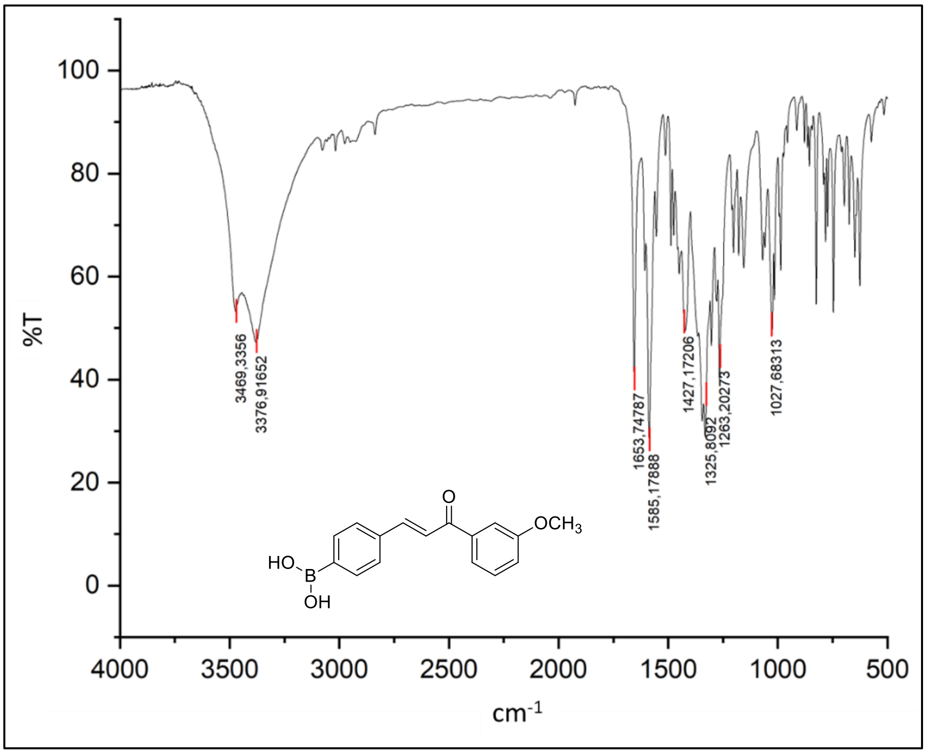

Figure S78. FTIR Spectrum of compound **11.**

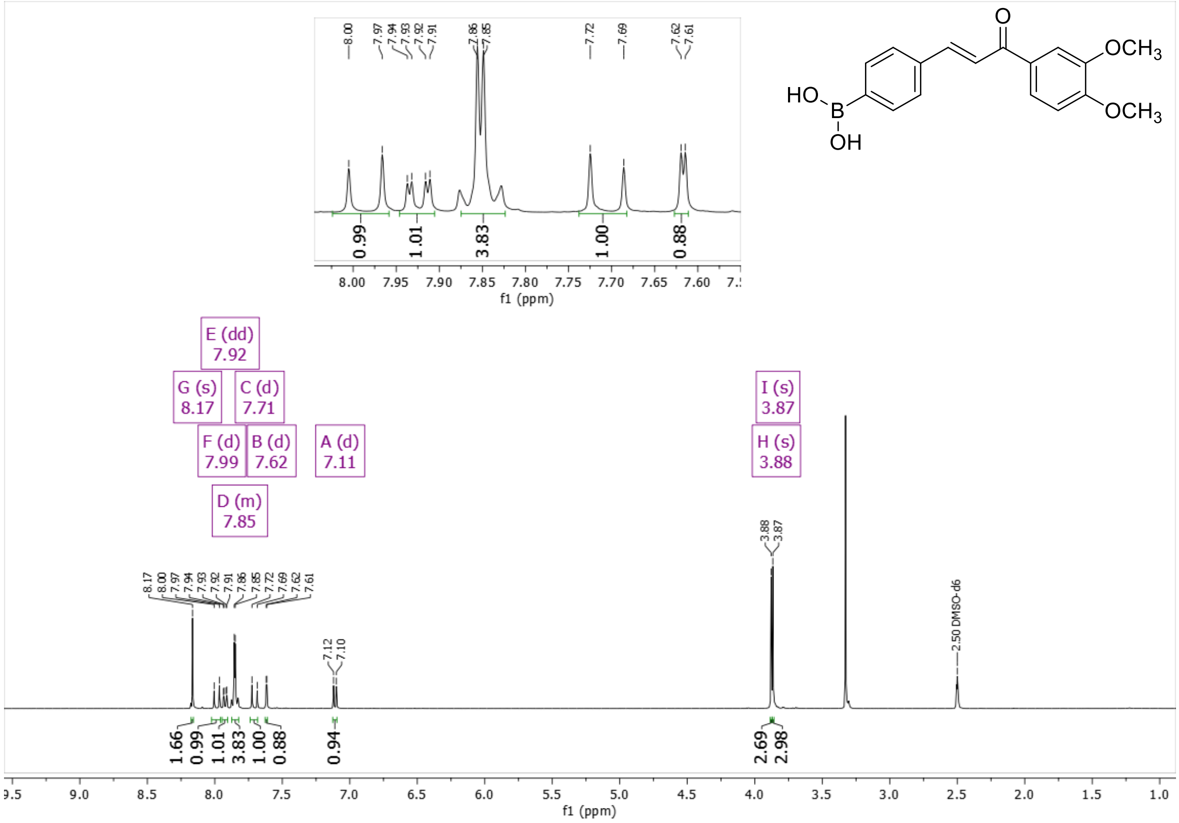

Figure S79. ^{1}H-NMR Spectrum of compound **12** (600 MHz DMSO-*d*6).

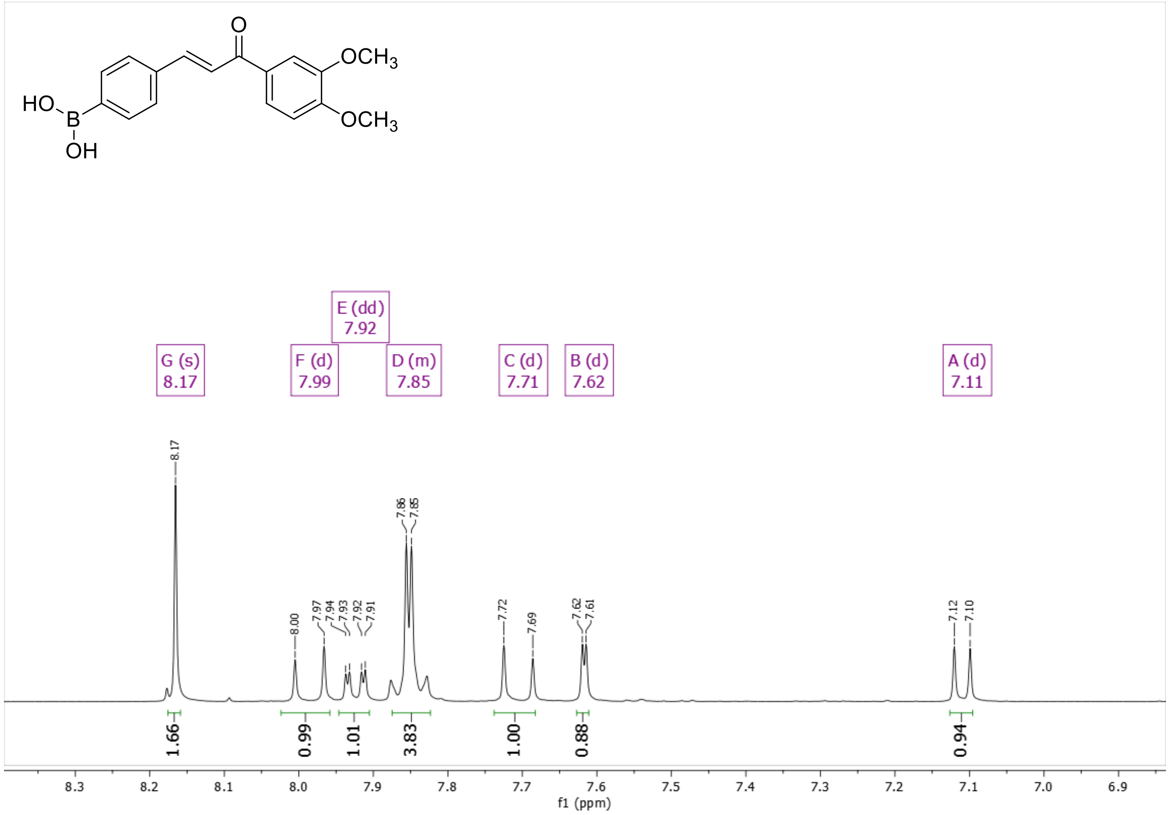

Figure S80. ^{1}H-NMR Spectrum of compound **12** (600 MHz DMSO-*d*6).

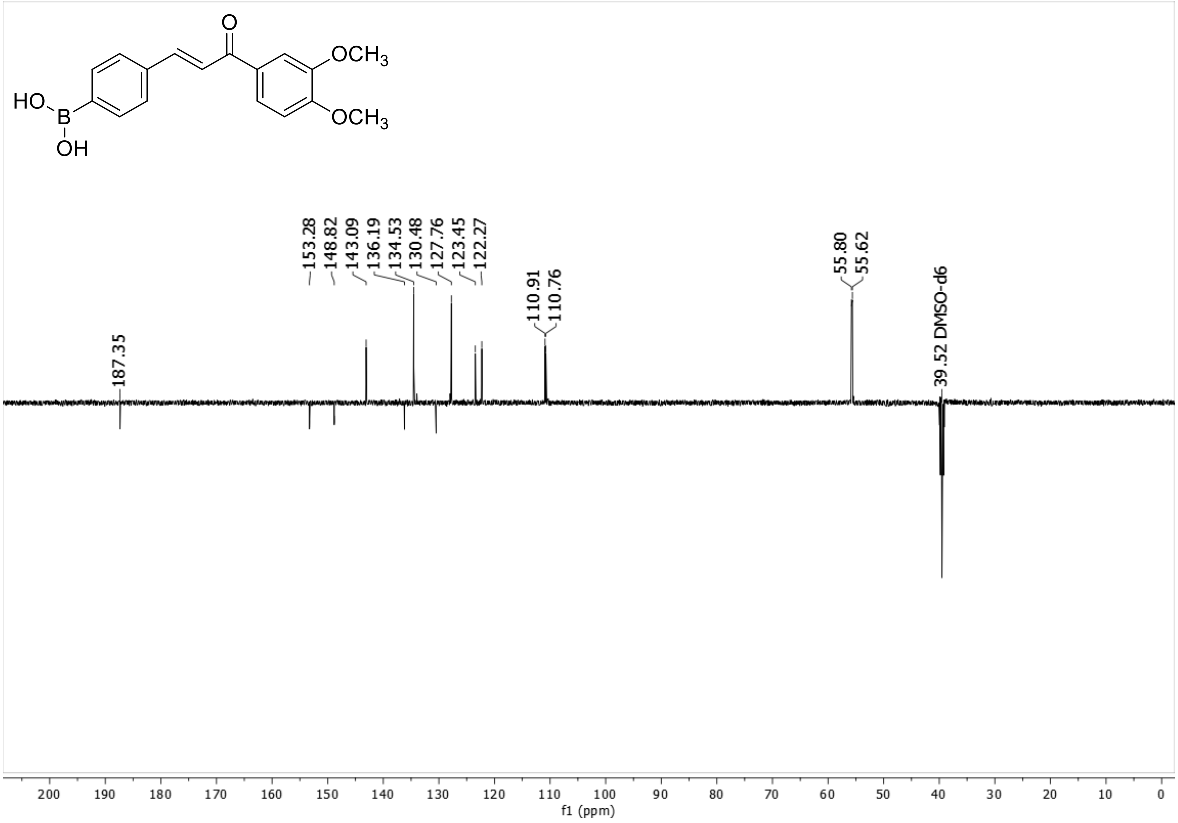

Figure S81. DEPTQ-NMR Spectrum of compound **12** (150 MHz DMSO-*d*6).

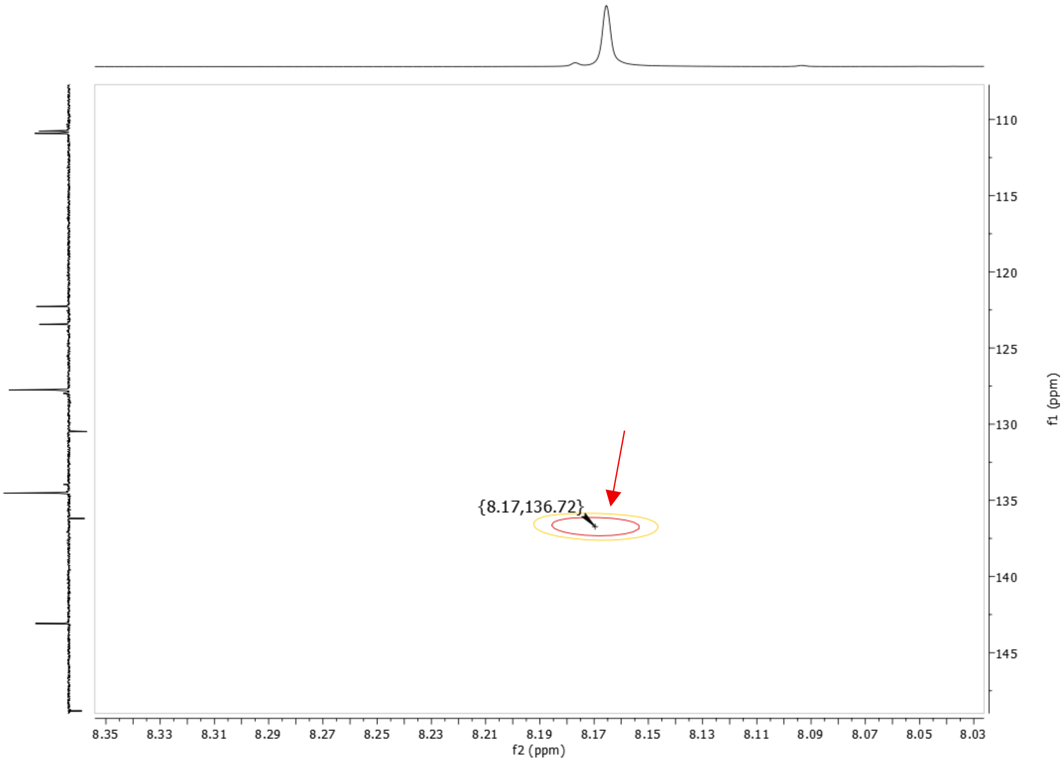

Figure S82. HMBC-NMR Spectrum of compound **12** (150 MHz DMSO-*d*6). The red arrow indicates the possible signal of the quaternary carbon directly bonded to boron (C–B).

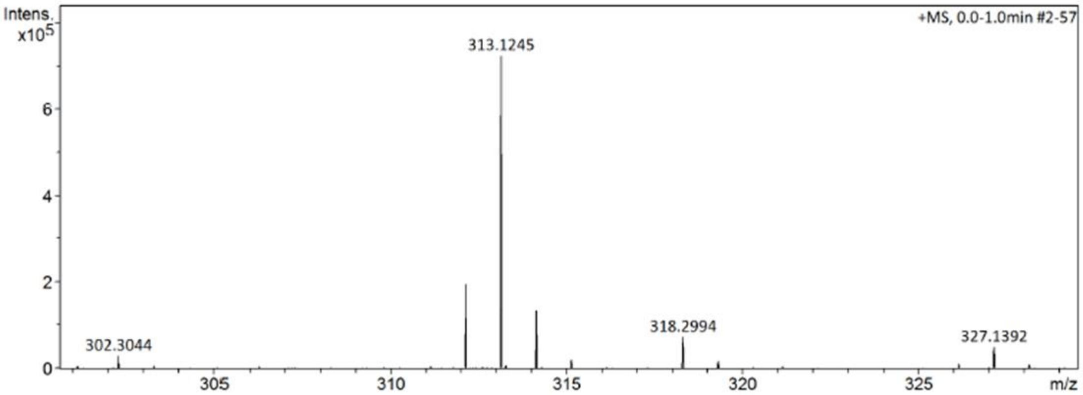

Figure S83. HRMS Spectrum of compound **12** (ESI+).

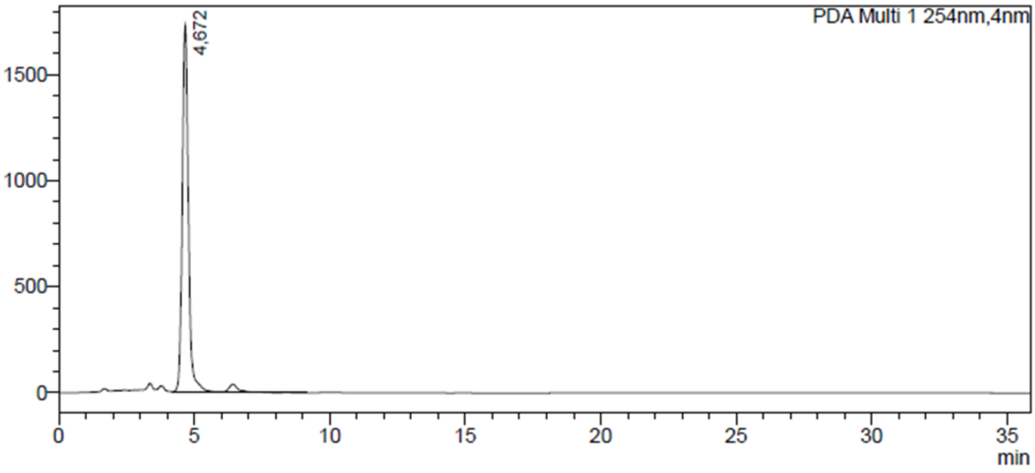

Figure S84. Chromatogram of compound **12**.

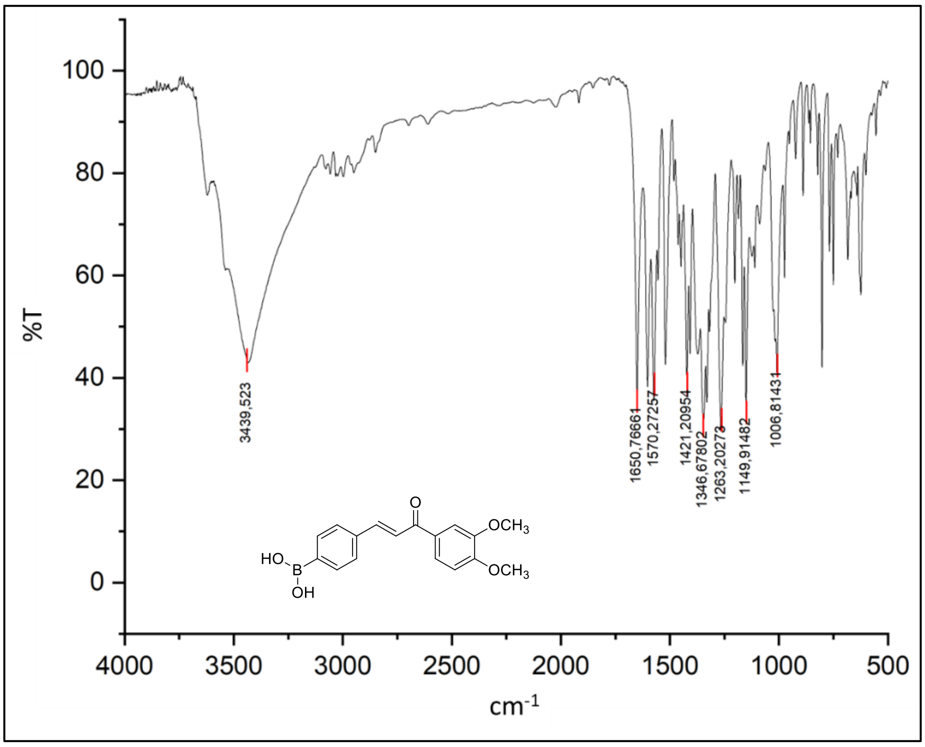

Figure S85. FTIR Spectrum of compound **12.**
